# Designing coastal conservation to deliver ecosystem and human well-being benefits

**DOI:** 10.1371/journal.pone.0172458

**Published:** 2017-02-27

**Authors:** Gust M. Annis, Douglas R. Pearsall, Katherine J. Kahl, Erika L. Washburn, Christopher A. May, Rachael Franks Taylor, James B. Cole, David N. Ewert, Edward T. Game, Patrick J. Doran

**Affiliations:** 1 The Nature Conservancy, Lansing, Michigan, United States of America; 2 The Nature Conservancy, St. Louis, Missouri, United States of America; 3 The Nature Conservancy, West End Brisbane, Queensland, Australia; University of Sydney, AUSTRALIA

## Abstract

Conservation scientists increasingly recognize that incorporating human values into conservation planning increases the chances for success by garnering broader project acceptance. However, methods for defining quantitative targets for the spatial representation of human well-being priorities are less developed. In this study we employ an approach for identifying regionally important human values and establishing specific spatial targets for their representation based on stakeholder outreach. Our primary objective was to develop a spatially-explicit conservation plan that identifies the most efficient locations for conservation actions to meet ecological goals while sustaining or enhancing human well-being values within the coastal and nearshore areas of the western Lake Erie basin (WLEB). We conducted an optimization analysis using 26 features representing ecological and human well-being priorities (13 of each), and included seven cost layers. The influence that including human well-being had on project results was tested by running five scenarios and setting targets for human well-being at different levels in each scenario. The most important areas for conservation to achieve multiple goals are clustered along the coast, reflecting a concentration of existing or potentially restorable coastal wetlands, coastal landbird stopover habitat and terrestrial biodiversity, as well as important recreational activities. Inland important areas tended to cluster around trails and high quality inland landbird stopover habitat. Most concentrated areas of importance also are centered on lands that are already conserved, reflecting the lower costs and higher benefits of enlarging these conserved areas rather than conserving isolated, dispersed areas. Including human well-being features in the analysis only influenced the solution at the highest target levels.

## Introduction

Conservation planning has traditionally been employed to identify and prioritize areas with high ecological value for conservation actions by drawing on principles of conservation biology and focusing on biological or ecological features such as rare or endemic species, areas of high species richness, or important habitat types [1], [2], [3], [4]. Such planning often seeks to achieve scientifically derived targets for the representation of conservation features in a system of reserves, though conservation actions often include a combination of land and water protection and ecosystem restoration activities. It is increasingly recognized that incorporating social data and human values into conservation planning improves the chances of successful conservation by both garnering broader project acceptance and potentially expanding benefits to include human well-being [5], [6], [7], [8], [9]. However, while the practice of incorporating social data into conservation planning is becoming more common [10], methods for identifying and defining meaningful targets for elements of human well-being are much less established. In particular, incorporating human well-being into commonly used conservation planning software like Marxan [11] typically requires not only geospatial data for mapping select components of human well-being, but also a means of establishing targets for their representation. Here we describe an approach that uses stakeholder surveys to identify regionally relevant components of human well-being and identify targets for their representation. Components of human well-being were identified and used not as costs or threats to conservation, but as features that could co-occur with or be enhanced through improved ecological conditions.

**Fig 1:**
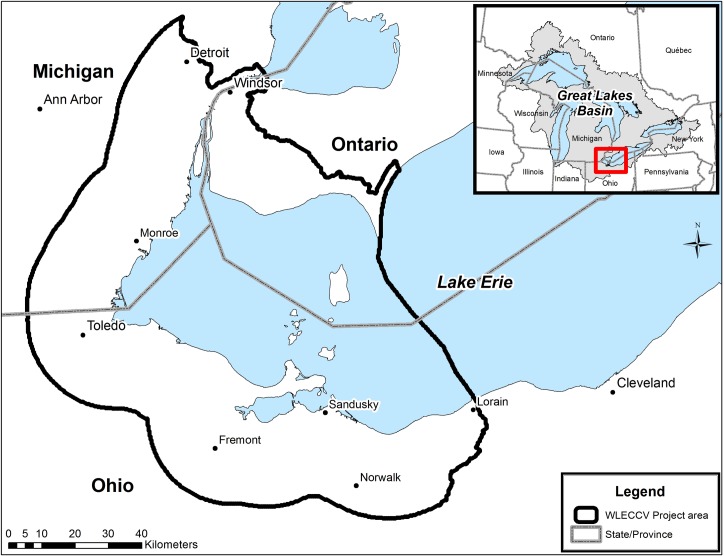
Project area. The black line designates the spatial extent of the Western Lake Erie Coastal Conservation Vision project area. The coastal portion of the western Lake Erie basin (up to 25k inland from coast) is based on supporting migratory stopover habitat data. Data credits: States/Provinces from U.S. States and Canada Provinces, Tele Atlas North America, Inc.; Cities from U.S. Cities, Data and maps for ArcGIS, ESRI; U.S. and Canada City points, Tele Atlas North America, Inc.; Lakes from Great Lakes GIS, Institute for Fisheries Research, Michigan Department of Natural Resources Fisheries Division and University of Michigan, School of Natural Resources; Great Lakes Basin from Great Lakes GIS, Institute for Fisheries Research, Michigan Department of Natural Resources Fisheries Division and University of Michigan, School of Natural Resources.

Our research focuses on the coastal and nearshore areas of the western Lake Erie basin (WLEB) (Fig 1) as a demonstration for combining ecological and social factors in conservation planning. Our primary objective was to develop a spatially-explicit conservation plan that identifies the most efficient locations for conservation actions to meet ecological goals while sustaining or enhancing human well-being values. First, we developed a process for integrating human well-being values into biodiversity conservation planning that can serve as a model both for other areas of the Great Lakes and conservation planning more generally. Second, we employed data not typically used in conservation planning and developed an innovative approach to incorporating social values which will benefit and complement priority-setting efforts across regional conservation, urban planning, and business sectors. Finally, we examined the influence that incorporating human well-being values into the conservation plan had in terms of: 1) the location and spatial extent of resulting solutions, and 2) the cost required to meet regionally-vetted ecological goals. The mapped outputs of this work comprise the Western Lake Erie Coastal Conservation Vision (WLECCV).

Our project results are not intended to suggest a system of reserves, but to highlight areas important for achieving regional ecological goals as well as contributing to important human well-being values. On-the-ground conservation practitioners will have to evaluate these resulting areas to determine what kind of protection, restoration, policy change, municipal planning, or other activities should be taken to best achieve regional goals. Worldwide, approximately 10% of the global population lives in low-lying coastal areas and faces increasing threats from climate change and other sources [12], and although this work was performed in the WLEB, the methods for meeting ecological targets and enhancing human well-being are transferrable to virtually any other coastal geography.

## Methods

### Study region

The western Lake Erie basin (WLEB) holds enormous ecological, cultural, and economic importance to local communities, visitors, and commercial interests that operate at regional and global scales. The WLEB is the warmest, shallowest, and most biologically productive region in the Laurentian Great Lakes of North America [13]. At the intersection of the Mississippi and North Atlantic Flyways, the WLEB is a critical corridor for birds migrating between breeding grounds in northern North America to wintering areas in the southern U.S.A. and as far as Central and South America. Food and shelter offered by the WLEB shoreline and inland stopover habitat is critical to millions of songbirds, waterfowl, shorebirds, hawks, owls and other species during the high-stress periods of spring and fall migration [14], [15]. Coastal ecosystems bordering the WLEB provide multiple ecosystem services [16] including hunting, migratory bird-watching opportunities, and world-renowned fishing which all contribute important economic revenues to the region. The nation’s largest spring birding festival is held in northwestern Ohio and generated $37 million in revenues in 2014 [17]. The recreational fishery generates $1.4 billion annually and the commercial fishery is worth $4.6 million in the U.S. and $33 million in Ontario [18]. In addition, the lake provides drinking water to approximately 11 million people [19]. The watershed of the WLEB also supports substantial and diverse agricultural production; of the roughly 9.1 million acres (3.7 million Ha; excluding Lake St. Clair) in the watershed, roughly 65% is in crop or pasture land [20], [21].

Despite these assets, the WLEB has been severely degraded due to high human population densities, intensive agriculture, and significant shoreline hardening [22], [16]. Anthropogenic impacts have degraded natural habitat and water quality, reduced native plant and wildlife populations, and diminished many ecological services. There is a resounding call to prioritize conservation action in the WLEB and the Great Lakes to help reverse some of these trends, particularly with unprecedented investments in restoration through the Great Lakes Restoration Initiative [23]. Conservation actions will need to meet measurable ecological goals and sustain the multiple nature-based activities that contribute positively to the region’s coastal communities and their economies. Since it is impossible to restore ecological function to the entire 240 km WLEB coastal region, conservation practitioners must determine which stretches of the coast are the highest priority for conservation activities that benefit both ecological systems and people.

In 2012, after a two-year binational planning process that included over 190 representatives from 87 agencies and organizations, the Lake Erie Biodiversity Conservation Strategy (LEBCS) [24] established ecological goals for protecting native biodiversity in and around Lake Erie in support of the Lake Erie Lakewide Action and Management Plan (LAMP) [13] for the first time. The LEBCS recommended several strategies for achieving these goals and general areas of biodiversity significance, but to date there has been no focused effort to identify specific areas for implementing those strategies. Our project area includes the Detroit River, the nearshore waters of the WLEB (which encompasses all of the open waters of western Lake Erie), and its coastal area up to 25k inland from the coast or the extent of the WLEB watershed, as defined in the (LEBCS) [24] (Fig 1). We focus on one of the LEBCS strategies–coastal conservation–and produce the first “conservation priority” map for the western Lake Erie basin that combines traditional ecological priorities with consideration of human well-being through a formal optimization process.

### Spatial optimization for site selection

We utilized Marxan with Zones [25], hereafter “MarxanZ”, to identify areas for conservation actions (including both protection and restoration, since restoration usually requires protection in the WLEB, and even protected lands often require restoration) that would benefit both ecological priorities and human well-being priorities. MarxanZ allows mapping of distinct spatial zones for different kinds of activities. Additionally, it offers the ability to incorporate multiple economic or social costs. The primary components of a MarxanZ analysis include a planning unit framework, a suite of features that represent conservation and human well-being priorities, and costs. Each of these components is described below.

### Planning unit framework

**Fig 2:**
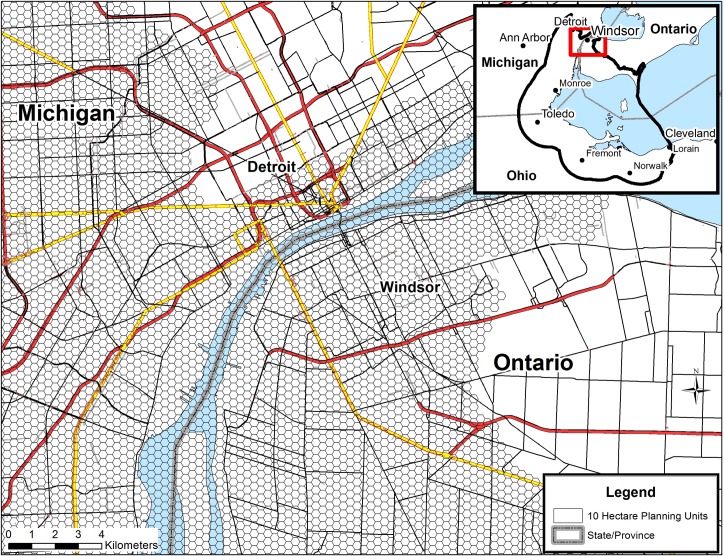
Showing 10-ha hexagon spatial planning units used in the WLECCV optimization analysis. Framework is shown here overlaid on the northern reach of the Detroit River, including portions of Michigan U.S.A. and Ontario, Canada. Data credits: States/Provinces from U.S. States and Canada Provinces, Tele Atlas North America, Inc.; Cities from U.S. Cities, Data and maps for ArcGIS, ESRI; U.S. and Canada City points, Tele Atlas North America, Inc.; Lakes from Great Lakes GIS, Institute for Fisheries Research, Michigan Department of Natural Resources Fisheries Division and University of Michigan, School of Natural Resources; Great Lakes Basin from Great Lakes GIS, Institute for Fisheries Research, Michigan Department of Natural Resources Fisheries Division and University of Michigan, School of Natural Resources; Roads from U.S. and Canada Major Roads, Tele Atlas North America, Inc.

Although there are no standardized methods for selecting planning units of a specific size and shape in a Marxan analysis, there are a number of factors typically considered. These include compatibility with the resolution of other input data, the scale of the planning, and the intended uses of the output products [26]. Many of our input data are represented by 30m or 100m grid cells. Local conservation actions are typically implemented on a parcel-specific basis and we wanted to use a planning unit that would be suitable for parcel level planning. We utilized 10-hectare (approximately 25-acre) hexagonal planning units (Fig 2, n = 120,197). This size is compatible with the resolution of our input data and represented a reasonable tradeoff between computing time and providing results at a scale meaningful for conservation actions. While smaller units might be ideal for local planning, the hexagons highlighted in our results can lead local planners to areas within which more detailed planning is warranted.

### Selecting ecological priorities, identifying conservation features and establishing targets

The Lake Erie Biodiversity Conservation Strategy (LEBCS) [24] established a set of ecological priorities and associated goals [13]; we generally adopted these ecological priorities with minor modifications. To spatially represent the ecological priorities as features in the MarxanZ analysis, we obtained and further processed data sets from multiple sources (see S1 Appendix and S1 Table for details). Most ecological priorities are represented by more than one conservation feature, and conservation targets for these features in the MarxanZ analysis were informed by the LEBCS goals and the relationship of the MarxanZ feature to the corresponding ecological priority. For example, fish habitat is a feature representing the nearshore system ecological priority, and the LEBCS does not specify a particular goal for fish habitat. In cases such as this, we assigned a target in MarxanZ by quantifying the extent of these features in the WLEB and using best professional judgement (Table 1). It should be noted that two conservation features, *Shorebird habitat* and *Inland waterfowl habitat*, have very low targets. These two features were included, despite being near desired target amounts, because they were identified as important by the LEBCS and our stakeholder surveys for the WLEB.

**Table 1:** Ecological priorities from the Lake Erie Biodiversity Conservation Strategy (LEBCS) [24] with corresponding conservation features and targets used in the Western Lake Erie Coastal Conservation Vision (WLECCV) project. Some conservation targets were modified from original LEBCS values based on more recent information.

Ecological Priorities	Conservation Features	Feature Description	Conservation Target
**Nearshore Zone: waters less than 15 m in depth, including the coastal margin**	Nearshore Fish Habitat	• Nearshore fish habitat data consisted of three depth based habitat classes: 1) walleye larval/juvenile habitat (highest potential impact and therefore highest priority for protection); 2) adult walleye habitat (second priority); and 3) walleye/yellow perch habitat (third priority). The amount of each habitat type was quantified for each planning unit. For target achievement full value was given to class 1, half value was given to class 2, and 1/3 value given to class 3. • Target based on professional judgement and consultation with fisheries experts.	10%
	Walleye Spawning Sites (lake)	• Number of spawning sites within each planning unit. • Target based on expert opinion of the importance of spawning sites for the persistence of walleye populations.	100%
**Native MigratoryFish: Lake Erie fish with populations that require tributaries for a portion of their life cycle, including lake sturgeon, walleye, suckers and sauger**	Walleye Spawning Sites (tributaries)	• Number of spawning sites within each planning unit. • Target based on expert opinion of the importance of spawning sites for the persistence of walleye populations.	100%
	Walleye Stream Potential Habitat	• Scores from 0 (none) to 100 (good) for the potential habitat suitability of streams for walleye. • Target based on professional judgement given the nature of the data (index) and the importance of stream habitat as expressed in the LEBCS.	25%
**Coastal Wetlands: wetlands with historic and current hydrologic connectivity to, and directly influenced by, Lake Erie**	Current and Restorable Coastal Wetlands	• Area of existing, former, or potential wetlands within each planning unit. • Target based on LEBCS goal of 10% increase in coastal wetland area and calculated from available data on current and restorable wetland extent.	20%
**Coastal Terrestrial Systems: upland systems within ~2 km of the shoreline**	Coastal Terrestrial Biodiversity Significance	• Index from 0 (none) to 21 (best) for the coastal biodiversity significance of each planning unit. • Target based on the LEBCS goals for coastal terrestrial systems and calculated from available data on biodiversity scores in the study area.	60%
**Aerial Migrants: migrating birds, insects, and bats dependent on the Lake Erie shoreline**	Coastal Landbird Habitat	• Area of high quality coastal landbird habitat within each planning unit. High quality defined as class 4 or 5.) • Target based on LEBCS goals for landbird stopover habitat and calculated from current data on high quality habitat area within the study area.	85%
	Inland Restorable Landbird Habitat	• Area of inland landbird habitat within each planning unit. Potentially restorable habitat includes agricultural lands as well as low intensity development lands. • Target based on LEBCS goals for landbird stopover habitat and calculated from current data on inland restorable habitat area within the study area.	30%
	Shorebird Habitat	• Area of high quality shorebird habitat within each planning unit (High quality defined as classes 3, 4, or 5). • Target based on goals in the Upper Mississippi River and Great Lakes Region Joint Venture Shorebird Habitat Conservation Strategy [27].	0.061%
	Nearshore Waterfowl Habitat	• Index from 0 (least important) to 9.72 (most important) of nearshore waterfowl habitat quality. • Target based on LEBCS goals for waterfowl stopover habitat and calculated from current data on waterfowl stopover habitat [28] within the study area.	30%
	Inland Waterfowl Habitat	• Index from 0 (least important) to 9.62 (most important) of inland waterfowl habitat quality. • Target based on goals in the Upper Mississippi River and Great Lakes Region Joint Venture Waterfowl Habitat Conservation Strategy [28].	0.8%
**Connecting Channels (Detroit River)**	Current and Restorable Coastal Wetlands	• Area of existing, former, or potential wetlands within each planning unit. • Target based on LEBCS goal of 10% increase in coastal wetland area and calculated from available data on current and restorable wetland extent.	20%
	Detroit River Spawning Sites (sturgeon, whitefish, walleye)	• Target based on expert opinion of the importance of spawning sites for the persistence of lake sturgeon, whitefish, and walleye.	100%
	Detroit River Walleye Habitat	• Scores from 0 (poor) to 100 (good) for the potential habitat suitability of streams for walleye. • Target based on professional judgement given the nature of the data (index) and the importance of stream habitat as expressed in the LEBCS.	25%
**Islands: including both naturally formed and artificial islands**	Coastal Terrestrial Biodiversity Significance	• Index from 0 (none) to 21 (best) for the coastal biodiversity significance of each planning unit. • Target based on the LEBCS goals for coastal terrestrial systems and calculated from available data on biodiversity scores in the study area.	60%

### Selecting human well-being priorities, identifying features and establishing targets

Whereas ecological priorities and associated targets were drawn from published reports, no corresponding, prioritized list of human well-being priorities and targets exists. To overcome this information deficit, we first adopted a framework of broad domains of human well-being. We then developed a conceptual diagram linking a set of ten ecosystem services found to be important in the WLEB and responsive to coastal conservation to the domains of human well-being. Finally, we identified relevant values through anthropological field work within local communities and reviews of county management plans throughout the study area. More specifically, our list of human well-being priorities was drawn from nine domains of human well-being, based on Smith et al. [29] and Lovelace et al. [30], including: health; social cohesion; education; safety and security; living standards; leisure time; spiritual and cultural fulfillment; life satisfaction and happiness; and connection to nature.

To identify locally important values, we conducted stakeholder interviews (n = 30) within a subset of the communities in our study area to garner information with respect to the social, economic, and cultural perspectives of people in the region. We conducted semi-structured, conversational interviews using maps as visual probes [31]. Interview participants were identified using a combination of ‘purposeful’ and ‘snowball sampling’ methods [32], [33]. Prospective interviewees were invited to participate via phone, email and in-person conversations. If interested, letters describing the interview protocol were sent via email or hard copy depending on the participant’s preference. The letter described the purpose of the interview, that participation was voluntary, that confidentiality would be maintained, financial compensation would not be provided, and that they could discontinue the interview for any reason, at any time. An author made arrangements with interviewees for a time and place that they preferred and, at the interview, the letter detailing the purpose was again reviewed allowing participants time to discuss questions and verbally consent. The interview was then conducted and, based on interviewee preference, recorded with a handheld digital recorder, handwritten notes, or both. Participants provided verbal consent to participate in the study. Consent was confirmed over the phone prior to arranging interviews via email and phone. At the interview we reviewed protocol and reconfirmed consent before initiating the interview. Written consent was deemed unnecessary because the interviews gathered information about human and community well-being, economic development, and the efforts underway in conservation and natural resource management. The interviews gathered information about well-being related to conservation and ecosystem services, not about human subjects. The Nature Conservancy’s World Office Human Subjects Board reviewed the study letter and the set of interview questions before use and approved of this consent procedure as the interviews were characterized as having no data about human subjects and no private information and that no federal funds were used to conduct this research. Qualitative analyses of these interviews revealed themes of social, cultural and economic challenges and opportunities for these communities with respect to their specific relationships to Lake Erie and the coastal environment.

We then constructed a conceptual diagram to associate the ten ecosystem services and the values identified through interviews and plan reviews with the nine domains of human well-being and evaluated how each of these might respond–positively or negatively–to coastal conservation activities. We eliminated from consideration those values and associated domains that were unlikely to respond, reducing the number of domains from nine to seven. We then found spatial data to represent five of the seven remaining domains, and had to eliminate two from the analysis for lack of data (Table 2). These data sets representing the human well-being priorities were developed into features for the MarxanZ analysis by attributing the data to the planning units; they were derived from multiple sources (Table 3; S1 Appendix and S1 Data Sources Table contains details on sources and development).

**Table 2:** Domains of human well-being used as an initial framework for the WLECCV (adapted from Smith et al. 2013 [29]).

Domain of Human Well-being	Definition	Affected (+ or -) by Coastal Conservation	Acquired Spatial Data
Health	Physical and psychological human health (behavior, mental and emotional health, nutrition and perceived health) + access to quality food and water, air quality	X	X
Social Cohesion	Bonds that tie people together in society (connectedness, identity, participation, trust and obligation, volunteering, city satisfaction, length of residence) + effective government, civil society, freedom of choice and action, social diversity, topophilia, tax revenue, groups / unions / associations	X	
Spiritual and Cultural Fulfillment	Opportunity to meet spiritual and cultural needs (importance of arts, culture and religion, purpose, visits to museums, natural and historic sites) + Recreational (cultural) places and activities	X	X
Education	Outcomes derived from formal and informal transfer of knowledge and skills (attainment, test results, participation, local knowledge and training)	X	
Safety and Security	Freedom from harm, both perceived and actual (violent crimes, safety at work and home, terrorism) + access to critical services		
Living Standards	Wealth, income levels, housing and food security (household and community debt, median home value, food availability and access, median income, poverty) + housing, economic security, equity, job satisfaction, property values, employment security	X	X
Life Satisfaction and Happiness	Contentment with our life (life evaluation, optimism and self-reported happiness) + personal well being		
Leisure Time	Amount and quality of time spent outside of obligations to work and home (time spent on hobbies, sporting events, relaxing, etc.)	X	X
Connection to Nature	The innate emotional affiliation of humans to other living organisms (respect and appreciation for nature) + Recreational (natural) places and activities, park lands, beach quality, scientific resources, coastal development, aesthetics	X	X

**Table 3:** Human well-being priorities, corresponding conservation features, and targets used in the WLECCV analysis, with corresponding domain(s) of human well-being (adapted from Smith et al. 2013 [29]). Only the target for Scenario 1 (highest level) is shown.

			**Domains of Human Well-being**					
**Human Well-being Priority**	Human Well-being Features	Feature Description	Health	Spiritual & Cultural Fulfillment	Living Standards	Leisure Time	Connection to Nature	Target
**Drinking water**	Drinking water intakes (Lake Erie)	Point locations of intake facilities in Lake Erie	X		X			94%
	Drinking water intakes (inland)	Point locations of intake facilities on land	X		X			94%
**Birding**	Birding visits; popularity of birding spots	Points attributed with number of unique visits, per day, at eBird designated hotspots	X			X	X	91%
**Fishing**	Recreational fishing (Lake Erie)	Polygons of state/provincial assessment units in Lake Erie attributed with fishing value in terms of angler-hours		X		X	X	89%
	Recreational fishing (streams)	Polygons in some OH rivers and the Detroit River attributed with fishing value in terms of angler-hours		X		X	X	89%
	Commercial fishing	Polygons of state/provincial assessment units in Lake Erie attributed with annual harvest in terms of lbs/km^2^			X			63%
**Hiking/Biking/Rec.**	Parks & recreation lands	Polygons of public lands managed as parks, conservation areas, wildlife refuges	X	X		X	X	89%
	Trails	Lines representing municipal, non-motorized trails	X	X		X	X	76%
**Swimming**	Beaches	Point locations of beaches	X	X		X	X	83%
**Boating**	Recreational boating	Raster; estimated density of boating use in the WLEB		X		X		72%
	Water access sites	Point locations of sites of public access to Lake Erie or tributary streams	X	X		X	X	72%
**Hunting**	Hunting areas	Polygons of public and private lands managed for hunting		X		X	X	70%
**Snorkeling/Diving**	Shipwrecks (dive sites)	Points locations of shipwrecks in Lake Erie attributed by distance from nearest marina (surrogate for frequency of use)		X		X		41%

Our review of county plans and stakeholder interviews offered little guidance on setting targets for these human well-being features. To assign targets, we established relative importance among the human well-being features by surveying regional stakeholders (n = 31 surveys; including representatives from state and federal agencies, community planners, conservation NGOs, and some private industries and businesses) at three workshops held in Monroe, Michigan, and Toledo, Ohio (both in the U.S.); and Essex, Ontario (Canada). Our survey approach was intended to ascertain a relative assessment of human well-being services within the western Lake Erie basin. The pool of participants was restricted to those who attended our workshops by invitation, including representatives from management, regulatory, and conservation agencies and organizations, and a few corporate and academic entities. We distributed a list of features and asked participants to rate their perception of the importance of each feature to people living in the region as “low”, “medium”, or “high”.

Internal Review Board (IRB) review is required for certain defined research activities which collect data on human subjects. The United States Department of Health and Human Services defines a “Human subject” as “a living individual about whom a research investigator (whether a professional or a student) obtains data through 1) intervention or interaction with the individual, or 2) identifiable private information” (46.102(f)). The Nature Conservancy’s inquiry was directed toward rating ecosystem or well-being values relevant to Western Lake Erie, and was not research on human subjects as defined above. The questionnaire asked participants to rate ten values (e.g. parks, trails, drinking water, commercial fishery, hunting) relevant to Lake Erie on the basis of two factors that could inform the importance of those values in selecting conservation projects. The questionnaire gathered information about those ecosystem values, not about human subjects. No data about human subjects and no private information were gathered through the questionnaire. No federal funds were used to conduct this research. For these reasons, no Internal Review Board review was required or sought.

After compiling scores across all three workshops, we normalized the scores on a scale of 0–100, 100 representing the score if every participant ranked the feature as high importance. We then scaled these scores by normalizing at four additional levels (0–75, 0–50, 0–25, and 0) and used these scores as targets (e.g., a score of 75 would indicate a target of 75%) to provide five scenarios for representing human well-being features (Table 4). These five scenarios enabled us to examine the influence of human well-being on the conservation plan, including when human well-being is omitted (targets all set to 0), representing a reserve design using only ecological priorities. Within each scenario we maintained the relative importance of each feature based on the results of the stakeholder surveys.

**Table 4:** Importance scores for human well-being features in the western Lake Erie basin. Scores based on workshop participants in OH, ON, and MI, and targets for those features at five levels obtained by normalizing the scores within varying ranges. We applied these five scenarios to understand how the human well-being features influence the overall conservation plan. Human well-being features that were added following the workshops, based on participant feedback, do not appear in this table; targets for those features were set based on relative similarity to other features.

		Scenario				
	Raw Workshop Score	1. (0–100)	2. (0–75)	3. (0–50)	4. (0–25)	5. (0)
**Birding**	85	91	69	46	23	0
**Parks and Recreation**	83	89	67	45	22	0
**Commercial Fishing**	59	63	48	32	16	0
**Recreational Fishing**	83	89	67	45	22	0
**Shipwrecks**	38	41	31	20	10	0
**Drinking Water**	87	94	70	47	23	0
**Beaches**	77	83	62	41	21	0
**Hunting**	65	70	52	35	17	0
**Recreational Boating**	67	72	54	36	18	0
**Trails**	71	76	57	38	19	0

### Costs

In our analysis, we also included a number of representative costs or other impediments to conservation actions. Much conservation planning has been based on crude proxies for costs [34]; however accurate costs are important to managers implementing on-the-ground activities. MarxanZ allows the use of multiple and sophisticated cost information in its optimization process. We identified seven cost layers, four of which are characterized in monetary values derived from local conservation projects, and three of which are cost indices reflecting landscape attributes that affect the feasibility of effective conservation (Table 5). Six of the seven costs apply to the inland and coastal portion of the study area; only the Lake Erie and Detroit River Stress Index applies to the Nearshore and Detroit River targets. Our analysis is primarily intended to optimize terrestrial conservation action, whereas in the nearshore area of Lake Erie and the Detroit River it is limited to identifying areas of importance without recommending particular actions in those two ecosystems. Hence, we do not incorporate monetary costs that would result from restrictions (e.g., marine reserves or limited take areas) within the lake. Datasets used to represent costs for this assessment were derived from multiple sources and are described in detail in S1 Appendix and S1 Table.

**Table 5:** Costs of implementing conservation actions in the coastal areas of western Lake Erie. S1 Appendix provides greater detail about each of these cost layers.

Cost	Description	Units
**Land value**	The average land value in the WLEB coastal area	$
**Wetland restoration**	The average cost of restoring coastal wetlands in the WLEB	$
**Phragmites treatment**	Cost estimate for removing the invasive common reed (*Phragmites australis*)	$
**Marinas**	Index representing marina size. Areas with marinas and lots of boat traffic would make coastal restoration more difficult.	Index
**Lake Erie and Detroit River Stress Index**	Index representing 34 stressors that likely have an impact on biota and ecosystem dynamics	Index
**Landbird habitat restoration**	Cost of restoring bird habitat based on land cover and the cost of planting trees	$
**Walleye stream habitat improvement cost**	Index representing the difficulty of restoring walleye habitat in streams	Index

### Innovative data

Our analysis was strengthened through the use and development of several innovative data sets, three of which we highlight here including current and restorable coastal wetland areas, crowd sourced data from eBird [35], and real-world costs for *Phragmites* treatment.

The Current and Restorable Coastal Wetlands feature (Table 2; S1 Appendix) incorporates novel data and recognizes the need for restoration as equal to or greater than the need for protection. This feature includes existing coastal wetlands [36] and a new data set from the Western Lake Erie Restoration Assessment (WLERA) that models the restoration potential for coastal areas on the US side of western Lake Erie [37]. The WLERA Version 1 incorporates a number of parameters and provides an index of wetland restorability. We used the footprint of areas with a restorability index > 0 to represent the area of potential wetlands, and other parameters to quantify the amount of current and restorable wetland area within each planning unit. We also used the restorability index in our wetland restoration cost layer (Table 5; S1 Appendix). This approach–focused on restoration in a highly degraded coastal area–is novel and now expanding northward via the Upper Midwest and Great Lakes Landscape Conservation Cooperative.

As a citizen science program, eBird [35] enables the public to keep an online checklist available for use with a smartphone application or the internet. Recreational and professional bird watchers record the method, location, and time of their birding trip, and then list the species heard/observed at that location. This data layer (Table 2; S1 Appendix) represents birding “hotspot” use as recorded by bird watchers. Hotspot locations are designated by a committee and defined as birding sites that are publicly accessible and likely to be birded regularly. We used the total number of eBird hotspot uploads from 2008–2012 to represent site popularity and birding as an ecosystem service.

The *Phragmites* treatment cost (Table 5; S1 Appendix) represents the real-world cost of treating the invasive common reed (*Phragmites australis*). The treatment method for removing *Phragmites* incorporates a three-year process with aerial spraying in the first year and follow-up spot removal of the remaining *Phragmites* during the next two years. Experienced practitioners at The Nature Conservancy estimate the cost for this three-year treatment at $500 per acre. Locations with *Phragmites* were identified from data developed by the Michigan Tech Research Institute [38]. The cost layer was constructed by multiplying each acre of *Phragmites* by $500.

### Zones

The WLECCV analysis area incorporates coastal and inland areas as well as large areas of water, especially the Detroit River and open waters of western Lake Erie. While specific conservation activities are well understood for land areas, they are not well established for the open waters of the Great Lakes (the Thunder Bay National Marine Sanctuary in Lake Huron is one exception). Since the WLECCV is addressing both land and water areas, we identified two distinct zones for our analyses: 1) a Terrestrial Conservation zone, defined as the terrestrial areas in the project area that are important for conservation actions; and 2) an Important Aquatic Areas zone, defined as lake and connecting channel waters which include areas important for biodiversity and cultural resources.

### Optimization analysis

MarxanZ produces numerous outputs [39]. Here we focus on two: the “best solution” and “selection frequency” outputs. The best solution output is from the single run (out of *n* runs where *n* is determined by the user) that best meets targets for the features and minimizes costs. It provides a traditional conservation plan with hard spatial boundaries; our 10-ha planning units are either “in” or “out” of the solution. It allows us to evaluate the different MarxanZ scenario levels used for the human well-being features, and to estimate the total area within which conservation actions would be required to meet targets for all features. In our analysis we decided to lock in existing protected lands because of the amount of ongoing conservation work in many of the areas. Locking these areas in recognizes the efforts of partners and will help characterize the importance of these areas for additional values that may not be of current focus.

The selection frequency output provides a count of the number of runs for which each planning unit is selected as part of the solution. In the selection frequency output, the planning units that are selected most frequently (e.g., >180 times out of 200 runs; the “top 10%”) are most critical for meeting targets and can be thought of as ‘irreplaceable’ planning units. In our analysis, these most critical planning units can be considered the highest priority for conservation or restoration actions. We used the selection frequency output to more precisely focus conservation actions on the ground than would be possible using the best solution. We also compare the “top 10 percent” of planning units among the five human well-being scenarios with respect to total area and cost.

## Results

Our results indicate optimal areas for meeting ecological and human well-being goals. On land, these areas should be interpreted as priorities for protection or restoration; determining specific conservation actions for individual areas will require field-scale assessments. We are not, however, suggesting actions such as restrictions on fishing or other activities for the important aquatic areas, only that these areas are important and contribute to both ecological and human well-being priorities. Improvement of these important aquatic areas might be achieved indirectly through terrestrial conservation efforts that would reduce sediment and nutrient inputs to Lake Erie, and future efforts may evaluate direct actions for these areas.

### Terrestrial conservation areas

**Fig 3:**
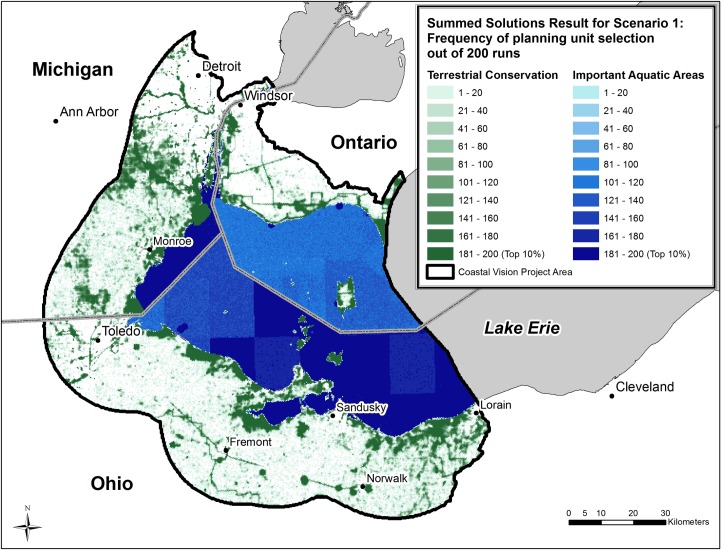
Results for Scenario 1. Targets based on workshop survey scores normalized to 100. Darker colors represent higher priority areas for conservation or restoration. Data credits: States/Provinces from U.S. States and Canada Provinces, Tele Atlas North America, Inc.; Cities from U.S. Cities, Data and maps for ArcGIS, ESRI; U.S. and Canada City points, Tele Atlas North America, Inc.; Lakes from Great Lakes GIS, Institute for Fisheries Research, Michigan Department of Natural Resources Fisheries Division and University of Michigan, School of Natural Resources.

The most important, or ‘irreplaceable’, areas for terrestrial conservation in Scenario 1 (highest human well-being level), cluster around the coast where many features representing both conservation and human well-being priorities are located, often co-occurring in the same planning units (Fig 3). In particular, the *current and restorable wetlands* and *coastal terrestrial biodiversity significance* features, which have relatively high targets, are located along the coast. Inland priority areas are primarily located in places with high *landbird stopover habitat* (representing Aerial Migrants) value and along *trails* (representing Health). Planning units that are included at lower frequencies for *landbird stopover habitat* are numerous and widespread, and because the target for *landbird stopover habitat* is high (85 percent along the coast and 30 percent of the entire inland project area), MarxanZ must include many of those planning units in each run to achieve the target.

### Important aquatic areas

The top 10 percent of important aquatic areas in Lake Erie are concentrated in the waters of Ohio and Michigan, corresponding to areas highly valued for *recreational fishing* and *recreational boating* as well as supporting numerous *walleye spawning areas* (Fig 3). *Recreational fishing* is of far greater importance to the solution in the U.S. than in Ontario, while *commercial fishing* is more important to the solution in Ontario. In accordance with evaluations completed in our stakeholder workshops, *recreational fishing* has a higher target in MarxanZ than *commercial fishing*, so the planning units with high *recreational fishing* value were selected more frequently than those with high *commercial fishing* value. Outside these areas of high *recreational fishing* and *recreational boating* value, frequently selected planning units mostly appear in a widespread, speckled pattern because the targets for most features in the lake can be achieved in many different areas. In addition, the international boundary and other boundaries between large grids used by state and provincial agencies for assessing both *commercial fishing* and *recreational fishing* effort are apparent in the lake. These boundaries and some of the speckling are the result of the relative homogeneity within the large grids and the high targets for features such as *nearshore fish habitat* and *nearshore waterfowl habitat*, both of which require a large lake area to achieve these targets.

### Impact of varying the targets for human well-being

**Fig 4:**
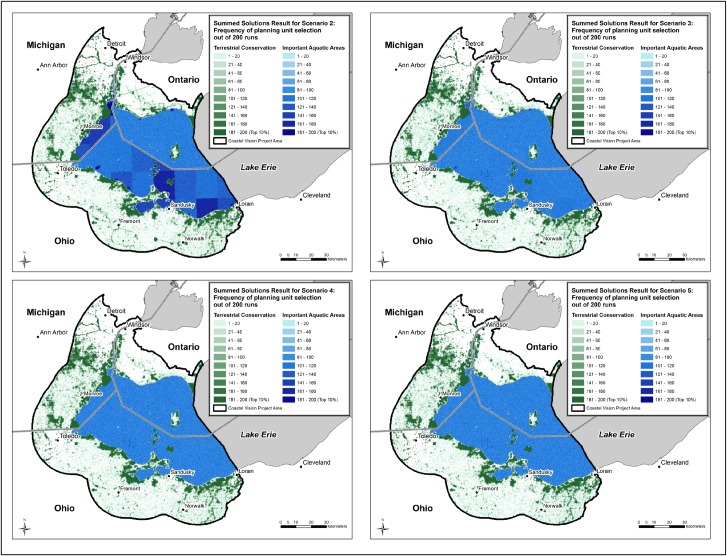
Results for Scenarios 2–5 based on four different human well-being target scenarios. Scenario 2 (targets based on workshop survey scores normalized to 75); Scenario 3 (targets based on workshop survey scores normalized to 50); Scenario 4 (targets based on workshop survey scores normalized to 25); Scenario 5 (targets based on workshop survey scores normalized to 0). Darker colors represent higher priority areas for conservation or restoration. Data credits: States/Provinces from U.S. States and Canada Provinces, Tele Atlas North America, Inc.; Cities from U.S. Cities, Data and maps for ArcGIS, ESRI; U.S. and Canada City points, Tele Atlas North America, Inc.; Lakes from Great Lakes GIS, Institute for Fisheries Research, Michigan Department of Natural Resources Fisheries Division and University of Michigan, School of Natural Resources.

**Fig 5:**
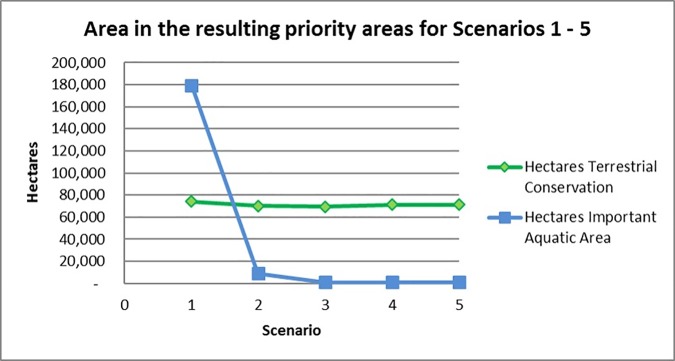
Area included in the resulting priority areas (top 10%) for Scenarios 1–5. Scenarios based on five different human well-being targets in both the Terrestrial Conservation Zone and the Important Aquatic Areas Zone. Scenario 1 (targets based on workshop survey scores normalized to 100); Scenario 2 (targets based on workshop survey scores normalized to 75); Scenario 3 (targets based on workshop survey scores normalized to 50); Scenario 4 (targets based on workshop survey scores normalized to 25); Scenario 5 (targets based on workshop survey scores normalized to 0).

Among the five optimization scenarios employing different targets for human well-being features, priority areas change only slightly (Figs 3 and 4). Correspondingly, the area of the top 10 percent planning units in the terrestrial conservation zone changes little across the five scenarios (Table 6). In contrast, the hectares of important aquatic area are substantially greater in Scenario 1 relative to the other scenarios (Fig 5). This dramatic increase in important aquatic area suggests that at lower targets many of the human well-being features are co-located and MarxanZ can achieve those targets with relatively little total area. Obviously some human well-being values are not co-located, so at the highest target setting (Scenario 1) Marxan must include many more planning units, each of which may contain just one or a few features.

**Table 6:** Hectares of terrestrial and important aquatic area in the resulting priority area (top 10%).

Scenario	Hectares Terrestrial Conservation	Hectares Important Aquatic Areas	Land Cost	Hectares Already Conserved	Percentage Already Conserved	Percentage of Total Land Area
**1.**	74,220	179,499	$20,074,746,989	38,090	51%	9.1%
**2.**	70,240	9,030	$17,352,902,060	38,090	54%	8.6%
**3.**	69,500	890	$16,522,838,978	38,090	55%	8.5%
**4.**	71,090	890	$16,922,768,163	38,090	54%	8.7%
**5.**	71,190	890	$16,912,965,806	38,090	54%	8.7%

Results based on five scenarios with varying human well-being targets.

**Fig 6:**
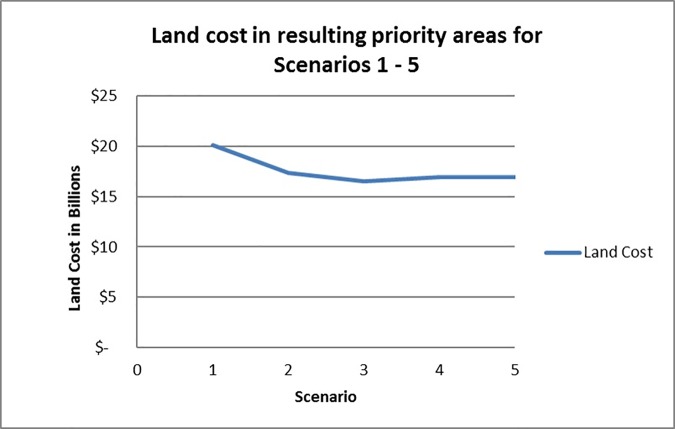
Land cost associated for Scenarios 1–5. Scenarios based on five different human well-being targets in both the Terrestrial Conservation Zone and the Important Aquatic Areas Zone. Scenario 1 (targets based on workshop survey scores normalized to 100); Scenario 2 (targets based on workshop survey scores normalized to 75); Scenario 3 (targets based on workshop survey scores normalized to 50); Scenario 4 (targets based on workshop survey scores normalized to 25); Scenario 5 (targets based on workshop survey scores normalized to 0).

In the terrestrial conservation zone, as targets for human well-being increase, the concentration of top 10% planning units shifts slightly from coastal to inland, reflecting greater relative importance of features such as trails and inland water intake facilities. The increase in area at the highest target levels is reflected in the land cost of the terrestrial conservation zone (Fig 6). Even though the increase in area between Scenarios 1 and 2 is slight (8.6% to 9.1%), conservation of these additional lands would cost an additional $2.7 billion dollars (Table 6, Fig 6), based on real land values in the WLEB; note that we are not suggesting that all land in the solutions would have to be purchased. In Scenario 1, achieving targets for human well-being requires more land and costlier lands; i.e., areas that may not contain as many ecological or human well-being features but that are required to achieve targets. Human well-being values are exerting more influence over the solution in Scenario 1, as is evident in the selection frequency map for the highest target setting (Fig 3); linear clusters of high-priority planning units–associated with trails–appear more distinctly in Ontario and northern Ohio than on any of the maps for Scenarios 2–5 (Fig 5). Water intake facilities in Ohio, many in proximity to trails, also are more prominent in the top 10% in Scenario 1. As these inland features become more critical for meeting higher targets, MarxanZ tends to select adjacent planning units that are less critical at lower targets. In this way, MarxanZ minimizes boundary costs, reflecting a preference for a few larger areas to many, smaller areas. Conversely, the top 10% solutions at the four lower target levels (Scenarios 2–5) apparently achieve targets for human well-being features without adding significant area or cost. In other words, human well-being values can be maintained or enhanced through conservation or restoration actions in these scenarios without increasing cost.

## Discussion

Conservation scientists and practitioners increasingly recognize that incorporating human values into conservation planning increases the chances for success by garnering broader project acceptance [5], [6], [7], [8]. However, methods for defining quantitative targets for the spatial representation of human well-being priorities are generally lacking [40]. Our approach to identifying regionally important human values, compiling datasets to represent them, and establishing specific targets based on stakeholder outreach and survey is novel and could be applied to other areas. Our methodology was a demonstration of how we might use MarxanZ to achieve multiple ecological goals, set through a rigorous stakeholder-supported target- and goal-setting process, and enhance human well-being. Future work should strive to use larger surveys with a more complete representation of stakeholders and employ more rigorous methods for socioeconomic or ecosystem services valuations.

A number of studies have used stakeholder feedback on human well-being factors to influence conservation plan objective setting ([41], [42], [43], [44]) though such efforts have generally not used stakeholder feedback to set quantitative spatial targets other than for commercial values, such as fish harvest (e.g., [39]). In our study, we describe a simple approach for using stakeholder surveys to generate relative importance among human well-being factors and translate that into spatial representation targets in a formal optimization process. We identified and used 13 human well-being features within five domains of well-being that could benefit from improved ecological conditions and show that by eliciting stakeholders’ perceived importance of each of these factors we can assign relative importance values and translate those into quantitative targets within a systematic conservation planning analysis using MarxanZ.

The results of this work demonstrate a method for identifying the best places for conservation actions that not only achieve regionally defined ecological goals but also incorporate important aspects of human well-being. Implementing conservation actions on 9% of the land area within 25 kilometers of the coast in the western Lake Erie basin will meet most ecological goals as established in the Lake Erie Biodiversity Conservation Strategy (LEBCS) [24] and approximately half of that land is already in some form of conservation ownership (although conservation ownership does not necessarily equate to good ecological condition). These same actions will also enhance many of the places people value for nature-based recreational activities and the provision of clean drinking water, therefore providing opportunities that enhance chances for conservation success. Although we are not advocating specific conservation actions in Lake Erie for the important aquatic areas it is assumed that conservation actions on the land will help reduce sediment and nutrient inputs to the lake thereby improving the aquatic habitats and ecological conditions.

In our analysis, varying targets for human well-being features while keeping ecological targets constant affected the resulting priority areas for terrestrial conservation only at the highest target level. That is to say, results for the four lower target levels for human well-being suggest that conservation investments in priority areas should also enhance human well-being values. This observed correspondence among ecological importance and human well-being may not carry over to other regions or to a different suite of conservation and human well-being priorities. We selected human well-being priorities based, in part, on a perceived benefit from conservation actions (as reported importance from LEBCS workshops and our three WLECCV workshops); if we had selected human well-being values that would not benefit from conservation or restoration, we may have observed a stronger influence of human well-being on the conservation plan.

While the maps produced through this project have the potential to serve as a “blueprint” for conservation actions in the region, it is important to note that they do not provide information on the specific type of actions needed on the ground (i.e., protection, restoration, other). Necessary actions could be clarified through more detailed inspection of underlying data sets (e.g., phragmites cost, land value), though in many cases local site evaluations will be necessary. Although we emphasize the utility of the resulting conservation priority map and data to conservation practitioners, it is important to point out that highlighted areas could also be selected by regional or community planners as sites for hiking, birding or other recreational uses that people associate with healthy and intact ecological systems; again, the underlying data could be used to inform such decisions.

Detailed maps, methods and supporting materials from this work can be found at www.nature.org/wlecoastal.

### Future work and lessons learned

We are just beginning to discuss on-the-ground applications of this WLECCV analysis with conservation partners and engaging local communities. By learning the “currencies” in which communities value their coastal resources, we can apply resulting (or new) data to inform current and future conservation actions toward more ecologically, economically and socially resilient futures. Ultimately, through stronger conservation partnerships and community engagement, we hope to quantify linkages between specific conservation actions and measurable human well-being impacts.

More work is needed to develop robust methods of identifying and assigning quantitative targets to human well-being priorities through stakeholder engagement, interviews, focus groups, in-person or web-based surveys, ecosystem service valuation, or crowd sourced data collection. In addition, more research and effort are needed to develop geospatial data representing social, cultural and economic services provided through healthy functioning ecosystems (i.e., ecosystem services). Gaps, in dollars and acres, have been assessed to state current “conditions” relative to WLEB-specific goals. In order to effectively track regional progress toward goals, we need regularly updated, high resolution spatial data to assess land cover change over time (e.g., changes in condition or extent of coastal wetlands) and an online tracking system, such as https://greatlakesinform.org/, to make goal status transparent to conservation practitioners, municipalities, funders and decision makers. These regular updates, coupled with spatial optimization, would facilitate efficient adaptive management of on-the-ground conservation actions, allowing conservation practitioners to readily identify remaining candidate locations where conservation can meet multiple objectives at the lowest cost [45].

## Supporting information

S1 Appendix Dataset Descriptions. Descriptions of and Datasets used to Represent Conservation Targets, Human Well-being Targets, and Costs.
(PDF)

S1 Table List of Data Sources Used. (PDF)

## References

[1] Margules CR, Pressey RL. Systematic conservation planning. Nature. 2000 5 11; 405(6783):243–53. 10.1038/35012251 10821285

[2] Pressey RL. Conservation planning and biodiversity: assembling the best data for the job. Conserv Biol. 2004 12 1;18(6):1677–81.

[3] Sowa SP, Annis G, Morey ME, Diamond DD. A gap analysis and comprehensive conservation strategy for riverine ecosystems of Missouri. Ecol Monogr. 2007 8; 77(3):301–34.

[4] Conservancy Nature. Conservation Action Planning Handbook: Developing Strategies, Taking Action and Measuring Success at Any Scale. The Nature Conservancy, Arlington, VA 2007. 127p.

[5] Knight AT, Cowling RM, Campbell BM. An operational model for implementing conservation action. Conserv Biol. 2006 4 1; 20(2):408–19. 16903102 10.1111/j.1523-1739.2006.00305.x

[6] Cowling RM, Wilhelm-Rechmann A. 2007. Social assessment as a key to conservation success. Oryx. 2007; 41(2): 135–136.

[7] Milner‐Gulland EJ, McGregor JA, Agarwala M, Atkinson G, Bevan P, Clements T, et al Accounting for the Impact of Conservation on Human Well‐Being. Conserv Biol. 2014 10 1;28(5):1160–6. 10.1111/cobi.12277 24641551 PMC4315902

[8] Stephanson SL, Mascia MB. Putting people on the map through an approach that integrates social data in conservation planning. Conserv Biol. 2014 10 1;28(5):1236–48. 10.1111/cobi.12357 25102957

[9] Pearsall DR, Khoury ML, Paskus J, Kraus D, Doran PJ, Sowa SP, et al Environmental Reviews and Case Studies: “Make No Little Plans”: Developing Biodiversity Conservation Strategies for the Great Lakes. Environ Pract. 2013 12 1;15(04):462–80.

[10] Balmford A, Cowling RM. Fusion or failure? The future of conservation biology. Conserv Biol. 2006 6 1;20(3):692–5. 16909557 10.1111/j.1523-1739.2006.00434.x

[11] Ball IR, Possingham HP, Watts ME. Marxan and relatives: software for spatial conservation prioritization Spatial conservation prioritization: quantitative methods and computational tools. Oxford University Press, Oxford 2009:185–95.

[12] McGranahan G, Balk D, Anderson B. The rising tide: assessing the risks of climate change and human settlements in low elevation coastal zones. Environ Urbanization. 2007 4 1;19(1):17–37.

[13] Lake Erie LaMP. 2008. Lake Erie Lakewide Management Plan: 2008 Update. Prepared by the Lake Erie LaMP Work Group. Available: https://www.epa.gov/sites/production/files/2015-10/documents/lake-erie-lamp-2008.pdf. Accessed 4.15.2015

[14] Ewert DN, Soulliere GJ, Macleod RD, Shieldcastle MC, Rodewald PG, Fujimura E, et al Migratory bird stopover site attributes in the western Lake Erie basin. Ann Arbor. 2006 4;1001:48103.

[15] Ewert DN, Doran PJ, Hall KR, Froehlich A, Cannon J, Cole JB, et al. On a wing and a (GIS) layer: prioritizing migratory bird stopover habitat along Great Lakes shorelines. Final report to the Upper Midwest/Great Lakes Landscape Conservation Cooperative. 2012 Nov. Available: http://www.greatlakeslcc.org/wp-content/uploads/2012/09/LCC-PhaseIStopover-Report_Final.pdf.http://glmigratorybirds.org

[16] Allan JD, Smith SD, McIntyre PB, Joseph CA, Dickinson CE, Marino AL, et al Using cultural ecosystem services to inform restoration priorities in the Laurentian Great Lakes. Front Ecol Environ. 2015 10;13(8):418–24.,

[17] American Bird Conservancy. Birding’s “Biggest Week” partners to help Cerulean Warbler and other migratory birds. American Bird Conservancy 2015 Available: https://abcbirds.org/article/birdings-biggest-week-partners-to-help-cerulean-warbler-and-other-migratory-birds/. March 19, 2015. Accessed April 27, 2015.

[18] Lake Erie Improvement Association. Strategic Plan for Lake Erie Partners: Sustaining Healthy Waters for Lake Erie’s Economy. 2012. Available: http://www.lakeerieimprovement.org/wp-content/uploads/2012/02/leia-strategic-plan-final-12-17-2012.pdf

[19] EPA. Lake Erie. EPA 2015 Available: http://www.epa.gov/greatlakes/lakeerie/. 2015-03-27. Accessed 4/24/2015.

[20] U.S. Department of Agriculture. 2014. 2012 Agricultural Census Report: Great Lakes Water Resource Region 04, HUC6 Level Watersheds. Available at https://www.agcensus.usda.gov/Publications/2012/Online_Resources/Watersheds/gl04.pdf

[21] Ontario Ministry of Natural Resources. 2002. Provincial Land Cover 2000. GIS data available at https://www.javacoeapp.lrc.gov.on.ca/geonetwork/srv/en/main.home?uuid=ed66d203-d5ca-47a2-b357-0226ea3d29ae Accessed 24 May 2014.

[22] Allan JD, McIntyre PB, Smith SD, Halpern BS, Boyer GL, Buchsbaum A, et al Joint analysis of stressors and ecosystem services to enhance restoration effectiveness. Proceedings of the National Academy of Sciences. 2013 1 2;110(1):372–7. 10.1073/pnas.1213841110 PMC3538252 23248308

[23] Great Lakes Interagency Task Force. Great Lakes Restoration Initiative Action Plan II. Great Lakes Restoration Initiative (GLRI). 2014 Available: http://greatlakesrestoration.us/actionplan/index.html

[24] Pearsall D, Carton de Grammont P, Cavalieri C, Chu C, Doran P, Elbing L, et al. *Returning to a Healthy Lake*: *Lake Erie Biodiversity Conservation Strategy*. Technical Report. A joint publication of The Nature Conservancy, Nature Conservancy of Canada, and Michigan Natural Features Inventory, Lansing, MI, 340 pp. with appendices; 2012.

[25] Watts ME, Ball IR, Stewart RS, Klein CJ, Wilson K, Steinback C, et al Marxan with Zones: software for optimal conservation based land-and sea-use zoning. Environmental Modelling & Software. 2009 12 31;24(12):1513–21.,

[26] Ardron JA, Possingham HP, Klein CJ. Marxan good practices handbook, Version 2. Pacific Marine Analysis and Research Association, Vancouver, BC, Canada 2010.

[27] Potter BA, Gates RJ, Soulliere GJ, Russell RP, Granfors DA, Ewert DN. Upper Mississippi River and Great Lakes Region joint venture shorebird habitat conservation strategy. US Fish and Wildlife Service, Fort Snelling, MN 2007.

[28] Soulliere GJ, Potter BA, Coluccy JM, Gatti RC, Roy CL, Luukkonen DR, et al Upper Mississippi River and Great Lakes region joint venture waterfowl habitat conservation strategy. US Fish and Wildlife Service, Fort Snelling, Minnesota, USA 2007.

[29] Smith LM, Case JL, Smith HM, Harwell LC, Summers JK. Relating ecoystem services to domains of human well-being: Foundation for a US index. Ecol Indic. 2013 5 31;28:79–90.

[30] Lovelace S, Goedeke TL, Dillard M. 2011. Prioritizing County-Level Well-Being: Moving Toward Assessment of Gulf Coast Counties Impacted by the Deepwater Horizon Industrial Disaster. NOAA Technical Memorandum NOS NCCOS 146, 48 pp.

[31] Washburn EL. A social landscape analysis of land use decision making in a coastal watershed. Soc Nat Resour. 2013 3 1; 26(3):325–38.

[32] Leach WD. Surveying diverse stakeholder groups. Society & Natural Resources. 2002 8 1;15(7):641–9.

[33] MacMynowski DP. Across space and time: Social responses to large-scale biophysical systems. Environ Manage. 2007 6 1;39(6):831–42. 10.1007/s00267-006-0082-4 17415612

[34] Groves C, Game E. Conservation Planning: Informed Decisions for a Healthier Planet. Roberts & Company Press; 2015.

[35] eBird. 2013. eBird: An online database of bird distribution and abundance [web application]. eBird, Ithaca, New York. Available: http://www.ebird.org. (Accessed: May 2013).

[36] Albert DA, and Simonson L. 2004 Coastal wetland inventory of the Great Lakes basin (GIS coverage of the entire U.S. Great Lakes), Great Lakes Coastal Wetlands Consortium, Great Lakes Commission, Ann Arbor, Mich.

[37] Saarinen JA, Kowalski KP. 2015. Western Lake Erie Restoration Assessment (WLERA), version 1.0. Unpublished data.

[38] Bourgeau-Chavez L, Endres S, Battaglia M, Miller ME, Banda E, Laubach Z, et al Development of a bi-national Great Lakes coastal wetland and land use map using three-season PALSAR and Landsat imagery. Remote Sensing. 2015 7 9;7(7):8655–82. http://www.mdpi.com/2072-4292/7/7/8655

[39] Watts ME, Steinback C, Klein C. User Guide: Applying Marxan with Zones, North central coast of California marine study. University of Queensland, Brisbane 2008.

[40] Ban NC, Mills M, Tam J, Hicks CC, Klain S, Stoeckl N, et al A social–ecological approach to conservation planning: embedding social considerations. Front Ecol Environ. 2013 5 1;11(4):194–202.

[41] Klein CJ, Steinback C, Watts M, Scholz AJ, Possingham HP. Spatial marine zoning for fisheries and conservation. Front Ecol Environ. 2010 9 1;8(7):349–53.

[42] Grantham HS, Agostini VN, Wilson J, Mangubhai S, Hidayat N, Muljadi A, et al A comparison of zoning analyses to inform the planning of a marine protected area network in Raja Ampat, Indonesia. Marine Policy. 2013 3 31;38:184–94.

[43] Adams VM, Pressey RL, Stoeckl N. Navigating trade-offs in land-use planning: integrating human well-being into objective setting. Ecol Soc. 2014;19(4).

[44] Gurney GG, Pressey RL, Ban NC, Álvarez‐Romero JG, Jupiter S, Adams VM. Efficient and equitable design of marine protected areas in Fiji through inclusion of stakeholder‐specific objectives in conservation planning. Conserv Biol. 2015 10 1;29(5):1378–89. 10.1111/cobi.12514 25916976

[45] Game ET, Lipsett‐Moore G, Hamilton R, Peterson N, Kereseka J, Atu W, et al Informed opportunism for conservation planning in the Solomon Islands. Conserv Lett. 2011 2 1; 4(1):38–46.

