# Supplementary material for: Designing coastal conservation to deliver ecosystem and human well-being benefits
Source: PLoS One. 2017 Feb 27;12(2):e0172458. doi: 10.1371/journal.pone.0172458 (PMC5328270; doi:10.1371/journal.pone.0172458)
Supplement: S1 Appendix — Descriptions of and Datasets used to Represent Conservation Targets, Human Well-being Targets, and Costs. (PDF) [file pone.0172458.s001.pdf]

S1 Appendix:
Descriptions of and Datasets used to Represent Conservation Targets, Human Well-being Targets, and Costs

Supporting information (S1 Appendix) to manuscript:

Designing coastal conservation to deliver ecosystem and human well-being benefits

Gust M. Annis[1*], Douglas R. Pearsall[1], Katherine J. Kahl[1], Erika L. Washburn[1,#a], Christopher A. May[1], Rachael Franks Taylor[1,#b], James B. Cole[2], David N. Ewert[1], Edward T. Game[3], Patrick J. Doran[1]

Contents

Conservation Targets: 3
- Spawning Sites 3
- Walleye Stream Potential Habitat 6
- Nearshore Fish Habitat 9
- Current and Restorable Coastal Wetlands 11
- Coastal Terrestrial Biodiversity Significance 14
- Migratory Bird Stopover Habitat 16

Human Well-being Targets: 19
- Beaches 19
- Birding Visits 21
- Commercial Fishing 23
- Surface Water Intakes 25
- Hunting Areas 27
- Parks and Recreation Lands 29
- Recreational Boating 31
- Shipwrecks (Dive Sites) 33
- Recreational Fishing 35
- Trails 38
- Water Access Sites 40

Costs: 42
- Land Value 43
- Wetland Restoration Cost 45
- Phragmites Treatment Cost 47
- Marinas Cost to Wetlands 48
- Lake Erie and Detroit River Stress Index 49
- Landbird Habitat Restoration Cost 50
- Walleye Stream Habitat Restoration Cost 52

Conservation Targets:

Spawning Sites

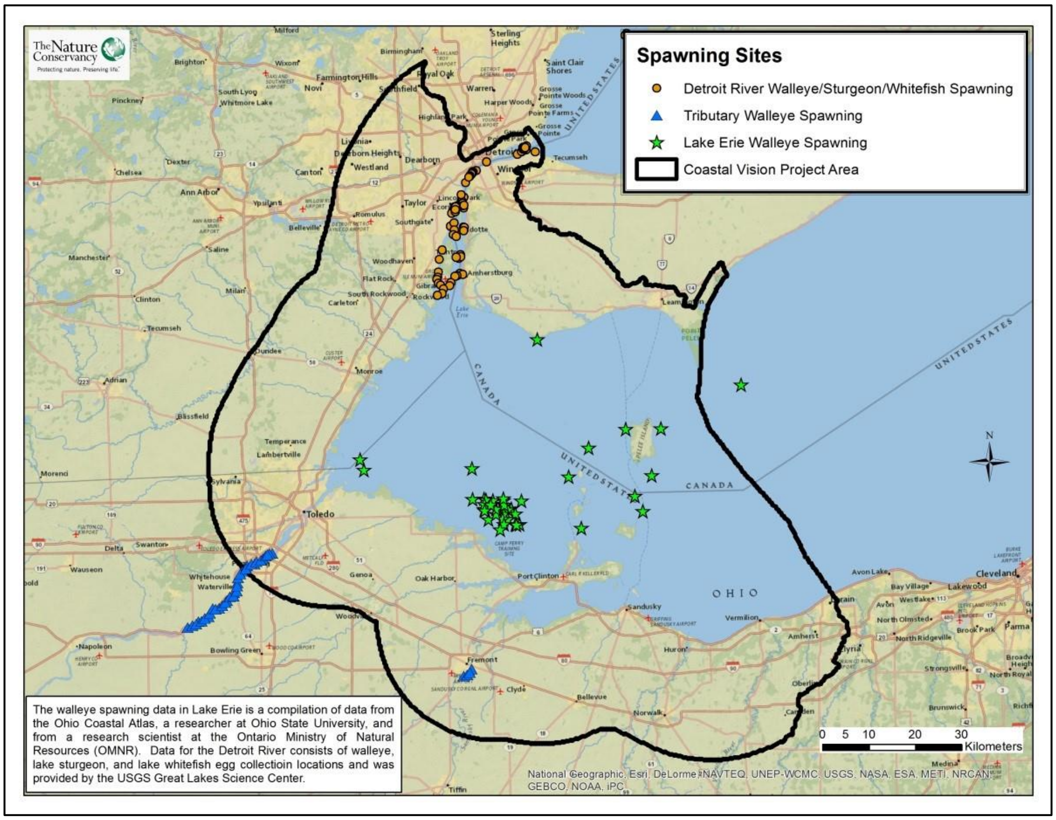

Spawning Sites. *Spawning locations for walleye, lake whitefish, and lake sturgeon.*

Fish spawning sites in relation to regional ecological and social values

This data layer includes spawning sites in three categories recognizing the importance of these distinct areas for different species or populations of fish. Walleye (*Sander vitreus*) spawning sites are mapped separately for the nearshore area of the Western Lake Erie Basin (WLEB) and in tributaries. Detroit River spawning sites are mapped as important to three species including walleye, lake whitefish (*Coregonus clupeaformis)*, and lake sturgeon (*Acipenser fulvescens*). All three species are important native migratory fish. Walleye are important native predators and as migratory fish they aid in the migration of other species like mussels. Walleye, like many native fish, spawn in tributaries and in shallow, rocky shoals in the lake. The stability of migratory fish populations is therefore threatened by dams and other obstructions that prevent fish from completing their migration from the lake to upstream areas[1]. Within the lake, spawning success can be affected by boats passing overhead, which disturbs spawning fish by increasing water movement, and by kicking up sediments that can interfere with spawning success and egg survival[2]. Taking care to minimize boating disturbance in order to maintain healthy fish populations allows for the proper functioning of the ecosystem and also directly benefits boaters themselves; Lake Erie is the most popular sport fishing destination in the Great Lakes basin, and would collapse

immediately if walleye and other sport fish populations crashed. According to a survey by the USFWS, approximately 646,000 Great Lakes anglers focused their efforts on Lake Erie in 2011, and nearly a third of these visitors came in search of sauger and walleye[3]. Walleye, along with yellow perch (*Perca flavescens*), also account for 80% of the CA$33 million Canadian commercial fishing industry[4]. Lake whitefish, once virtually extirpated from the WLEB, have recently been documented as spawning in the Detroit River. Their return to the system provides a good indicator of recovery. Lake sturgeon is a large, charismatic species listed as either threatened or endangered by 19 or the 20 states within its original range in the United States. Sturgeon spawn in the gravel beds of fast flowing streams, such as the Detroit and St. Clair Rivers and, prior to widespread construction of dams and other barriers, spawned in several other WLEB tributaries. The WLECCV project uses walleye, lake whitefish, and lake sturgeon as a proxy for migratory fish and incorporates their spawning areas in recognition of the importance of these habitats for supporting a healthy ecosystem and strong fishery in Lake Erie.

Spawning sites data layer

Fish spawning sites are identified by the Lake Erie Biodiversity Conservation Strategy (LEBCS) as an important target for conservation in the WLEB; the LEBCS sets forth a goal of having at least two viable populations of each migratory fish species present in each basin of Lake Erie. This data layer is a compilation of data from the Ohio Coastal Atlas, the USGS Great Lakes Science Center, a researcher at Ohio State University, and from a research scientist at the Ontario Ministry of Natural Resources (OMNR) (See "Data Sources"). Data obtained from the Ohio Coastal Atlas was originally gathered by the Ohio Department of Natural Resources Sandusky Fisheries Research Unit. Spawning data received from the OMNR represent areas where OMNR scientists have suggested spawning areas exist based on walleye tagged in the area during spawning seasons in past years. There were 170 spawning sites identified within the study area. Detroit River spawning sites were attributed with the number of species found (maximum of 3) at each site and then summing these numbers for all sites within a 10 hectare planning unit. For tributaries and nearshore spawning sites, values were derived by summing the number of spawning sites within each 10 hectare planning unit.

Data sources and potential limitations

Data for Ohio were downloaded from the Ohio Coastal Atlas website. Additional data for Ohio was provided by Cassie May, a PhD student at Ohio State University[5]. Data for Ontario were received from Dr. Yingming Zhao with the Ontario Ministry of Natural Resources[6]. Data for the Detroit River were provided by Dr. Edward Roseman and Jason Fischer at the USGS Great Lakes Science Center[7]. It would be ideal to include other migratory species in this analysis to ensure that the LEBCS goal for all migratory fish species is considered during the analysis; such data is currently not available for the WLEB, although a current mapping project underway at The Nature Conservancy may make it available in the future. This data layer includes many spawning locations in the WLEB, but it should be noted that unknown spawning areas may exist beyond the currently recorded areas.

References and links

1. Pearsall, D., P. Carton de Grammont, C. Cavalieri , C. Chu, P. Doran, L. Elbing, D. Ewert, K. Hall, M. Herbert, M. Khoury, D. Kraus, S. Mysorekar, J. Paskus and A. Sasson. (2012). *Returning to a Healthy Lake: Lake Erie Biodiversity Conservation Strategy*. Technical Report. A joint publication of The Nature Conservancy, Nature Conservancy of Canada, and Michigan Natural Features Inventory. 340 pp. with appendices. http://www.conservationgateway.org/ConservationByGeography/NorthAmerica/wholesystems/greatlakes/Pages/lakeerie.aspx
2. Asplund, T. 2000. The effects of motorized watercraft on aquatic ecosystems. Wisconsin Department of Natural Resources, Bureau of Integrated Science Services and Wisonsin-Madison Water Chemistry Program. http://files.dnr.state.mn.us/aboutdnr/reports/boating/impacts_mississippi_2004/references/asplund_2000.pdf
3. U.S Department of the Interior, U.S. Fish and Wildlife Service, and U.S. Department of Commerce, U.S. Census Bureau. 2011 National Survey of Fishing, Hunting, and Wildlife-Associated Recreation. www.census.gov/prod/2012pubs/fhw11-nat.pdf
4. http://www.mnr.gov.on.ca/en/Business/GreatLakes/2ColumnSubPage/STEL02_173913.html#Commercial_Fisheries
5. Cassie May, Graduate Student, PhD, Ohio State University.
6. Dr. Yingming Zhao, Research Scientist, Aquatic Research and Development Section, Ontario Ministry of Natural Resources.
7. Dr. Edward Roseman, Research Fishery Biologist, USGS Great Lakes Science Center.

Walleye Stream Potential Habitat

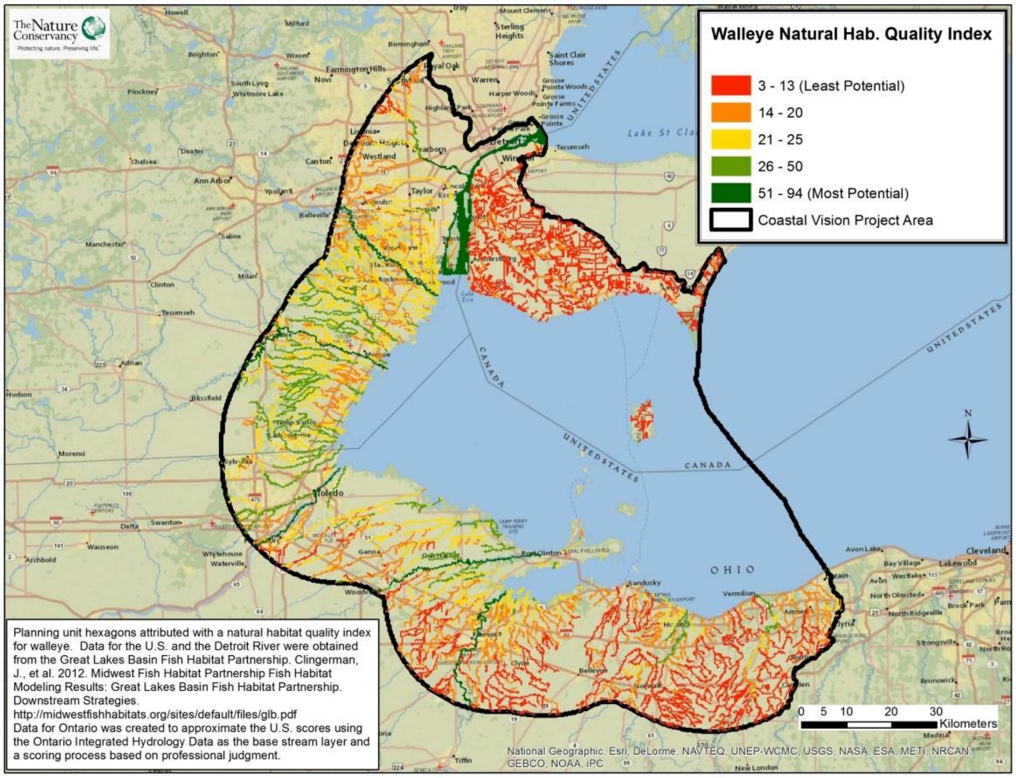

Walleye Stream Potential Habitat: *The WLEB analysis area is outlined in black, with the habitat quality score for each stream indicated by a color gradient from red (low quality) to green (high quality).*

Walleye stream potential habitat in relation to regional ecological and social values

This data layer uses walleye stream potential habitat as a proxy to represent all native migratory fish in the Western Lake Erie Basin (WLEB). Walleye (*Sander vitreus*) and other sport fish species are worth tens of millions (US$) to the WLEB economy; walleye and sauger are especially popular, luring 0.6 million of the 1.7 million anglers who contributed US$1.9 billion to the Great Lakes basin's economy in 2011[1]. There are at least 30 species supplying the fishing industry in the Lake Erie basin that rely on streams for spawning; it is essential to preserve the accessibility and overall quality of tributary spawning habitat in order to sustain these fish populations[2]. The vital connection between upstream habitats and Lake Erie has been difficult to maintain due to the installation of hydroelectric dams, poorly designed road-stream crossings, and various structures to alleviate storm and erosion damage, including dams and sea walls[3]. Other threats to the quality of stream habitats include non-point source pollution from agricultural and urban land, the contamination of sediments with toxic compounds, the presence of competitive or parasitic invasive species (e.g. sea lamprey), and temperature increases from climate change[4]. The Western Lake Erie Coastal Conservation Vision Project recognizes the ecological and socioeconomic importance of maintaining healthy stream habitat for fish populations like walleye, and therefore includes this data layer to ensure that stream habitat quality is incorporated into the optimization of areas for conservation.

Walleye stream habitat layer

The Lake Erie Biodiversity Conservation Strategy (LEBCS) established a goal of having at least 50% of each stream type– based on the Midwest Fish Habitat Partnership classification[5]–connected to Lake Erie in order to provide access to spawning areas for migratory fish[6]. The WLECCV includes this data layer to help stakeholders achieve the LEBCS goal by ensuring that the highest predicted quality stream habitat is included when determining optimal areas for conservation. To create this data layer for the U.S., the National Fish Habitat Partnership (NFHP) assessed fish habitat and identified 19 natural habitat characteristics that were then used to calculate potential habitat quality for walleye species in particular. Natural habitat characteristics that most influenced potential habitat quality were the drainage area of a stream's watershed, wetland area in the watershed, mean annual precipitation, modeled stream temperature, and slope of the watershed.

Data for the Ontario side of the basin was created to approximate the U.S. index scores using the Ontario Integrated Hydrology data as the base stream layer and a scoring process based on professional judgment. Based on personal communication from Ontario fisheries professionals at a stakeholder workshop held in Essex, Ontario, in October of 2014 all of the streams in this portion of Ontario should receive a "poor" score for walleye habitat. We assigned habitat scores based on steam size by assigning headwater streams a score of 5; creeks a score of 10; and small rivers a score of 15.

Data sources and potential limitations

The data used for this layer on the U.S. side of the basin were obtained from the Great Lakes Basin Fish Habitat Partnership[7]. It should be noted that while walleye are an important migratory species in the WLEB, it would be ideal to include other migratory species in this analysis; such data is currently not available for the WLEB, although a current mapping project underway at The Nature Conservancy may make it available in the future. In addition, the walleye habitat index scores developed for Ontario streams are only an approximation and were not developed with as much rigor as the Great Lakes Basin Fish Habitat Partnership data available in the U.S.

References and Links

1. 2011 National Survey of Fishing, Hunting, and Wildlife-Associated Recreation. August 2012. USFWS. http://www.doi.gov/news/pressreleases/upload/FWS-National-Preliminary-Report-2011.pdf
2. Pearsall, D., et al. 2012. *Returning to a Healthy Lake: Lake Erie Biodiversity Conservation Strategy*. Technical Report. A joint publication of The Nature Conservancy, Nature Conservancy of Canada, and Michigan Natural Features Inventory. 340 pp. with Appendices. http://www.conservationgateway.org/ConservationByGeography/NorthAmerica/UnitedStates/michigan/Pages/lakeerie.aspx
3. Clingerman, J., et al. 2012. Midwest Fish Habitat Partnership Fish Habitat Modeling Results: Great Lakes Basin Fish Habitat Partnership. Downstream Strategies. http://midwestfishhabitats.org/sites/default/files/glb.pdf
4. Pearsall, D., et al. 2012. *Returning to a Healthy Lake.*
5. Clingerman, J., et al. 2012. Midwest Fish Habitat Partnership Fish Habitat Modeling Results: Great Lakes Basin Fish Habitat Partnership. Downstream Strategies.

http://midwestfishhabitats.org/sites/default/files/glb.pdf 6. Pearsall, D., et al. 2012. *Returning to a Healthy Lake: Lake Erie Biodiversity Conservation Strategy*.

7. Clingerman, J., et al. 2012. Midwest Fish Habitat Partnership Fish Habitat Modeling Results: Great Lakes Basin Fish Habitat Partnership. Downstream Strategies. http://midwestfishhabitats.org/sites/default/files/glb.pdf

Nearshore Fish Habitat

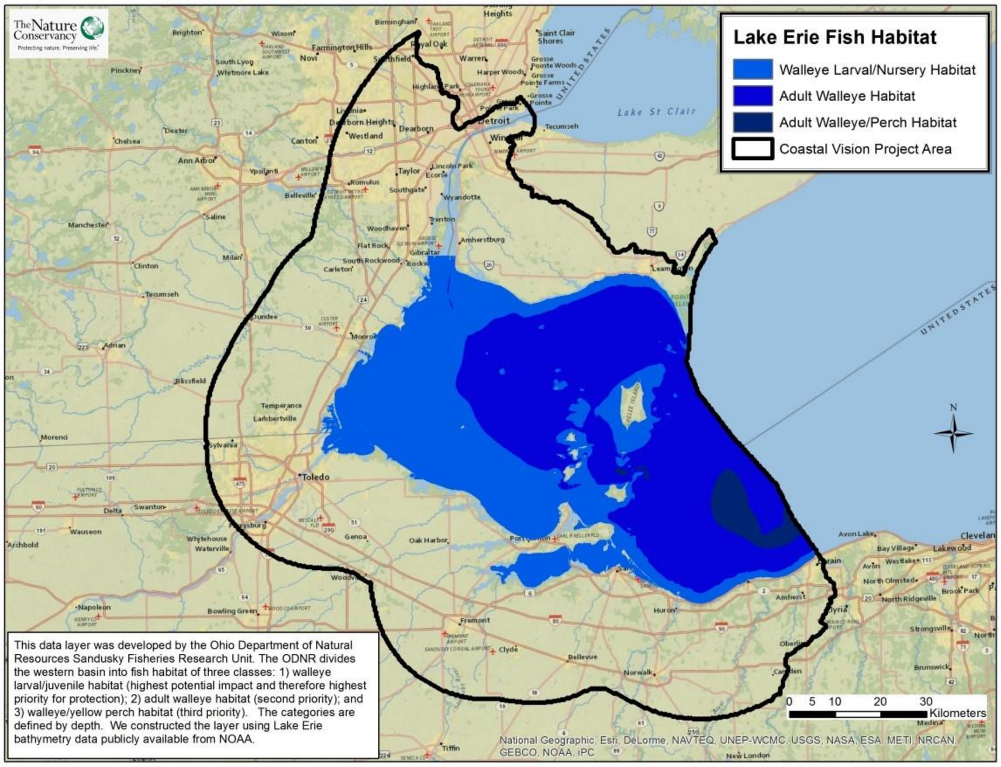

Nearshore Fish Habitat: *Fish habitats within the WLEB nearshore are depicted in the map above, with very dark blue areas indicating adult walleye and perch habitat, medium blue area showing adult walleye habitat, and light blue areas for nursery and walleye larval habitat. The study area is outlined in black.*

How is nearshore fish habitat in Lake Erie connected to regional ecological and social values?

This data layer uses walleye (*Sander vitreus*) and yellow perch (*Perca flavescens*), both native migratory fish, to provide a sense of priority for fish habitat in the Western Lake Erie Basin (WLEB). Walleye play a key role in the ecosystem as predators and facilitate the migration of other species like mussels, which ride in their gills during their larval stage[1]. This species, like other migratory fish, utilizes different parts of the lake at different life stages; as young fry walleye live in coastal wetlands or shallow, rocky reefs, but as adults they move to deeper parts of the lake[2]. Walleye, yellow perch and other sport fish species fuel an economy worth tens of millions in the WLEB; walleye and sauger are especially popular, luring 0.6 million of the 1.7 million anglers who contributed $1.9 billion to the WLEB economy in 2011[3]. Canadian commercial operators earned $7.8 million for the 4.8 million pounds of walleye caught in 2008 (out of a total catch of 30.1 million pounds)[4]. The Western Lake Erie Coastal Conservation Vision (WLECCV) Project recognizes that the range of habitat types available in the lake should be conserved in order to sustain adult fish populations, which contribute to the regional ecosystem and economy by adding biodiversity to the lake and supporting the recreational and commercial fishing industries.

Nearshore fish habitat data layer

The Lake Erie Biodiversity Conservation Strategy (LEBCS) established a goal of having at least two viable populations of each migratory fish species present in each basin of Lake Erie by 2030[5]. This data layer was developed following the methods set forth in a wind energy suitability analysis report by the Ohio Department of Natural Resources (ODNR) Sandusky Fisheries Research Unit, which recognizes that wind energy development in Lake Erie could negatively impact fish habitat. The ODNR divides the western basin into three fish classes: 1) walleye larva and juvenile habitat (highest potential impact and therefore highest priority for protection); 2) adult walleye habitat (second priority); and 3) walleye and yellow perch habitat (third priority). A fourth category (hypoxic zone) does not occur in the western basin. The categories are defined by depth: Category 1 includes all waters less than 8m depth; category 2 includes waters 8–13m in depth; and category 3 includes watersheds 13–18 m in depth. Based on these definitions, this data layer was constructed using Lake Erie bathymetry data and depicts the three different types of fish habitat.

Nearshore fish habitat data consisted of three depth based habitat classes: 1) walleye larval/juvenile habitat (highest potential impact and therefore highest priority for protection); 2) adult walleye habitat (second priority); and 3) walleye/yellow perch habitat (third priority). The amount of each habitat type was quantified for each planning unit. Full value (habitat area) was given to class 1, half value was given to class 2, and 1/3 value given to class 3.

Data sources and potential limitations

Bathymetry data for this layer were downloaded from the NOAA website. It would be ideal to include other migratory species in this analysis to ensure that the LEBCS goal for all migratory fish species is considered during the analysis; such data is not yet available for the WLEB, although a current mapping project underway at The Nature Conservancy may make it available in the future.

References and links

1. Pearsall, D., P. Carton de Grammont, C. Cavalieri , C. Chu, P. Doran, L. Elbing, D. Ewert, K. Hall, M. Herbert, M. Khoury, D. Kraus, S.Mysorekar, J. Paskus and A. Sasson. (2012). *Returning to a Healthy Lake: Lake Erie Biodiversity Conservation Strategy*. Technical Report. A joint publication of The Nature Conservancy, Nature Conservancy of Canada, and Michigan Natural Features Inventory. 340 pp. with Appendices. http://www.conservationgateway.org/ConservationByGeography/NorthAmerica/wholesystems/greatlakes/Pages/lakeerie.aspx ; http://www.fws.gov/midwest/endangered/clams/mussels.html
2. Pearsall, D., *et al.* 2012. *Returning to a Healthy Lake.*
3. 2011 National Survey of Fishing, Hunting, and Wildlife-Associated Recreation. August 2012. USFWS. www.doi.gov/news/pressreleases/upload/FWS-National-Preliminary-Report-2011.pdf
4. www.miseagrant.umich.edu/explore/about-the-great-lakes/lake-erie/
5. Pearsall, D., *et al.* 2012. *Returning to a Healthy Lake.*

Current and Restorable Coastal Wetlands

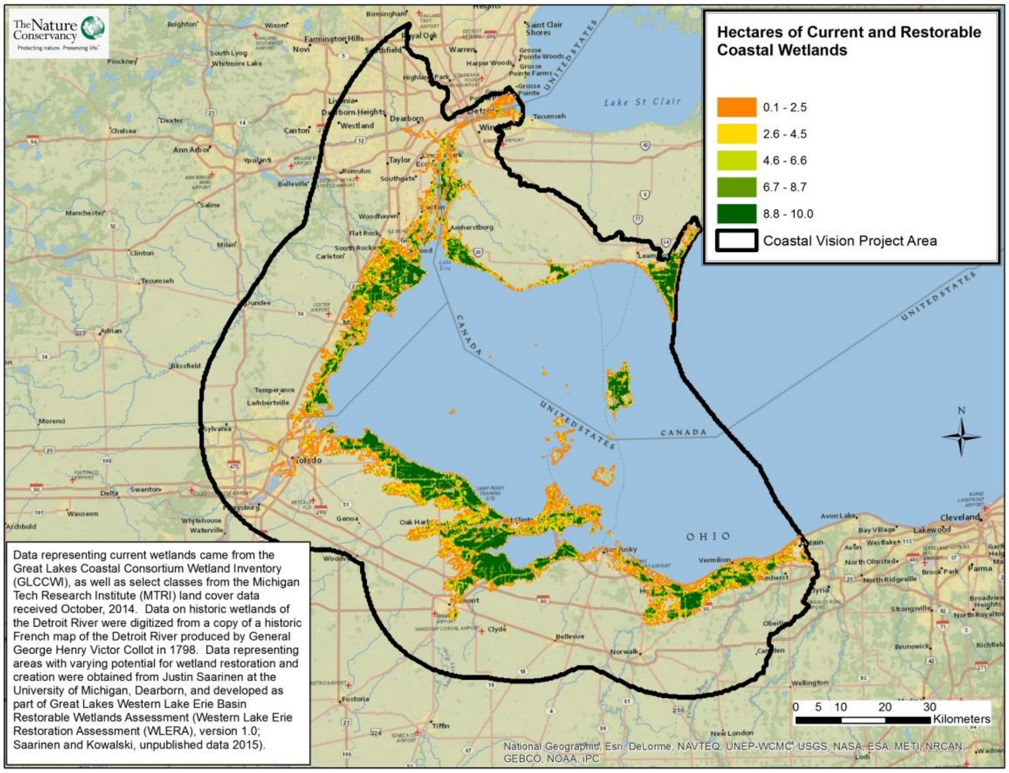

Current and Restorable Coastal Wetlands. *This map shows areas with existing wetlands and potential for wetland restoration. Areas with the potential to be restored to wetlands are displayed on a gradient from green to orange, with green indicating areas with the highest density of restorable land and orange indicating the lowest density of restorable land. The WLEB analysis area is outlined in black.*

Coastal wetlands in relation to regional ecological and social values

Coastal wetlands provide habitat for birds, spawning fish, and a diversity of amphibians, reptiles, insects, and plants. They are the most biodiverse habitats in Lake Erie, and provide critical stopover and breeding habitat for local migratory birds. Wetlands are also crucial spawning habitat for fish, and serve as buffers for coasts in the face of erosion-inducing storm surge. Finally, wetlands filter nutrients and sediment out of runoff that otherwise threatens Lake Erie water quality. These functions are essential for maintaining the nutrient and material cycles of Lake Erie and for maintaining the health of its biota[1]. Residents of the Western Lake Erie Basin depend on these wetland functions to improve water quality and help provide for recreation opportunities that support the regional economy. The robust fish and bird populations supported by coastal wetlands provide for a recreational fishing industry worth US$1.4 billion (2012), for commercial fisheries worth over $4.6 million (2012) on the U.S side and $33 million (2012) in Ontario[2], and for annual birdwatching revenues that are estimated at $26 million (2011)[3]. By filtering runoff, wetlands additionally contribute to the health of beach-goers and swimmers while simultaneously helping to maintain a supply of fresh drinking water to over 11 million people[4]. Coastal wetlands play a critical role in serving as buffers against storm waves, winds and flooding, all of which are increasing due to intensifying storm events. The Western Lake Erie Coastal Conservation Vision

Project recognizes the ecological and socioeconomic value of wetland habitats and uses this data layer to ensure that existing wetlands and areas with potential for wetland restoration are included in the analysis.

Current and restorable coastal wetlands data layer

The Lake Erie Biodiversity Conservation Strategy (LEBCS) established a 2030 goal of increasing coastal wetland area, as of 2011, by 10%[5]. The Western Lake Erie Coastal Conservation Vision analysis uses this data layer of current and potential coastal wetlands to determine optimal areas for restoration and creation of wetland habitat. This will aid in determining where to increase wetland habitat to achieve the LEBCS goal. The data layer shows current coastal wetlands and areas with some suitability for restoration to coastal wetland along the Western Lake Erie coast from the Detroit River in Michigan to Sandusky, Ohio. The current and restorable coastal wetlands data layer was created by combining data from four primary sources representing data on existing, former, or potential wetland areas.

Data sources and potential limitations

Data representing areas with varying potential for wetland restoration and creation were obtained from Justin Saarinen at the University of Michigan, Dearborn, and Kurt Kowalski of the US Geological Survey and developed as part of the Western Lake Erie Wetland Restoration Assessment (WLERA)[6]. The WLERA data did not exist for the Detroit River or anywhere in Ontario. For the Detroit River and Ontario, we compiled wetland polygons from The Great Lakes Coastal Wetland Consortium (GLCWC)[7] and select land cover classes (wetland classes, agriculture, and fallow field) from the Michigan Tech Research Institute (MTRI) coastal land cover dataset[8]; we restricted the data to elevations less than 176 meters because elevations above that have very low inundation frequencies and thus aren't suitable for restoration to coastal wetlands. In addition, data on historic wetlands of the Detroit River[9] were used to indicate suitable areas for wetland restoration after removing currently developed land.

The GLCWC data – as of this writing – must be requested from Don Uzarski, PI of the Great Lakes Coastal Wetland Monitoring Program. We used the "complete polygon coverage" file, as augmented by the Michigan Natural Features Inventory during the creation of the LEBCS. Products developed with this data should acknowledge the following groups: U.S. Geological Survey Water Resources Division, Environment Canada Canadian Wildlife Service-Ontario Region, Michigan Natural Features Inventory, and Ontario Ministry of Natural Resources.

References and links

1. Pearsall, D., et al. 2012. *Returning to a Healthy Lake: Lake Erie Biodiversity Conservation Strategy*. Technical Report. A joint publication of The Nature Conservancy, Nature Conservancy of Canada, and Michigan Natural Features Inventory. 340 pp. with Appendices. http://www.conservationgateway.org/ConservationByGeography/NorthAmerica/UnitedStates/michigan/Pages/lakeerie.aspx
2. http://www.lakeerieimprovement.org/wp-content/uploads/2012/02/leia-strategic-plan-final-12-17-2012.pdf

3. http://ohioseagrant.osu.edu/research/economic/?ID=R/ME-033#benefits
4. http://www.lakeerieimprovement.org/wp-content/uploads/2012/02/leia-strategic-plan-final-12-17-2012.pdf
5. Pearsall, D., et al. 2012. *Returning to a Healthy Lake: Lake Erie Biodiversity Conservation Strategy*. Technical Report. A joint publication of The Nature Conservancy, Nature Conservancy of Canada, and Michigan Natural Features Inventory. 340 pp. with Appendices. http://www.conservationgateway.org/ConservationByGeography/NorthAmerica/UnitedStates/michigan/Pages/lakeerie.aspx
6. Saarinen JA, Kowalski KP. 2015. Western Lake Erie Restoration Assessment (WLERA), version 1.0. Unpublished data.
7. Michigan Tech Research Institute (MTRI) land cover data received October, 2014 (http://geodjango.mtri.org/coastal-wetlands/). Citation: Bourgeau-Chavez, L.L., Endres, S.L., , Battaglia, M.J., Miller, M.E,Banda, E.C., Laubach, Z.M., Higman, P., Chow-Fraser, P. and Marcaccio, J. 2015. Development of a bi-national Great Lakes coastal wetland and land use map for resource management using multidate radar and optical satellite imagery.
8. Historic wetlands of the Detroit River were digitized from a copy of a historic French map of the Detroit River produced by General George Henry Victor Collot in 1798.

Coastal Terrestrial Biodiversity Significance

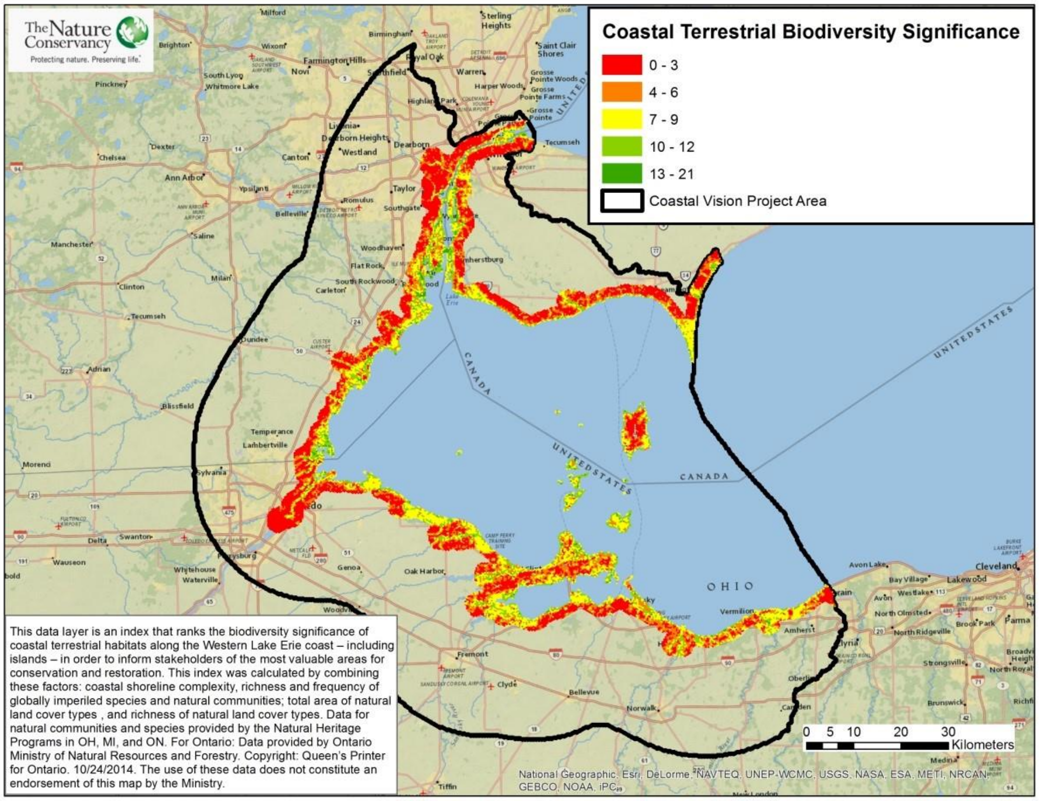

Coastal Terrestrial Biodiversity Significance: *This map shows the significance of coastal areas along Lake Erie for biodiversity conservation, with most significant areas in green and least significant areas in orange. The WLEB analysis area is outlined in black.*

Coastal terrestrial biodiversity in relation to regional ecological and social values

The coastal terrestrial system includes the area within roughly 2km of the Lake Erie shoreline; this area is critical habitat for migratory shorebirds, rare plants, reptiles and amphibians, including the eastern white-fringed orchid (*Platanthera leucophaea*), eastern fox snake (*Pantherophis gloydi*), and elusive clubtail (*Stylurus notatus*). This system is composed of small and isolated communities of forested wetland, emergent marsh, beach-dune systems, oak savanna communities, alvar (limestone bedrock) communities and upland forest. The beaches and aesthetic value of the coastal terrestrial system make it important for residential, industrial, and recreational uses. Swimmers frequent its beaches, and its aesthetics add value to coastal property and to tourism along the coast. However, development, shoreline hardening, and other human activities threaten the species and communities of this system with habitat loss and fragmentation. The WLECCV seeks to identify opportunities for conservation to benefit both the natural coastal habitat and the people that depend on it.

Coastal terrestrial biodiversity significance data layer

The Lake Erie Biodiversity Conservation Strategy (LEBCS) established a goal of having 40 percent of the coastal terrestrial system in natural landcover by 2030[1]. This data layer is an index that ranks the biodiversity significance of coastal terrestrial habitats along the Western Lake Erie coast – including islands – in order to inform stakeholders of the most valuable areas for conservation and restoration. Similar to an index used in the LEBCS, this index was calculated by combining these seven factors: coastal shoreline complexity, richness of globally imperiled species and natural communities[1], frequency of globally imperiled species and natural communities, total area of natural land cover types[2], and richness of these natural land cover types.

Data sources and potential limitations

Natural land cover data for the coastal terrestrial system in the U.S. and Canada were obtained from the Great Lakes Coastal Wetland Mapping project. Data for globally imperiled species and natural communities were obtained from the Natural Heritage programs in OH, MI, and Ontario (for Ontario, data were provided by Ontario Ministry of Natural Resources and Forestry; copyright: Queen's Printer for Ontario. 10/24/2014. The use of these data does not constitute an endorsement of this product by the Ministry). Shoreline classes were obtained from the National Oceanic and Atmospheric Administration.

References and links

1. Pearsall, D., P. Carton de Grammont, C. Cavalieri , C. Chu, P. Doran, L. Elbing, D. Ewert, K. Hall, M. Herbert, M. Khoury, D. Kraus, S. Mysorekar, J. Paskus and A. Sasson. (2012). *Returning to a Healthy Lake: Lake Erie Biodiversity Conservation Strategy*. Technical Report. A joint publication of The Nature Conservancy, Nature Conservancy of Canada, and Michigan Natural Features Inventory. 340 pp. with appendices. http://www.conservationgateway.org/ConservationByGeography/NorthAmerica/wholesystems/greatlakes/Pages/lake erie.aspx
2. Michigan Tech Research Institute. 2014. Great Lakes Coastal Mapping Project. Land cover data obtained for Lake Erie on October 13, 2014.
3. National Oceanic and Atmospheric Administration, Medium Resolution Shoreline dataset: http://www.glerl.noaa.gov/data/char/glshoreline.html

---

[1] Based on Element Occurrence records obtained from Natural Heritage programs in Ontario, Michigan, and Ohio with Global Imperilment Ranks of G1-G3 and had been observed more recently than 1982.

[2] Forest, Shrub, Water, Aquatic Bed, Wetland, Schoenoplectus, Typha, Phragmites, Wetland Shrub, Forested Wetland, using the Great Lakes Coastal Wetland Mapping data (Michigan Tech Research Institute 2014)

Migratory Bird Stopover Habitat

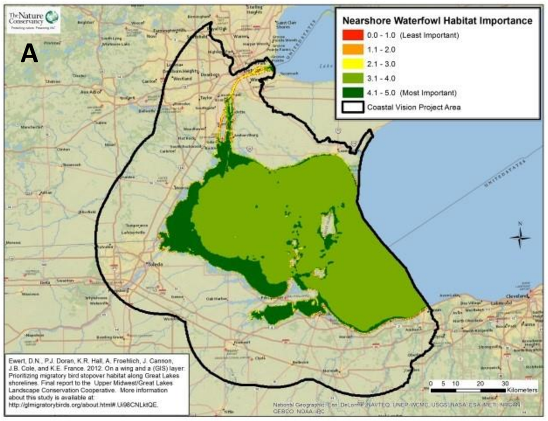

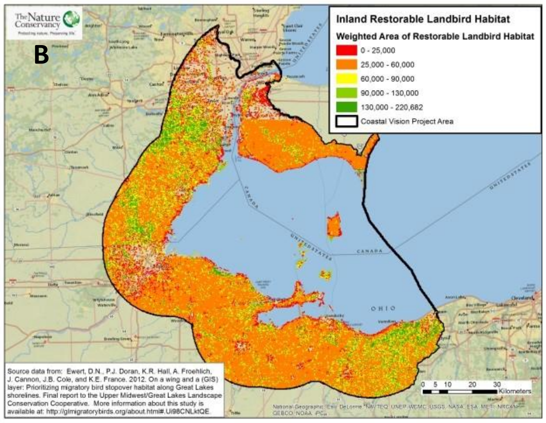

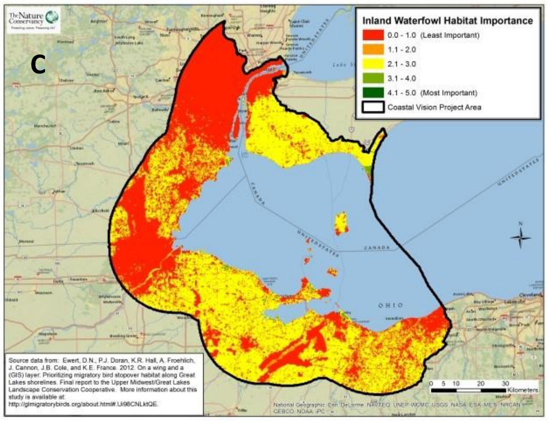

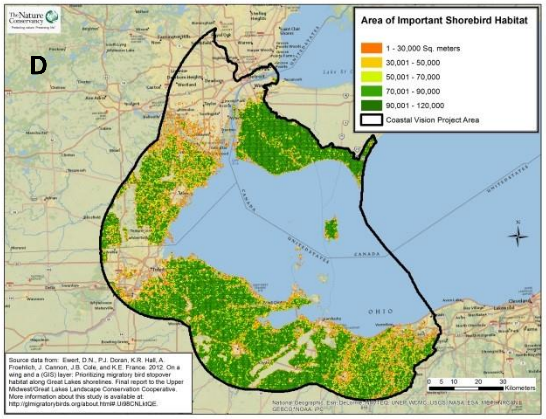

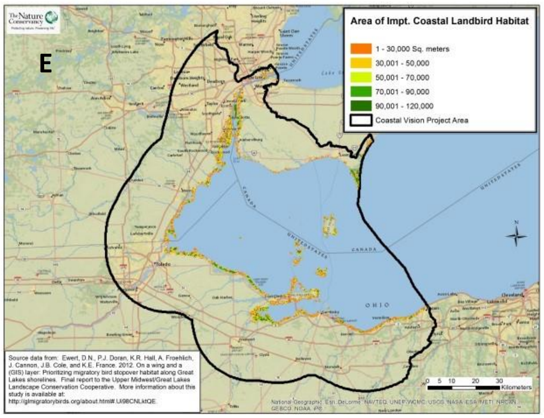

***Migratory Bird Stopover Habitat**. The study area is outlined in black. The suitability of land cover for bird habitat is shown on a gradient from red (least suitable) to green (most suitable). Five types of stopover habitat are depicted: A) Nearshore Waterfowl Habitat, B) Inland Restorable Landbird Habitat, C) Inland Waterfowl Habitat, D) Shorebird Habitat, and E) Coastal Landbird Habitat.*

Migratory birds in relation to regional ecological and social values

Located at the intersection of the Mississippi and North Atlantic Flyways, the Western Lake Erie Basin (WLEB) is an important corridor for northern birds migrating to and from the southern United States to Argentina. Songbirds, waterfowl, shorebirds, hawks, owls, and other species that travel through this region are dependent on food and shelter offered by the Western Lake Erie shoreline and inland stopover habitat during the high-stress periods of spring and fall migration. In turn, the huge numbers

of migrating birds represent an important component of the food chain by eating millions of insects and fruits and dispersing seeds along their route. The preservation of stopover sites ensures the survival of these birds, which is critical for both the biodiversity of the lake basin and the continued presence of these species in their southern range. This makes conservation efforts in the Lake Erie basin both locally and internationally important. In addition to their ecological role, migratory birds are treasured by the many bird enthusiasts who visit or live in the region. The seasonal diversity and abundance of migrating birds attract birders from around the world, who collectively benefit the region's economy by spending an estimated $26 million on food, gas, lodging, and other expenses related to their visit to the Lake Erie coast[1]. The WLECCV project acknowledges migratory birds as an important target for conserving WLEB biodiversity and the tourism industry that depends on these species.

Migratory Bird Stopover Habitat data layers

The Lake Erie Biodiversity Conservation Strategy (LEBCS) set the following 2030 goals for the WLEB: 1) At least 30% of the 2 km coastal area comprises high quality stopover habitat for migrating landbirds; 2) at least 10% of coastal area comprises high quality stopover habitat for migrating shorebirds; 3) at least 50% of the 2 km coastal area, including coastal wetlands, comprises high quality stopover habitat for migrating waterfowl; 4) at least 80% of the 2 km coastal area that is high quality stopover habitat for all bird groups is in conservation ownership or management[2]. The WLECCV project incorporated the habitat scores from a recent study by Ewert et al. (2012) for each of the three bird groups and separated them into five categories: coastal landbird habitat (0-1.6 km from shore), inland restorable landbird habitat (1.6 -25 km from shore), shorebird habitat (≤25 km of shore), nearshore waterfowl habitat (from Lake Erie shore to 15 m depth), and inland waterfowl habitat (islands and up to 25 km inland of Lake Erie shore). These data layers facilitate achievement of LEBCS goals by depicting existing stopover habitats for shorebirds, waterfowl, and landbirds (coastal only), and restorable inland habitat for landbirds, thus indicating where conservation efforts best accomplish these goals. Ewert et al (2012) identified and scored attributes of stopover sites and potential stopover habitat, such as land cover data and proximity to water bodies, within 25km of the Lake Michigan, Huron, Erie, and Ontario shorelines, and their connecting water bodies[3]. The criteria used in the study to predict the locations of stopover sites are based on a literature review and on unpublished information from reports and regional stopover experts; further study method details are available online.

The inland restorable landbird stopover habitat layer incorporates current stopover habitat (Ewert et al. 2012) and restorable habitat in two categories: highly restorable and moderately restorable habitat. These additional areas were identified by recoding landcover data (Provincial Land Cover for Ontario, and CCAP for the US). For example, pasture and hayfields were placed in the highly restorable category, and low intensity developed land was placed in the moderately restorable category. Each of these categories was assigned a value (1 for current habitat; 0.5 for highly restorable habitat; 0.25 for moderately restorable habitat) and the total area of each category was multiplied by these weighting values to calculate a final value for each 10-ha hexagon.

Data sources and potential limitations

The Nature Conservancy is developing a web portal that will allow the public to access stopover maps and report information. The primary land cover data sets used for this layer were published in 2006 (US) and 1999 (Canada). Consequently, changes in land use since that time have not been captured in the analysis; this is an inevitable limitation of data availability at the time of the project.

References and links

1. http://ohioseagrant.osu.edu/research/economic/?ID=R/ME-033#benefits
2. Pearsall, D., P. Carton de Grammont, C. Cavalieri , C. Chu, P. Doran, L. Elbing, D. Ewert, K. Hall, M. Herbert, M. Khoury, D. Kraus, S. Mysorekar, J. Paskus and A. Sasson. (2012). *Returning to a Healthy Lake: Lake Erie Biodiversity Conservation Strategy*. Technical Report. A joint publication of The Nature Conservancy, Nature Conservancy of Canada, and Michigan Natural Features Inventory. 340 pp. with Appendices. http://www.conservationgateway.org/ConservationByGeography/NorthAmerica/wholesystems/greatlakes/Pages/lakeerie.aspx .
3. Ewert, D.N., P.J. Doran, K.R. Hall, A. Froehlich, J. Cannon, J.B. Cole, and K.E. France. (2012). On a wing and a (GIS) layer: Prioritizing migratory bird stopover habitat along Great Lakes shorelines. Final report to the Upper Midwest/Great Lakes Landscape Conservation Cooperative.

Human Well-being Targets:

Beaches

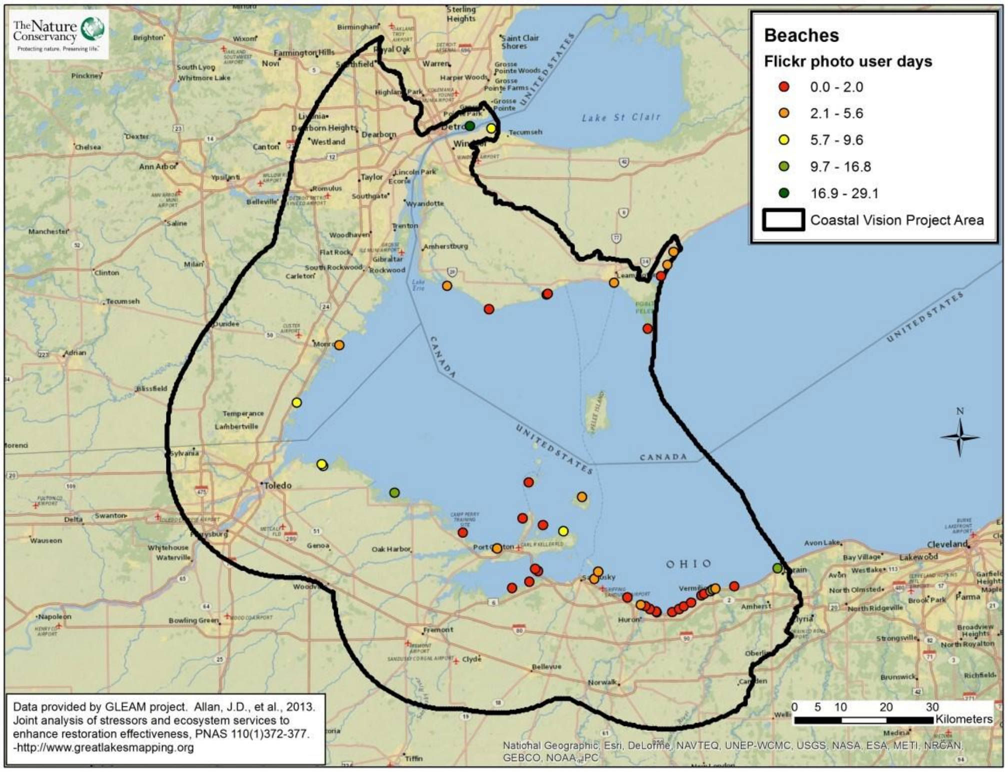

Beaches. *Publically accessible beaches are depicted by colored dots. Colors represent the number of flickr photo-user days, which is a proxy for the number of visitors to the beach, with green as the highest density and red as the lowest. The WLEB analysis area is outlined in black.*

Public beaches in relation to regional ecological and social values

Beaches provide the public with an opportunity to connect with nature and enjoy activities like swimming, walking and picnicking. Those who visit the beach also tend to visit nearby restaurants, shops and hotels. In this way, beaches also provide indirect benefits to the local coastal economy, in addition to directly benefiting their visitors. A survey of Maumee Bay visitors conducted in 1999 found that visitors spent an average of US$25 per day; given that over a million people visit each year, this beach is estimated to bring in about US$6 million annually[1]. A 2010 study in Michigan found that closing a single Michigan beach for even one day could result in a loss ranging from US$130,000 to US$24 million[2]; a 2001 study estimated that reducing one water quality advisory to all Ohio beaches would provide an aggregate benefit of US$3.2-3.4 million[3]. There are surprisingly few recent studies on the value of Great Lakes beaches, but the 2010 study of Michigan beaches mentioned above provides a calculated US$56 average per trip value to Lake Erie beaches, which is slightly higher than estimates for beaches on the other three Great Lakes surrounding Michigan[4]. This suggests that Lake Erie beaches are not only still valuable to the local economy, but may actually be worth more than earlier studies, like that of Maumee Bay, have estimated. A survey of 71 stakeholders conducted in 2012 for the Lake Erie Biodiversity

Conservation Strategy (LEBCS) found that recreation and tourism was regarded as the most highly valued service provided by Lake Erie and its coastal area[5]. The Public Beaches data layer is included in the Western Lake Erie Coastal Conservation Vision (WLECCV) analysis in recognition of the service that beaches provide to people and to the regional coastal economy.

Public Beaches data layer

This layer was developed by the Great Lakes Environmental Assessment and Mapping Project (GLEAM)[6]. The GLEAM project team identified beaches in the Western Lake Erie Basin (WLEB) and attributed them with Flickr photo activity as a correlate of potential visitation levels. American beach data was obtained from the U.S. EPA Beaches Environmental Assessment and Coastal Health Act Geospatial database, while Canadian beach data was obtained from Environment Canada; a few additional beaches were identified from the protected lands databases for the U.S. and Canada. The GLEAM project team calculated photo-user days using the Natural Capital Project's InVEST model (recreation initialization). This model is described in Wood et al (2013)[7]. The GLEAM team used a 500 meter buffer around each beach to capture Flickr activity along the shoreline. The result represents the number of annual photo user days, defined as the number of days a unique Flickr user uploads at least one photograph within that 500-meter buffer. The total is an average from 2005-2012. Higher photo-user days suggest that these beaches are frequented by the highest number of people. Overall, there are 47 beaches on the WLEB coast including the Detroit River.

Data sources & potential limitations

Data were obtained through personal communication with the GLEAM team.

References & Links

1. Sohngen, B., F. Lichtkppler, and M. Bielen. 1999. *The value of day trips to Lake Erie beaches*. Ohio Sea Grant College Program.
2. Song, F., F. Lupi, and M. Kaplowitz. 2010. "Valuing Great Lakes Beaches." *Agricultural & Applied Economics Association*: 25-27.
3. Murray, Chris, Brent Sohngen, and Linwood Pendleton. "Valuing water quality advisories and beach amenities in the Great Lakes." *Water Resources Research* 37.10 (2001): 2583-2590.
4. Song, F., F. Lupi, and M. Kaplowitz. 2010. "Valuing Great Lakes Beaches." *Agricultural & Applied Economics Association*: 25-27.
5. Pearsall, D., *et al.*. 2012. "Returning to a Healthy Lake: Lake Erie Biodiversity Conservation Strategy." Technical Report. A joint publication of The Nature Conservancy, Nature Conservancy of Canada, and Michigan Natural Features Inventory. 340 pp. with Appendices. http://www.conservationgateway.org/ConservationByGeography/NorthAmerica/wholesystems/greatlakes/Pages/lakeerie.aspx
6. Allan, J.D., et al., 2013. Joint analysis of stressors and ecosystem services to enhance restoration effectiveness, PNAS 110(1)372-377. http://www.greatlakesmapping.org .
7. Wood, S.A., Guerry, A.D., Silver, J.M. & Lacayo, M. 2013. Using social media to quantify nature-based tourism and recreation. Sci. Rep. 3, 2976; DOI:10.1038/srep02976.

Birding Visits

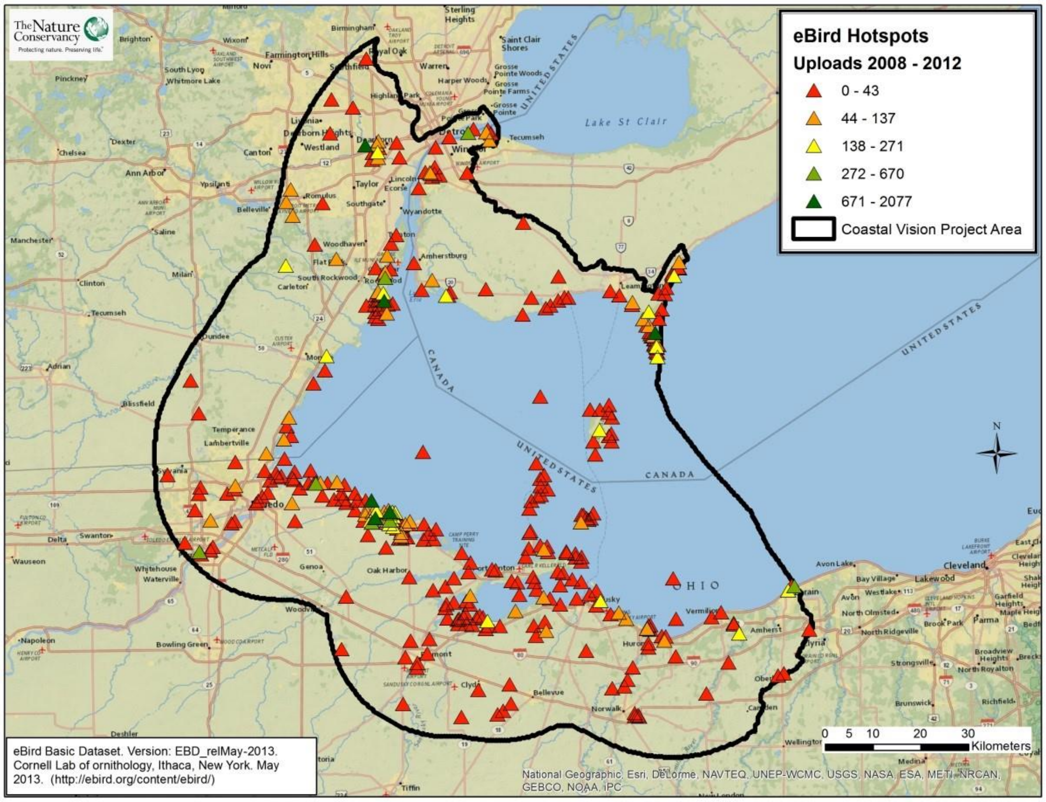

Birding Visits. *Popular birding hotspots are shown as triangles, with dark green indicating the most visited areas and red indicating the least visited. The WLEB analysis area is outlined in black.*

Birding in relation to regional ecological and social values

Birding, a popular activity among locals and tourists, links the Western Lake Erie economy to the ecology of the region. In 2012, 71 stakeholders in the Western Lake Erie Basin (WLEB) were surveyed as part of the Lake Erie Biodiversity Conservation Strategy (LEBCS). Results showed that those stakeholders were found to value nature-based recreation, such as birding, as the number one ecosystem service provided by Lake Erie and its coasts. Millions of migratory birds use the Lake Erie coast as stopover habitat, playing an ecologically important role as insectivores and seed dispersers while simultaneously impressing birders with their diversity and large numbers. The Lake Erie coast has consequently become the most popular Ohio birding destination visited by resident and out-of-state birders. In 2006, approximately 120,000 visitors came to Ohio to bird watch[1]. Popular birding sites in the region include the Lake Erie Birding Trail in Ohio, Lake Erie Metropark in Michigan, and Point Pelee National Park in Ontario. Six popular Lake Erie birding destinations in Ohio alone were found to bring in approximately $26 million[2] for the region's tourism economy, which suggests a potential to grow the tourism sector of the economy of this basin. Birding and recreational activities feed into an $11.5 billion coastal tourism industry in Ohio's seven coastal counties[3] and a $12.7 billion leisure tourism industry in Michigan. This economy relies on the continued health of resident and migratory birds and the habitats they depend

on, which closely ties human concerns to the biological conservation of these areas. To represent the level of intensity, and thus the potential importance, of bird watching in different places within the WLEB, we used data collected from eBird, a citizen science program run out of the Cornell Lab of Ornithology.

Birding Visits data layer

We used eBird data to represent birding activity in the WLEB. This data layer shows birding "hotspots", as recorded by bird watchers, and was used to include this valued ecosystem service in the Western Lake Erie Coastal Conservation Vision analysis. The Cornell Lab of Ornithology launched eBird as a citizen science program for the public to keep an online checklist, available for use with smartphone application or the internet. Recreational and professional bird watchers record the method, location, and time of their birding trip, and then list the species heard/observed at that location. An eBird committee can designate public locations as "hotspots," which are defined as "good birding sites that are accessible and likely to be birded by multiple people." Users can then note that their observations were taken at these hotspots. This program was launched in 2002 and has become very popular. In August 2013, 428,043 observations of 260 bird species were made in Ohio, and 528,865 observations of 302 species were made in Michigan[3]

Data quality is checked by regional and local experts, which includes the approval of hotspots, and data is then made publicly available. The WLECCV analysis includes the most recent five complete years (2008 - 2012) of data recording the total number of visits at each hotspot; this data was selected from a downloaded copy of the May 2013 version of eBird data. The total number of recorded visits to each hotspot used in the analysis is proportional to the number of individuals who visited each site.

Data sources & potential limitations

Data for the eBird data layer was downloaded from Cornell University's eBird website; data from 2008-2012 were taken from the May 2013 version of the eBird hotspot data for our analysis. The website's data is continually updated with public use, which makes it possible to update this layer over time.

References & Links

1. Worrell, Chris M. "Areas east of Cleveland heavily represented along new Lake Erie Birding Trail." Available at: http://www.cleveland.com/euclid/index.ssf/2011/10/areas_east_of_cleveland_heavil.html
2. http://ohioseagrant.osu.edu/research/economic/?ID=R/ME-033#benefits
3. http://www.lakeerieimprovement.org/wp-content/uploads/2012/02/leia-strategic-plan-final-12-17-2012.pdf
4. http://tourismplan.anr.msu.edu/docs/Michigan_Tourism.pdf
5. http://ebird.org/ebird/eBirdReports

Commercial Fishing

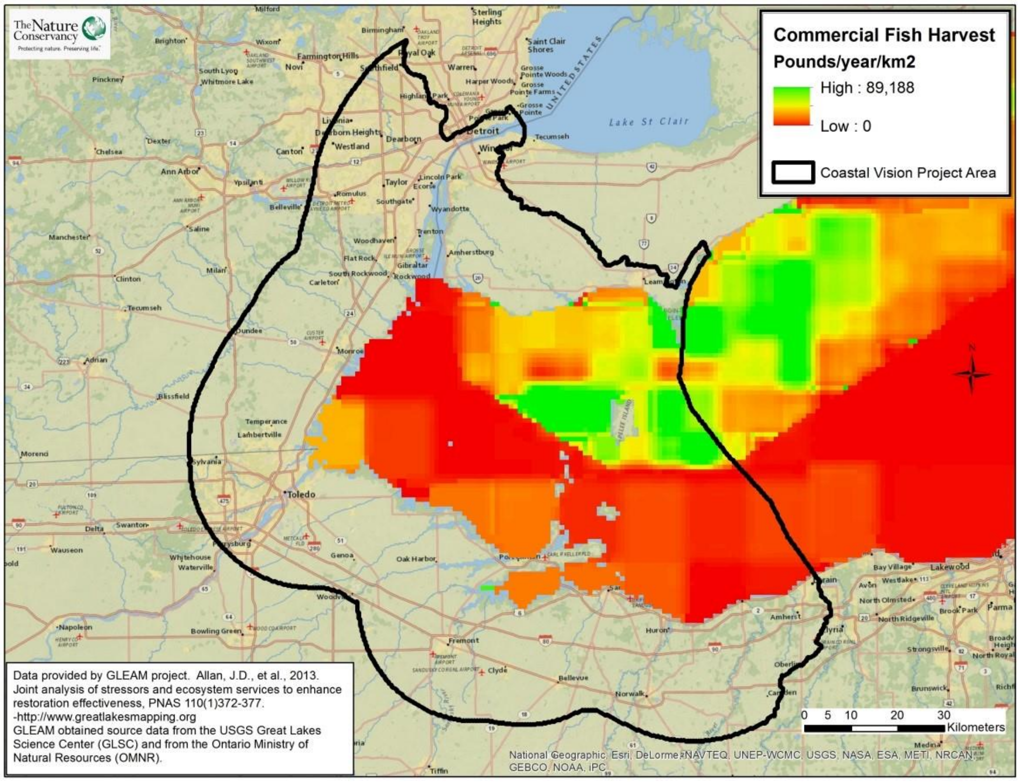

Commercial Fishing. *The density of commercial fishing harvest is shown on a gradient from green to red, with green representing the greatest catch size per km². The WLEB analysis area is outlined in black.*

Commercial fishing in relation to regional ecological and social values

The Great Lakes provide commercial fishermen with an annual average harvest of nearly 50 million pounds[1]. Lake Erie supports the largest commercial fishery, which consists primarily of walleye (*Sander vitreus*) and yellow perch (*Perca flavescens*)[2]. Fishermen from Ohio alone harvested 4.8 million pounds of fish—primarily yellow perch, white bass, white perch, and lake whitefish—worth an estimated US$5.8 million, from Lake Erie in 2012; more than one third of this catch (38.5%) was obtained from the Western Basin[3]. In 2012, Michigan commercial fishermen caught approximately 1.4 million pounds of fish—primarily carp (Cyprinidae), bigmouth buffalo (*Ictiobus cyprinellus*), sheephead (*Aplodinotus grunniens*), channel catfish (*Ictalurus punctatus*), and gizzard shad (*Dorsoma cepadianum*)—in Lake Erie, worth US$553,155[4]. The Ontario commercial catch in 2011 was about 26.5 million pounds, worth more than CA$33 million. In general, 80% of the value of Ontario's commercial fishery is harvested from Lake Erie, where the catch consists mostly of walleye and yellow perch[5]. Although the scale of the fishery in Lake Erie is impressive, it is only three-quarters of its historic size[6]. In the face of pressure from over-fishing, pollution, habitat destruction, and exotic species, it is essential that we effectively manage the health and subsequent yield of remaining fish populations. This layer is included to ensure that the needs of commercial fishing interests are considered in the analysis.

Commercial Fishing data layer

These data were compiled by the Great Lakes Environmental Assessment and Mapping Project (GLEAM) team using data obtained from the USGS Great Lakes Science Center (GLSC) and the Ontario Ministry of Natural Resources (OMNR). It shows the commercial catch by 10-minute grid cell for the U.S. side of Lake Erie and 5-minute grid cell for the Canadian side. The data for Canada represent round pounds[7] per year per km^2 for 2000 through 2009; for the US, the data represent round pounds per year per km^2 over the years 1998 through 2007[8]. The GLEAM project down-scaled the data to 1-km pixels using the cubic convolution algorithm in ArcGIS and assuming equal distribution of harvest within each reporting unit. We attributed our 10-ha planning units based on the harvest value at the center point of each hexagon. The resulting map above shows that the highest density of commercial fishing occurs on the Canadian side of the lake, as expected given the regulations on U.S. fishing.

Data sources & potential limitations

Data were obtained through personal communication with GLEAM project investigators. There are no data available for the Detroit River.

References & Links

1. http://greatlakesmapping.org/great_lake_stressors/3/commercial-fishing
2. http://greatlakesmapping.org/great_lake_stressors/3/commercial-fishing ; https://www.walleyecentral.com/articles/?a=1370 3. Ohio Division of Wildlife (ODW). 2013. Ohio's Lake Erie Fisheries, 2012. Annual status report. Federal Aid in Fish Restoration Project F69-P. Ohio Department of Natural Resources, Division of Wildlife, Lake Erie Fisheries Units, Fairport and Sandusky. http://www.dnr.state.oh.us/Portals/9/pdf/eStatus2012.pdf
4. Michigan Department of Natural Resources (DNR). 2012. State-Licensed Commercial Fishing Data for Michigan. http://www.michigan.gov/documents/dnr/2012_Commercial_Fishing_Data_for_Michigan_414185_7.pdf
5. http://www.mnr.gov.on.ca/en/Business/GreatLakes/2ColumnSubPage/STEL02_173913.html#Commercial_Fisheries
6. http://www.epa.gov/glnpo/atlas/glat-ch3.html#Commercial Fisheries 7. Round pounds refers to the weight of a catch before processing of fish.
8. http://www.greatlakesmapping.org/great_lake_stressors/3/commercial-fishing

Surface Water Intakes

Due to the sensitivity of this data no map is presented.

Surface water intakes in relation to regional ecological and social values

People have been withdrawing water from Lake Erie for over 150 years. Millions of Western Lake Erie Basin (WLEB) residents, along with industry, rely on viable infrastructure to access potable water from Lake Erie. In 2011, Lake Erie surface water withdrawals totaled 59 billion gallons per day[1]. The provision of water and water purification are two of the top 10 most highly valued ecosystem services provided by Lake Erie and its coastal area, according to a survey of lake managers and conservation practitioners conducted as part of the Lake Erie Biodiversity Conservation Strategy (LEBCS)[2]. We also recognize that these same coastal surface waters provide spiritual and cultural fulfillment for native peoples[3]. The quality of these valued services is currently threatened by pollution. In 2013, high levels of the toxin microcystin forced a small municipal water intake facility in Carroll Township, Ohio, to close, affecting roughly 2,000 customers[4]. In the same year, the elevated concentration of neurotoxins in the lake forced the city of Toledo to request an additional $1 million (USD) for chemicals to ensure proper treatment of the city's drinking water[5]. Then, in 2014, microcystin levels exceeded standards for the City of Toledo, which prompted a "do not drink" order forcing roughly 500,000 people to find alternative water sources for two days[3]. Surface water intakes not only give a supply of drinking water to local citizens, but also provide water for other activities, including industrial applications, power generation facilities, and irrigation for agriculture. Protection of water intake locations, and of the water source, can alleviate the negative ecological and economic impacts of pollution by preventing contamination from entering the lake. This is done by preserving natural land cover around intake points, which helps to filter water before it enters intakes, thereby reducing treatment costs. Protecting surface water intake points is a prime example of a conservation strategy that recognizes the intertwined relationship of social values, economic interests, and ecological concerns.

Surface water intake data layer

This layer includes locations of water intake points on land and in Lake Erie or the Detroit River. For our analysis, we buffered each point by 1 km to represent critical response areas and protected zones, which differ slightly in extent among the three jurisdictions (OH, MI, and ON). Some points in the layer also reflect locations of water treatment facilities. Due to the sensitivity of the water intake points to potential harm, we have agreed not to display maps of the water intake points. To prepare to populate hexes with these points, the Western Lake Erie Coastal Conservation Vision project team created 1km buffers around the U.S. intake points, with the exception of points in the Detroit River, which were only buffered on the upstream side. Canadian intake points already had buffers and these varied slightly depending on where they were. The team then computed the area of these buffers in each hexagon using an intersect process in GIS. When overlapping buffers existed the area was counted each time to emphasize these important areas.

Data sources & potential limitations

---

[3] http://www.toledoblade.com/watercrisis

Data for Ohio were distributed by the OH Department of Natural Resources (February 2014). The Essex Region Conservation Authority provided data for Ontario (February 2014). Michigan intake points were distributed by the MI Department of Environmental Quality (February 2014; with permission from the Detroit Water and Sewage Department, the City of Monroe Water Plant, and the Water Utility Director for Frenchtown Township).

References & Links

1. Great Lakes Commission (2013) Annual Report of the Great Lakes Regional Water Use Database. No. 21, May. Retrieved January 21, 2014 from www.projects.glc.org
2. 71 lake managers and conservation practitioners, representing a X percent response to the LEBCS survey.
3. Mother Earth Water Walker, accessed 4 November 2014: http://www.motherearthwaterwalk.com/?page_id=11
4. Henry, T. (2013, September 15) Carroll Township's scare with toxin a 'wake-up call.' *The Toledo Blade*. Retrieved January 21, 2014, from www.toldeoblade.com.
5. Troy, T. and Henry, T. (2013, September 24) City water plant seeks extra $1M for algae. *The Toledo Blade*. Retrieved January 21, 2014, from www.toldeoblade.com.

Hunting Areas

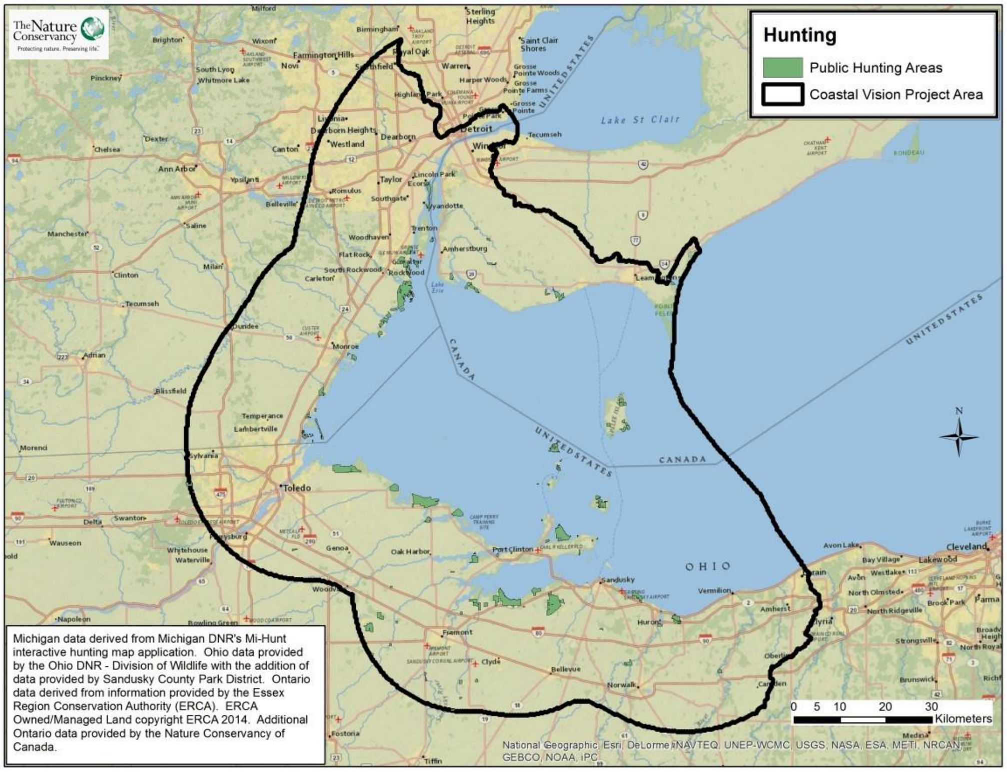

Hunting Areas. *Public lands open to hunting are shown in green. The WLEB study area is outlined in black.*

Hunting in relation to social and ecological values

In Michigan, Ohio, and Ontario combined, there were an estimated 1.97 million hunters who took part in one of the 2011 hunting seasons[1]. In the same year, an estimated $4.65 billion were spent on hunting related activities across the region[2]. The WLEB is home to rich hunting grounds for white-tailed deer, small game and waterfowl[3]. This was not always the case however, as wildlife populations were drastically altered in the early 1900's by habitat conversion and overhunting. In response, Ohio restricted all deer hunting until 1943, when rebounding deer populations allowed for the state's first modern day gun-season in three counties[4]. In 1956, the state opened all 88 counties to hunting, with a total of 3,911 deer harvested, which pales in comparison to the state's harvest of 218,910 in 2012-13[5]. Hunting is an important wildlife management technique, allowing state officials to regulate the number and type of prey hunted to prevent species from dominating, which can decrease biodiversity. The WLEB also has excellent waterfowl hunting opportunities due to its location on the intersection of the Mississippi and Atlantic Flyways. Turkey and small game hunting are also very popular with sportsmen across the region. Michigan, Ohio and Ontario have all designated state-owned property for hunters to utilize during open hunting seasons. The hunting data layer was included in this analysis to identify areas important to hunters within the WLEB.

Hunting Areas data layer

The WLECCV team compiled this data layer from a number of state- and provincial-level sources. Only those public lands which are explicitly listed as being open to hunting are included in this layer. In total, 29,184 acres of public hunting lands in 142 parcels were identified and included in the Marxan analysis.

Data sources and potential limitations

Public hunting areas in Michigan were derived from Michigan DNR's Mi-Hunt interactive hunting map application and attributed to state and federal land polygons in the CARL lands layer (June 2007; accessed February 2008). Hunting areas in Ohio were identified using information provided by the Ohio DNR's "Ohio Wildlife Areas" website and then attributed to state and federal land polygons contained in the same CARL lands layer. Several properties were digitized using information on the Sandusky County Park District's website. Canadian hunting areas were select by identifying areas within the Canadian parks and recreation dataset where hunting is allowed; this dataset was obtained through Nature Conservancy of Canada (personal communication; see Parks & Recreation factsheet for dates and details) and the Essex Region Conservation Authority, which provided data specifically for conservation lands (received March 2014). Ontario's public hunting areas were identified from information provided by the Essex Region Conservation Authority (ERCA; personal communication)[6]. Some minor verification was required to ensure that the data layer reflected the proper parcels. Additional data for Ontario was provided by the Nature Conservancy of Canada (received November 2014).

References and links

1. http://www.michigan.gov/dnr/0,4570,7-153-10366-121641--,00.html (MI data); http://www2.ohiodnr.gov/news/post/late-season-deerhunting-opportunities-available-in-ohio (OH data); http://www.mnr.gov.on.ca/stdprodconsume/groups/lr/@mnr/@fw/documents/report/stdprod_109164.pdf (ON data)
2. http://www.nssf.org/PDF/research/HuntingInAmerica_EconomicForceForConservation.pdf (MI and OH data), http://www.ofah.org/hunting/we-are-hunters-economy (ON data)
3. http://www.qdma.com/uploads/pdf/WR2012.pdf
4. http://www2.ohiodnr.gov/news/post/ohio-hunters-harvest-more-than-75-000-deer-during-weeklong-gun-season
5. http://www.dnr.state.oh.us/Portals/9/pdf/pub304.pdf
6. Information from ERCA is ERCA Owned/Managed Land copyright ERCA 2014. The location and characterization of these sites as shown are approximate and are subject to change. Copyright ERCA 2012.

Parks and Recreation Lands

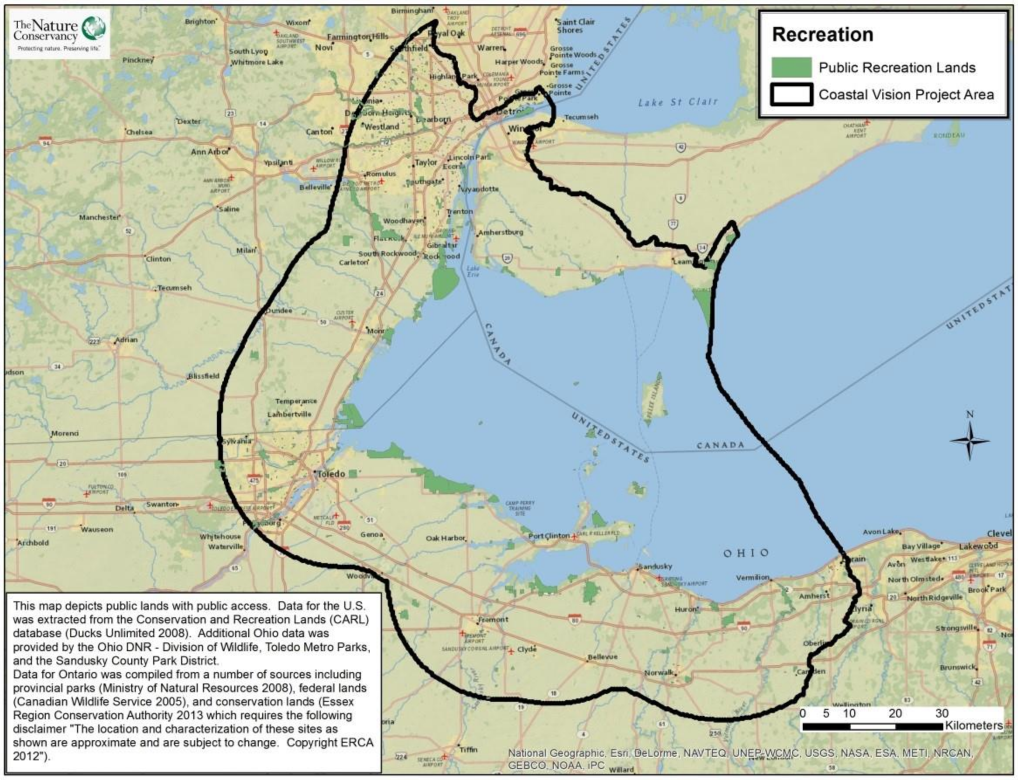

Parks and Recreation Lands. *Federal, provincial, state, county, and local parks are shown in green. The WLEB study area is outlined in black.*

Parks and recreation lands in relation to regional ecological and social values

Parks and recreational lands serve a variety of purposes, ranging from creating opportunities for recreation, to celebrating historical places, to conserving nature for citizens' enjoyment and education. Many of these areas contribute to conserving Western Lake Erie coastal areas and provide residents with access to nature, which is a critical benefit in urban areas where the majority of natural land cover has been converted to other uses. A variety of studies relay ways in which people are increasingly disconnected from nature, corresponding to an increase in health problems like obesity and to decreasing happiness and social cohesion among members of society[1]. The availability of parks and access to nature is therefore important for the physical and mental health of residents of the Western Lake Erie Basin (WLEB). A survey of 71 local natural resource professionals and conservation practitioners conducted for the Lake Erie Biodiversity Conservation Strategy (LEBCS) revealed that the most important ecosystem service provided by Lake Erie and its coastal area is recreation and tourism[2]. Ohio state parks like East Harbor, Maumee Bay, Marblehead Lighthouse, and several island parks recorded 0.9-1.5 million visitors per park in 2011 alone, indicating the importance of these places to the region's residents and visitors[3]. This data layer is included to represent regional access to nature through parks and recreational areas.

Parks and Recreation Lands data layer

This layer includes a total of 1,012 national, state or provincial, county, and local public places and parks that were identified and included in the analysis. Public lands with public access in the United States were obtained from the Conservation and Recreation Lands (CARL) database, which was developed by the Great Lakes-Atlantic Regional Office of Ducks Unlimited. Ontario protected lands were compiled from several sources that collectively include provincial parks, conservation areas, and federal lands. We computed the amount of park and recreation land in each hexagon using an intersect process in GIS.

Data sources & potential limitations

The CARL database is available online (June 2007; accessed February 2008). Additional Ohio data was provided by the Ohio DNR Division of Wildlife, Toledo Metro Parks, and the Sandusky County Park District. The Canadian parks and recreation dataset was obtained through Nature Conservancy of Canada (NCC; personal communication). Data for Ontario provided by NCC was compiled from a number of sources, with provincial parks obtained from the Ministry of Natural Resources (2008) and federal lands from the Canadian Wildlife Service (2005). The Essex Region Conservation Authority provided data directly for conservation lands (received March 2014) and requires the following disclaimer; "The location and characterization of these sites as shown are approximate and are subject to change; Copyright ERCA 2012".

Potential limitations with this dataset include the fact that not all parks or recreation lands are included in the CARL data or Ontario lands and both the quality and completeness of all data sets used varies across the WLEB.

References & Links

1. Smith et.al. 2013. Relating ecosystem services to domains of human well-being: foundation for a U.S. index. *Ecological Indicators*, 28: 79-90.
2. Pearsall, D., *et al.*. 2012. "Returning to a Healthy Lake: Lake Erie Biodiversity Conservation Strategy." Technical Report. A joint publication of The Nature Conservancy, Nature Conservancy of Canada, and Michigan Natural Features Inventory. 340 pp. with Appendices. http://www.conservationgateway.org/ConservationByGeography/NorthAmerica/wholesystems/greatlakes/Pages/lakeerie.aspx
3. Personal communication with Ohio Department of Natural Resources.

Recreational Boating

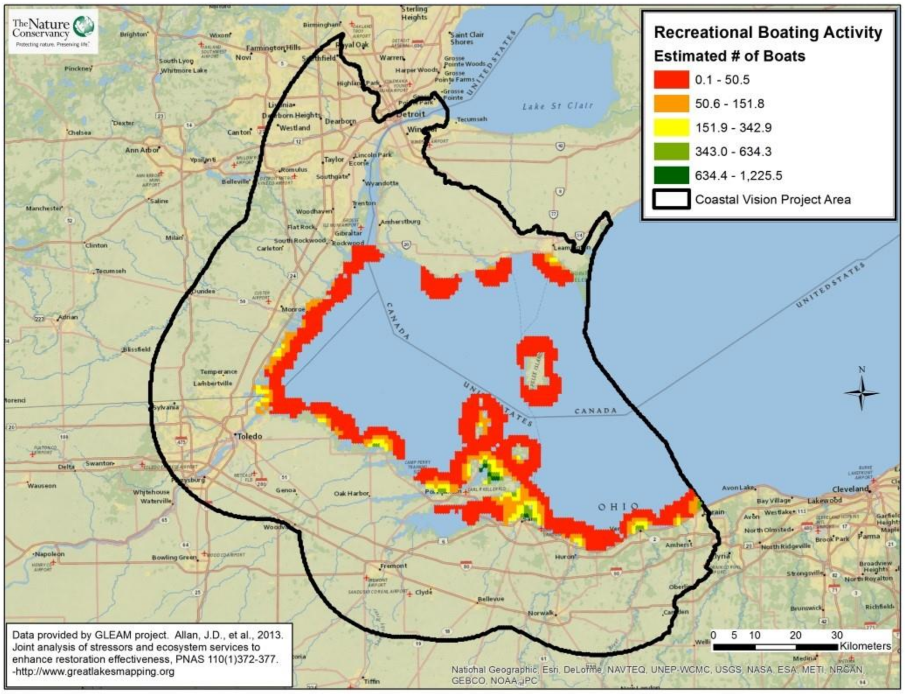

Recreational Boating. *The estimated number of boats on the water near marinas is shown on a gradient from green to red, with green being the highest density of boats. The WLEB analysis area is outlined in black.*

Recreational boating in relation to social and ecological issues

Boating offers individuals a variety of opportunities to recreate on the water, ranging from fishing to waterskiing to sailing. Recreational boating attracts visitors and other sportsmen from across the country to the Western Lake Erie Basin (WLEB). Recreational boating comprises a significant segment of the recreational use of the WLEB, with the Great Lakes Commission estimating that 2.56 million boats were registered in Michigan, Ohio and Ontario alone[1]. In total, over one-third of all boats registered in the U.S. call a Great Lakes state home. Pleasure-craft users average $15,626 in annual spending related to boating, directly supporting over 107,000 jobs throughout the Great Lakes[2]. The success of the boating industry in western Lake Erie is highly-dependent on lake levels, as many marinas are connected to the lake by very shallow channels. Since the early 2000's, Lake Erie has experienced near-record low lake levels, straining already depleted funds for dredging, closing boat slips in marinas and grounding vessels[3]. In 2016, lake levels have rebounded to more typical levels, however further stress on the ecosystem through climate change could impact lake levels and recreational boaters in the long-term. The recreational boating data layer was included in the Marxan analysis to highlight waters within the WLEB that are considered valuable by boaters.

Recreational Boating data layer

This layer shows the estimated density of boaters in the WLEB, with the greatest density of boats expected nearest to marinas. The number of boating slips at each marina in the WLEB was obtained

from the GLEAM research team, who multiplied by the average slip occupancy rate for 2004, and extrapolated to reflect decreased boating activity as distance from a marina increases.

Data sources and potential limitations

The recreational boating data layer was obtained through the Great Lakes Environmental Assessment and Mapping Project (GLEAM; received August 2013)[4]. This dataset may not be truly indicative of highly valued waters to boaters, as it is consists of predictions based on boat slips rather than on actual data gathered for boating activity. Recreational boating activity within the Detroit River was not available.

References and links

1. http://glc.org/files/docs/2007-rec-boating-economic-punch.pdf
2. http://www.greatlakes-seaway.com/en/recreational/cruising-the-seaway/cruises_facts.html and http://glc.org/files/docs/2007-rec-boating-economic-punch.pdf
3. http://www.glerl.noaa.gov/data/now/wlevels/dbd/
4. Allan, J.D., et al. (2013) Joint analysis of stressors and ecosystem services to enhance restoration effectiveness, PNAS 110(1) 372-377. Available online via: http://www.greatlakesmapping.org

Shipwrecks (Dive Sites)

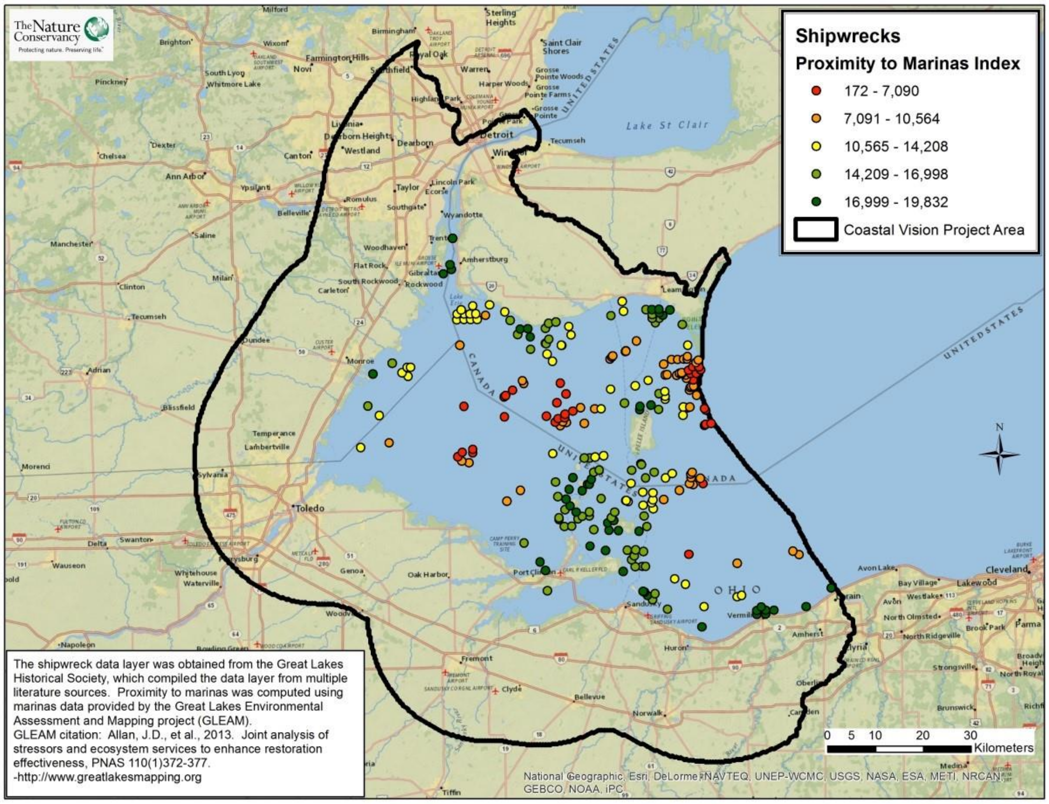

Shipwrecks (Dive Sites). *Here shipwrecks are shown as points colored according to predicted visitation rates based on proximity to marinas, with green indicating high predicted visitation rates and red indicating low rates.. The WLEB analysis area is outlined in black.*

Shipwreck dive sites in relation to regional ecological and social values

There are more than 6,000 shipwrecks in the Great Lakes, with 286 in the Western Lake Erie Basin (WLEB). Shipwrecks are considered an irreplaceable cultural resource that provides a historical record for researchers and freshwater recreation for residents in the Great Lakes. Shipwrecks include military, commercial, and private vessels and are managed as a public trust. With the strong support of local diving groups, Ontario, Ohio, and Michigan have passed laws that prevent the removal or damage of shipwrecks and related artifacts in the Great Lakes[1]. Archaeologists and recreational divers value the pristine condition of shipwrecks in the lakes' fresh, cold waters relative to those found in salt water. Many of the shipwrecks in Lake Erie are located in shallow, easily accessible waters, which increase the appeal of these sites for divers. Divers drawn to Lake Erie often rent gear, take charter boats to wreck sites, and pay for food and lodging during their trip, which contributes to the WLEB tourism revenue; in 2011, the Lake Erie coastal and island tourism industry as a whole brought $1.6 billion to Ohio alone[2]. A survey of natural resource professionals in the WLEB found that recreation and tourism is the most highly valued service provided by Lake Erie. Shipwrecks have been included in the analysis in recognition of their recreational and historic importance, and their corresponding economic value to the region.

Shipwrecks (Dive Sites) data layer

This data layer shows shipwreck locations in relation to marinas throughout the Western Lake Erie Basin (WLEB). In the analysis, the shipwrecks included in this layer were valued according to their proximity to marinas, with the assumption that wrecks closest to marinas will have the greatest value to local economies. The shipwreck data layer was obtained from the Great Lakes Historical Society, which compiled the data layer from multiple literature sources. The marinas layer was compiled by the Great Lakes Environmental Assessment and Mapping Project (GLEAM) and used for the valuation of individual shipwrecks.

Data sources & potential limitations

The shipwreck data layer was obtained from the Great Lakes Historical Society, which compiled the data layer from multiple literature sources. Proximity to marinas was computed using marinas data provided by the Great Lakes Environmental Assessment and Mapping Project (GLEAM)[3].

References & Links

1. http://www.michigan.gov/deq/0,4561,7-135-3313_3677_3701-14514--,00.html ; http://seanags.com/WreckLaw.pdf ; http://www.mynewwaterfronthome.com/shipwrecks.aspx
2. Bauer, J. 2012. Economic impact of tourism in Lake Erie shores & islands reveals nearly $1.6 billion in visitor spending. Lake Erie Shores & Islands.
3. Allan, J.D., et al. 2013. Joint analysis of stressors and ecosystem services to enhance restoration effectiveness, PNAS 110(1)372-377. <http://www.greatlakesmapping.org>.

Recreational Fishing

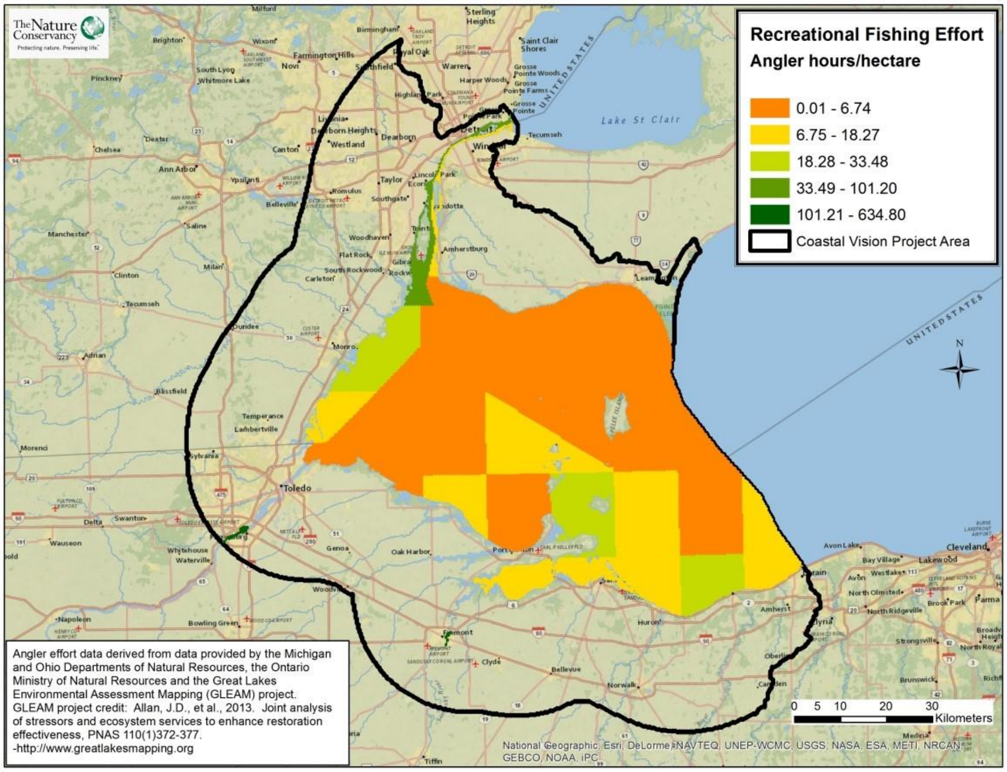

Recreational Fishing. *The density of recreational fishing effort is represented by angler-hours per hectare, with green indicating the greatest number of angler-hours and red indicating the least number. The WLEB study area is outlined in black.*

Recreational fishing in relation to regional ecological and social values

Recreational fishing provides an opportunity for people to interact with nature. Some studies have found that recreational fishing is the most popular outdoor activity among adults, with over 1,664,000 anglers fishing on the Great Lakes in 2011[1]. Thirty-eight percent of those anglers chose to fish in Lake Erie during 2011—this is 10 percent more than the number of anglers who visited Lake Michigan, making Lake Erie the most popular fishing destination in the Great Lakes Basin[2]. Fishing provides participants with light to moderate exercise, access to nature, and an important food source. Fishing within the Western Lake Erie Basin (WLEB) is also an important source of income and revenue, with the expenditures for a typical day trip on Lake Erie totaling between US$42-98 per angler[3]. Between 2006 and 2011, the number of anglers fishing in the Great Lakes region increased by 17 percent and contributed US$1.9 billion to the Great Lakes' economy, illustrating the increasing importance of fishing to the local economy[4]. Recreational and sport fishing also provides ecological benefit to the region in the form of tax revenues generated from the sale of fishing licenses, a portion of which is dedicated to habitat preservation and restoration. Numerous sport fishing tournaments take place within the WLEB on an annual basis, giving fisherman from around North America the chance to compete on some of the most abundant fishing grounds in the Great Lakes. Walleye are so highly valued by the sport fishing industry in the United States that commercial fishing interests are not permitted to catch walleye on the

American side of the border[5]. This layer was used to ensure that this highly valued activity was included in the analysis.

Recreational Fishing data layer

The recreational and sport fishing layer represents the fishing value of WLEB waters in terms of angler-hours, which vary slightly between the Ontario, Ohio, and Michigan areas of the WLEB. The WLECCV Project team compiled this layer from data provided by the Michigan and Ohio Departments of Natural Resources, the Ontario Ministry of Natural Resources, the Great Lakes Environmental Assessment and Mapping (GLEAM) project, and several published creel surveys. The assessment unit size for the U.S. and Canadian sides of the lake, and for the rivers, varied substantially; for example, the U.S. side of the lake is divided into 5-minute grid cells, while the Canadian side is divided into several uneven assessment units. All areas of the WLEB, including the Detroit, Maumee, and Sandusky Rivers, were converted into hectares to create a uniform data layer for the analysis. Similarly, angler-effort data for a mix of walleye, yellow perch, white bass, and smallmouth bass were received from several sources of varying measurement units that were subsequently converted into angler-hours for the analysis.

Data sources & potential limitations

The recreational fishing data layer was compiled using data from a number of different sources. Angler-hour data for fishing of all species in the Michigan portion of Lake Erie were obtained through personal communication with the Michigan Department of Natural Resources. Angler-hour data (2008) for walleye, yellow perch, white bass, and smallmouth bass fishing were obtained through communication with the Ohio Department of Natural Resources. The Ohio DNR also provided angler-hour data (2013) for walleye fishing in stretches of the Maumee and Sandusky River that they had previously identified as the most intensely fished areas. Angler-hours for walleye, yellow perch, and smallmouth bass fishing in the Ontario portion of Western Lake Erie were digitized based on data provided in a 2008 survey by the Ontario Ministry of Natural Resources[6]. Detroit River angler-hours for walleye, yellow perch, and white bass fishing on the Canadian side were digitized from a 2009 Creel survey by the Ontario Ministry of Natural Resources[7]. Angler-hours for the Michigan side of the Detroit River were provided through personal communication with a member of the Great Lakes Environmental Assessment and Mapping (GLEAM) project; this data includes both charter fishing (2010-2012) and private fishing (2002-2004). No angler effort data was available for Michigan or Ontario streams.

References & Links

1. American Sportfishing Association (2013) Sportfishing in America: An Economic Force for Conservation. Accessed online on 2/18/2014 via http://asafishing.org/facts-figures/sporfishing-economics/sportfishing-in-america/
2. U.S Department of the Interior, U.S. Fish and Wildlife Service, and U.S. Department of Commerce, U.S. Census Bureau. 2011 National Survey of Fishing, Hunting, and Wildlife-Associated Recreation. www.census.gov/prod/2012pubs/fhw11-nat.pdf
3. U.S. data gathered from U.S. Fish and Wildlife Service. 2011. National Survey of Fishing, Hunting, and Wildlife-Associated Recreation. Michigan and Ohio Summary Files. Accessed online on 2/19/2014 via http://www.census.gov/prod/www/fishing.html. Canadian data gathered from Fisheries and Oceans Canada.

2008. Survey of Recreational Fishing in Canada: Selected Results for the Great Lakes Fishery, 2005. Accessed online on 2/19/2014 via http://www.dfo-mpo.gc.ca/stats/rec/gl/index-eng.htm.

4. U.S. Fish and Wildlife Service. 2011 National Survey of Fishing, Hunting, and Wildlife-Associated Recreation-National Overview. August 2012. USFWS. www.doi.gov/news/pressreleases/upload/FWS-National-Preliminary-Report-2011.pdf ; U.S Department of the Interior,
   U.S. Fish and Wildlife Service, and U.S. Department of Commerce, U.S. Census Bureau. 2011 National Survey of Fishing, Hunting, and Wildlife-Associated Recreation. www.census.gov/prod/2012pubs/fhw11-nat.pdf
5. www.miseagrant.umich.edu/explore/about-the-great-lakes/lake-erie/
6. Belore, M., K. Ho, and R. Drouin. 2008. Summer angler survey in the Canadian waters of the Western Basin of Lake Erie. Ontario Ministry of Natural Resources: Lake Erie Management Unit.
7. Soper, K., and B. Locke. 2010. Summer creel census in the Canadian waters of the Detroit River. Ministry of Natural Resources: Lake Erie Management Unit.

Trails

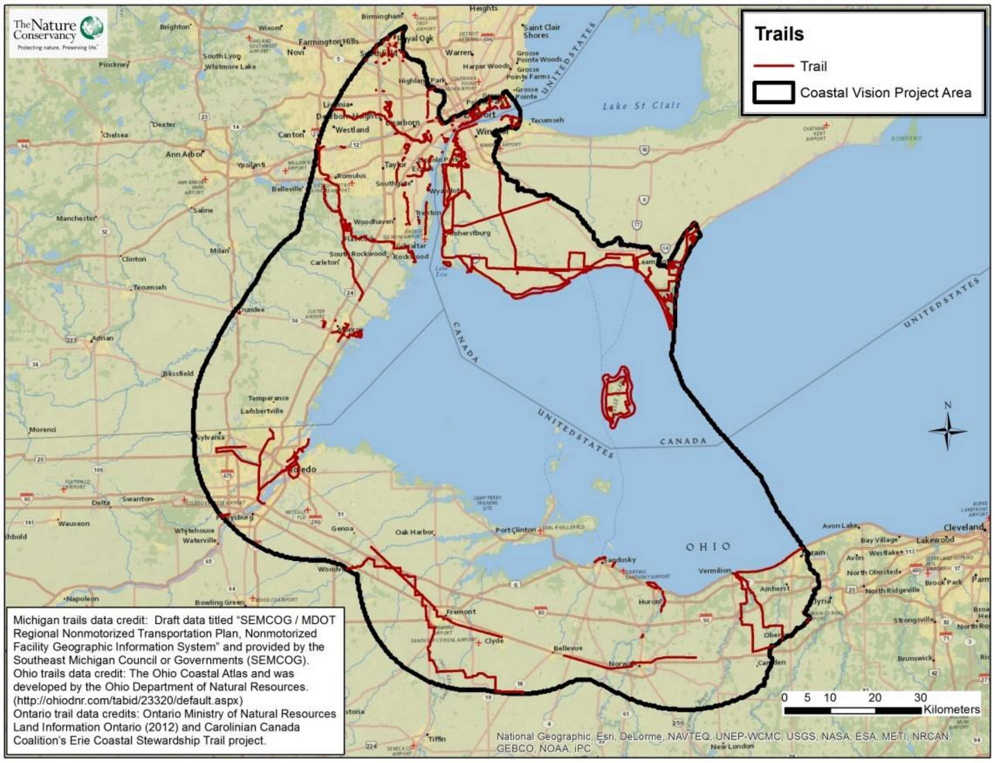

Trails. *Here trails within the WLE watershed and across Ohio are shown in purple. The WLEB analysis area is outlined in black.*

Trails in relation to regional ecological and social values

The Western Lake Erie Basin (WLEB) is home to hundreds of miles of trails that are accessible to a wide range of recreational users. Trails benefit human health by providing an excellent opportunity to engage in aerobic activity, contributing to reductions in heart disease, obesity, diabetes, and even hypertension and anxiety[1]. In addition to providing an opportunity for exercise, these trails provide economic vitality, access to nature, cultural awareness, and opportunities for socializing. A survey of natural resource professionals and conservation practitioners conducted as part of the Lake Erie Biodiversity Conservation Strategy (LEBCS) revealed that the most important ecosystem service provided by Lake Erie and its coastal area is recreation and tourism[2]. Trails like the Lake Erie Birding Trail facilitate birding and recreational activities that fuel the region's valuable tourism economy; tourism in Ohio's seven coastal counties, for example, brings in annual $11.5 billion[3]. In recent years, trails have become a focus of local and regional planning efforts, not only for social and economic benefits, but also for their important environmental benefits, such as providing habitat for animals and plants and reducing habitat fragmentation by connecting areas of conservation lands[4]. The Michigan Department of Transportation (MDOT) and the Southeast Michigan Council of Governments (SEMCOG) are currently creating regional plans for non-motorized trails that will cover all 7 counties in the region, in recognition of the importance of trails in the well-being of residents. A similar effort is being undertaken in Ontario, which has developed the Ontario Trails Strategy to increase collaboration and access to trails for residents throughout the province. This layer includes trails like the Ohio's statewide Buckeye Trail, Michigan's

popular Hines Park Bikeway, and Ontario's 31 mile (50 km) Chrysler Canada Trail and Pelee Island Winery Trail. These trails and others were included in the analysis in recognition of their importance to the people of the WLEB.

Trails data layer

National, state or provincial, county and local trails that provide public access to nature were considered in the analysis. This data layer includes both existing and planned hiking trails, cycling trails, and trails for both hiking and cycling. This may include features such as shared roadways (roads including bike lanes) or gravel-surfaced trails that may not be suitable for all uses. Approximately 734 miles (1,181 km) of trails were identified within the WLEB for inclusion in the analysis. Trails were attributed to hexes on simple presence-absence basis; no values were assigned to hexes without trails.

Data sources & potential limitations

Trails data for Canada (2012) were produced by the Ontario Ministry of Natural Resources Land Information Ontario[6] and distributed by Andrea Hebb of Nature Conservancy Canada (received August 2013). Additional Ontario data was provided by Carolinian Canada Coalition's Erie Coastal Stewardship Trail project and also distributed by Andrea Hebb (received October 2014). Data for Michigan trails was obtained from the Southeast Michigan Council of Governments[7] (SEMCOG; draft, received 18 Feb 2014), and data for Ohio came from the Ohio Department of Natural Resources[8] (OH DNR; Dec 2004, received 24 Jun 2013). Both existing and proposed trails were included in the Michigan and Ontario databases. This data layer potentially underrepresents the true number and use of trails within the region because some uses, such as cross-country skiing, were not considered in this analysis due to lack of readily available data. Water trails also were not generally included in this analysis, though some water trails were in the Ontario data.

References & Links

1. American Hiking Association (2010) Health Benefits of Hiking. Accessed online on 2/3/2014 via: http://www.americanhiking.org/wpcontent/uploads/2013/04/Heath-Benefits-of-Hiking-fact-sheet.pdf
2. Fifty-six lake managers, conservation practitioners, academic and other experts participated in the LEBCS survey.
3. http://www.lakeerieimprovement.org/wp-content/uploads/2012/02/leia-strategic-plan-final-12-17-2012.pdf
4. Hess, G.A. and Fischer, R.A. (2001) Communicating clearly about conservation corridors. *Landscape and Urban Planning.* Vol. 55, No. 3, 195-208.
5. Downriver Linked Greenway Initiative (2013) Trail Segments. Accessed online on 2/3/2014 via: http://www.downrivergreenways.org/home/trail-segments
6. Accessible online via: http://www.mnr.gov.on.ca/en/Business/LIO/2ColumnSubPage/STEL02_167956.html
7. http://www.semcog.org/
8. http://www2.ohiodnr.gov/

Water Access Sites

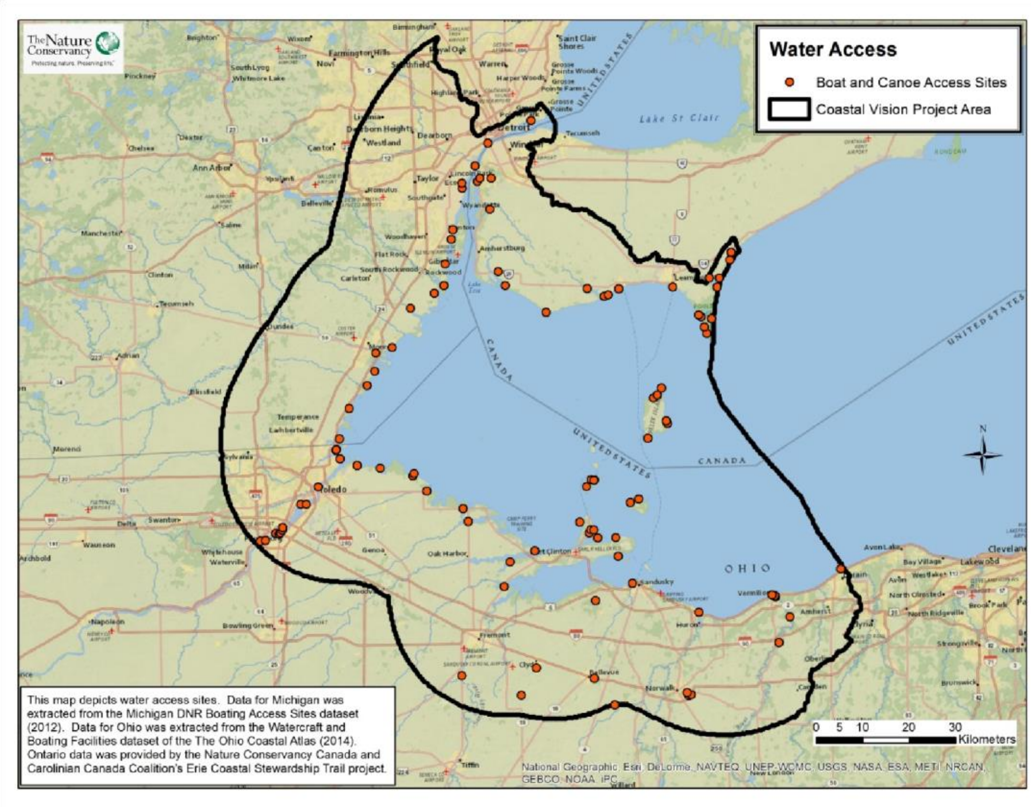

Water Access Sites: Public water access sites are indicated by orange points on the map. The study area is outlined in black.

How are water access sites in Lake Erie connected to ecological and social values?

Access to water is an important recreational value to many people in the region. Water access sites provide individuals a variety of opportunities to recreate on the water, from boating and fishing to canoeing and kayaking. Recreational boating attracts visitors and other sportsmen from across the country to the Western Lake Erie Basin (WLEB). Boating comprises a significant segment of the recreational use of the WLEB, with the Great Lakes Commission estimating that 2.56 million boats were registered in Michigan, Ohio and Ontario alone[1]. In total, over one-third of all boats registered in the U.S. call a Great Lakes state home. Pleasure-craft users average $15,626 in annual spending related to boating, directly supporting over 107,000 jobs throughout the Great Lakes[2]. The economic value of public access ramps can be very high[4]. The water access sites data layer was included in the Marxan analysis to highlight areas within the WLEB that are considered valuable by boaters, canoeists, and kayakers who do not typically utilize marinas.

Water access sites data layer

The WLECCV team compiled this data layer from a number of state and provincial sources. Marinas and private facilities were generally excluded. In total, 100 public water access sites were identified and included in the Marxan analysis.

Data sources and potential limitations

Data for Michigan was extracted from the Michigan DNR Boating Access Sites dataset (2012). Data for Ohio was extracted from the Watercraft and Boating Facilities dataset of the Ohio Coastal Atlas (2014). Ontario data was provided by the Nature Conservancy Canada and Carolinian Canada Coalition's Erie Coastal Stewardship Trail project (data received October 2014). It is possible that some public water access sites were not represented in the source data.

References and links

1. http://glc.org/files/docs/2007-rec-boating-economic-punch.pdf
2. http://www.greatlakes-seaway.com/en/recreational/cruising-the-seaway/cruises_facts.html and http://glc.org/files/docs/2007-recboating-economic-punch.pdf
3. http://www.glerl.noaa.gov/data/now/wlevels/dbd/
4. Harding, D., J. Wiggin, E. Mahoney, D. Bordner, D. Stynes, M. Thomas, and F. Lupi. 2009. Florida Boating Access Facilities Inventory and Economic Study including a Pilot Study for Lee County. Executive Summary. Florida Fish and Wildlife Conservation Commission. Tallahassee, Florida. iv + 20 p. http://myfwc.com/media/1162723/About_Econ_BAFI_Summary.pdf

Costs:

Marxan achieves an optimal solution by selecting geographic areas that meet user defined goals for features while minimizing costs. For the WLECCV analysis, Marxan balanced the contribution of each 10ha planning unit towards achieving the goals for each feature against any associated costs. To represent the costs of implementing conservation and restoration actions, we identified seven cost layers, four of which are characterized in monetary values derived from conservation and restoration projects, and three of which are indices reflecting landscape attributes that affect the feasibility of effective conservation (Table A1). Each cost layer is summarized below in terms of its relevance to conservation, the methods for constructing the layer, and a few additional details.

Table A1. Costs of implementing conservation and restoration actions in the coastal areas of western Lake Erie.

Cost	Units
Land value	$
Wetland restoration cost	$
Phragmites treatment cost	$
Marinas cost to wetlands	Index
Lake Erie and Detroit River stress index	Index
Landbird habitat restoration cost	$
Walleye stream habitat restoration cost	Index

Land Value

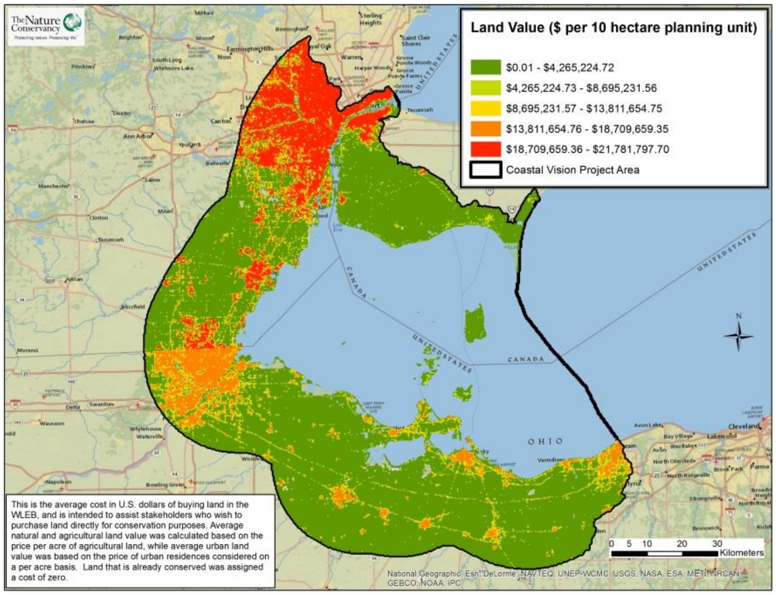

This layer comprises the average cost in US$ of buying land in the WLEB coastal area, and is intended to assist stakeholders who wish to purchase land directly for conservation purposes. For most of the project area, average values for undeveloped and agricultural lands were calculated based on the price per acre of agricultural land, while average urban land value was based on the price of urban residences considered on a per acre basis. This cost layer applies to the entire geography, making it possible for stakeholders to determine the approximate cost of purchasing land in various places in the basin. For example, someone interested in creating a new wetland area can see from this cost layer that buying a parcel of land near the mouth of the Maumee River is significantly more expensive than buying land further east along the southern coast of Lake Erie. With this information, it is now possible for this stakeholder to consider the cost of the parcel when determining the optimal location for the new wetland.

Data layer construction

This layer was constructed from average costs of agricultural and developed lands in the WLEB. The cost of agricultural and other undeveloped land was calculated using the average cost of agricultural land in Ohio[4], Michigan[2], and Ontario[3] (Table A2). The cost of developed land was based on the median home values in the region, as per acre urban land values were not found to be available. Developed land values were computed separately for Michigan, Ohio, and Ontario. For the U.S., we first calculated median home value by county using the value of homes owned by residents in the 45-54 age class, which is the class with highest valued homes. This data was obtained from the City-Data website (www.city-data.com). We assumed that the average home occupied a ¼ acre lot, so the final average home value was multiplied by 4 to give a per acre land value. In Ontario, the cost of urban land was based on the average listing price of a four bedroom home in Windsor, Ontario, for 2013 obtained from a report by Coldwell Bankers[4]. The land value on inhabitable islands in the Ohio portion of Lake Erie was calculated separately, due to the higher value of islands relative to the mainland. We obtained recent costs of conservation transactions for inland and lake front properties on several of these islands from the Lake Erie Islands Chapter of the Black Swamp Conservancy, and then adjusted

[4] http://ohioagmanager.osu.edu/farm-rents/farmland-value-and-rent-outlook-2013/

the land values to align with these transactions. Land on the Ontario and Michigan islands was also evaluated and found to have very similar value to the mainland areas, and therefore were not treated separately. Lands already in conservation ownership were assigned a value of US$0.00 since they are already protected. This included most public lands, conservation lands, and private hunt club lands.

Table A2. Values used in each jurisdiction and category in the Land Value cost layer.

Jurisdiction	Category	Value ($US per acre)
Michigan	Ag/natural	$4,220
Michigan	Urban	$879,720; ($219,930/home)
Ohio	Ag/natural	$5,000
Ohio	Urban	$685,872; ($171,468/home)
Ohio	Lake Erie Islands (undeveloped)	$7,091
Ontario	Ag/natural	$7,029
Ontario	Urban	$757,680; ($189,420/home)

Data Details

It would be ideal to build this data layer from sources that give urban land values per acre; in the future this layer may be rebuilt with census data that was previously unavailable. Median home values have been used as a proxy for urban land value, but the type of data available is inconsistent between countries, with median values divided by age class and county for the U.S. but only an overall median home value is available for Ontario. Using averages across the landscape gives a general picture of costs, but does not account for small-scale variations, such as the difference in land value in business versus residential districts. The contact for this data layer is Gust Annis.

2 https://www.msu.edu/~steind/2012%20MI%20Land%20Values%20Leasing%20Rates%20NO%20643_Final%20Version.pdf

3 Agricultural land values in the Essex region of Ontario were estimated based on the Essex Forests and Wetlands Natural Area Conservation Plan (Nature Conservancy Canada, 2009).

4 http://hlr.coldwellbanker.com/PressRelease.html

Wetland Restoration Cost

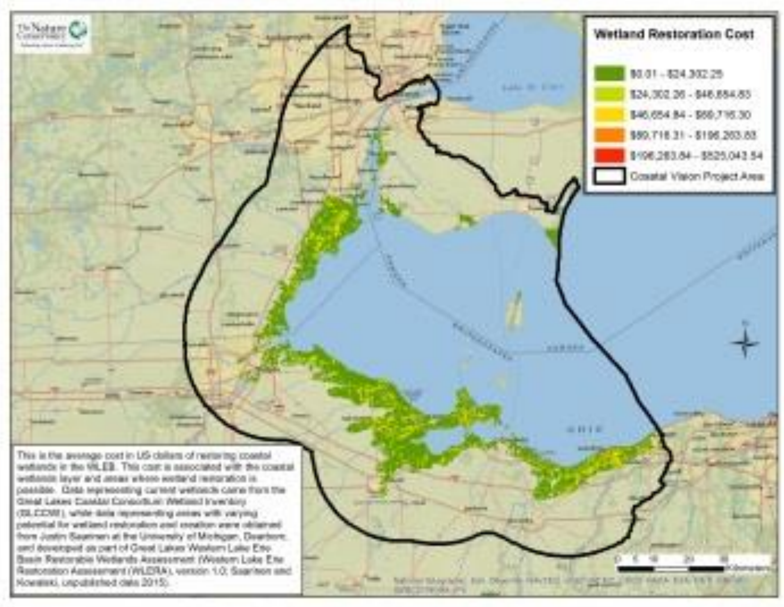

This is the average cost in US$ of restoring coastal wetlands in the WLEB. This cost is associated with the current and restorable coastal wetlands layer, which shows current wetlands and areas where wetland restoration is possible. The cost layer is designed to inform stakeholders of the expense of restoring wetlands along the coast by considering the size, restoration potential, and number of owners of a given coastal parcel. The cost layer also incorporates the average cost of restoration activities associated with all wetland restoration projects, such as native plant restoration and the removal or construction of water control structures like dams, culverts, and channels. A high cost planning unit, for example, is a unit with a low restoration potential, a large area, and many associated land owners, due to the difficulty of negotiating with many owners, and of actually carrying out restoration work across a large area that is not easily converted to wetland habitat. This layer is intended to help stakeholders determine the most cost-effective locations for wetland restoration work along the Lake Erie coast.

Data Layer Construction

The data used to calculate this layer includes four major components. The first is the Current and Restorable Coastal Wetlands layer, created by project staff to provide the acreage of current[5] and potential coastal wetlands. Next is the average restorability index, which indicates the level of difficulty of restoring wetlands and was provided as part of the Western Lake Erie Restoration Assessment by Justin Saarinen of the University of Michigan Dearborn and Kurt Kowalski of the US Geological Survey and covers the US coast except for the Detroit River. For the remaining US and Ontario coastal area, project staff developed a surrogate restorability index following the same principles but using coarser data. The third component is the number of owners in a given planning unit. The final component is a generalized cost estimate of $6,000 per acre for wetland restoration, provided by restoration practitioners Christopher May and James Cole. The data components for this layer are very complete, but it should be noted that the wetland restorability layer for the Detroit River corridor and the Ontario coast is rough; replication of the Western Lake Erie Restoration Assessment layer should be a priority.

---

[5] Obtained from the Great Lakes Coastal Wetland Consortium's Wetland Inventory (http://www.glc.org/wetlands/inventory.html).

Data Details

The wetland restorability index and number of owners per unit area were each divided by 50 to ensure that the resulting values fit a reasonable scale. This layer is complete, but there are notably extreme values at a fine scale in some areas; for example, many of the planning units in urban areas have very high costs due to high ownership complexity. As stated above, opportunities to replicate the wetland restorability index in Canada need to be explored. In general, there is a concern that the costs associated with wetland restoration are relatively complete while the costs of restoration of surrounding areas has not been accounted for; this “stacking up” of costs may cause Marxan to be biased against restoration in some wetland areas in favor of areas where costs have not yet been assigned. The contact for this data layer is Doug Pearsall.

Phragmites Treatment Cost

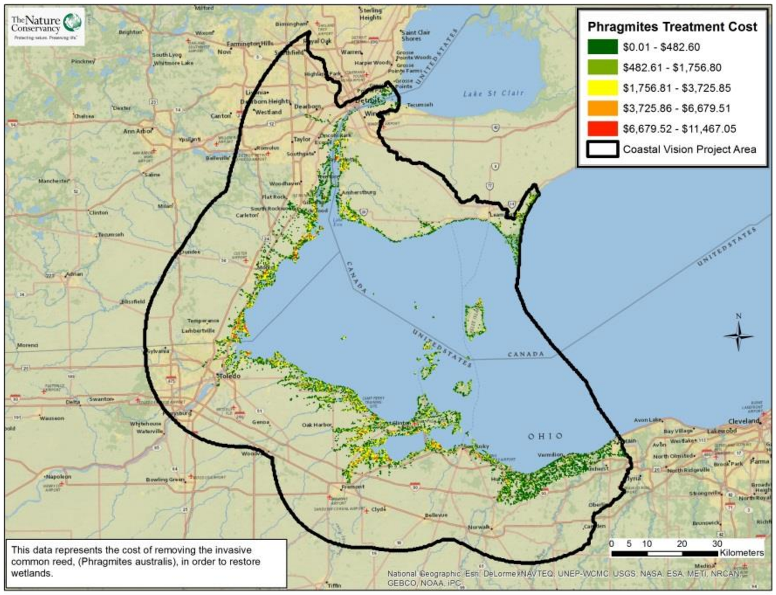

This layer estimates the cost of removing the invasive common reed, (*Phragmites australis*), in order to restore wetlands. The on-the-ground removal method considered in this layer is a three year process involving aerial spraying of Phragmites in the first year, and follow-up spot removal of remaining Phragmites during the following two years. This process is estimated by experienced TNC practitioners to cost approximately $500 per acre. Areas with high treatment costs therefore reflect areas with large amounts of Phragmites, which means removing all Phragmites will be expensive.

Data Layer Construction

Data depicting the location of *Phragmites* along the entire coast of the WLEB were developed by the Michigan Tech Research Institute[6]. This layer was constructed by multiplying each acre of *Phragmites* by $500, the estimated cost of removing *Phragmites*. The *Phragmites* data captures patches of that are larger than 0.2 ha, which is acceptable for this analysis but inevitably misses numerous emerging infestations.

Data Details

This data layer was derived from separate US and Canadian sources. The contact for this data layer is Doug Pearsall.

---

[6] This institute also developed a website with further information about Phragmites: http://www.mtri.org/phragmites.html.

Marinas Cost to Wetlands

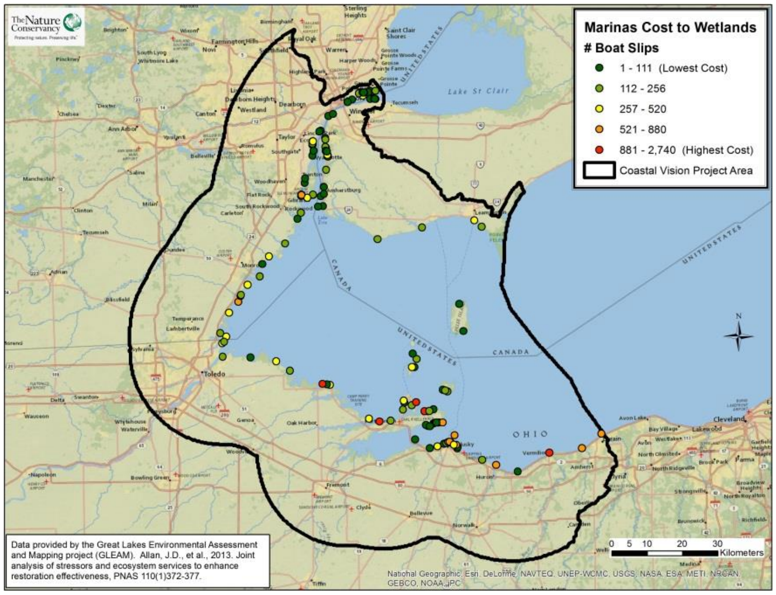

This layer shows where the presence of marinas hinders the restoration of coastal wetlands. Docks and boating ramps in marinas cause shoreline hardening and take up space that might otherwise be filled with wetland plants, and intensive activity associated with marinas creates wakes, pollution, and noise disturbance to wetland wildlife. Conservation practitioners will naturally look to other locations for creating and restoring wetland areas.

Data Layer Construction

This data layer was obtained from the Great Lakes Environmental Assessment and Mapping (GLEAM) Project[7]. GLEAM provided locations and the number of boat slips at each marina. We assumed that marinas with more boat slips would represent a higher cost to conservation, so we derived cost values from the number of boat slips within each project planning unit hexagon. The contact for this data layer is Gust Annis.

[7] Allan, J.D., et al., 2013. Joint analysis of stressors and ecosystem services to enhance restoration effectiveness, PNAS 110(1)372-377. http://www.greatlakesmapping.org

Lake Erie and Detroit River Stress Index

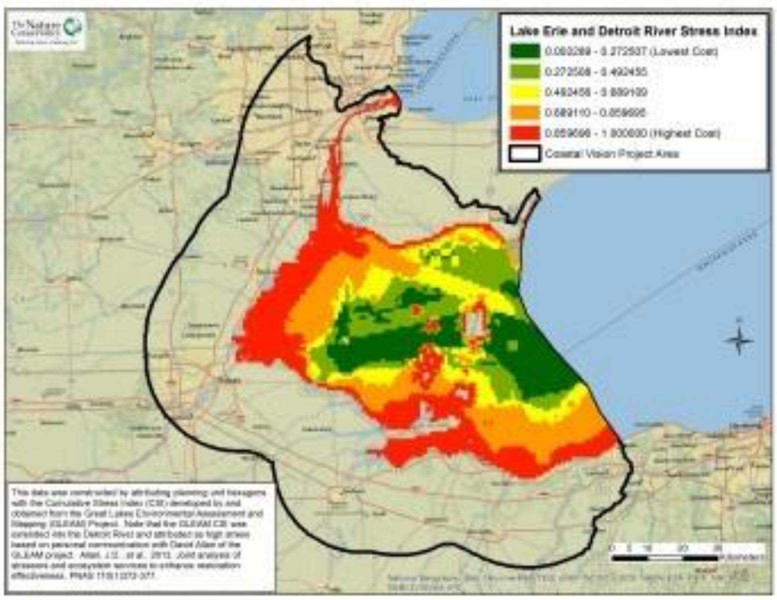

This layer is an index representing multiple stressors that likely have an impact on biota and ecosystem dynamics. The index represents cumulative stress for 34 different stressors which can be physical, chemical, or biological resulting from human activity. According to the Great Lakes Environmental Assessment and Mapping (GLEAM) Project[8], who developed the data, these stressors can be divided into seven different categories including aquatic habitat alterations, climate change, coastal development, fisheries management, invasive species, nonpoint source pollution, and toxic chemical pollution.

Data Layer Construction

The GLEAM research team identified and mapped 34 stressors across the Great Lakes at a 1 km^2 resolution excluding connecting channels. They then developed weightings of the relative impact of each stressor on habitat types using the results of a survey of Great Lakes experts. Finally, the Cumulative Stress Index was developed by summing all of the stressors for each grid cell. A detailed account of this data layer can be found at http://www.greatlakesmapping.org/lakes-stressors. TNC computed and assigned the mean stressor index to each planning unit hexagon in the project area. Note that the GLEAM CSI was extended into the Detroit River and attributed as high stress based on personal communication with David Allan of the GLEAM project. The contact for this data layer is Gust Annis.

[8] Allan, J.D., et al., 2013. Joint analysis of stressors and ecosystem services to enhance restoration effectiveness, PNAS 110(1)372-377. http://www.greatlakesmapping.org

Landbird Habitat Restoration Cost

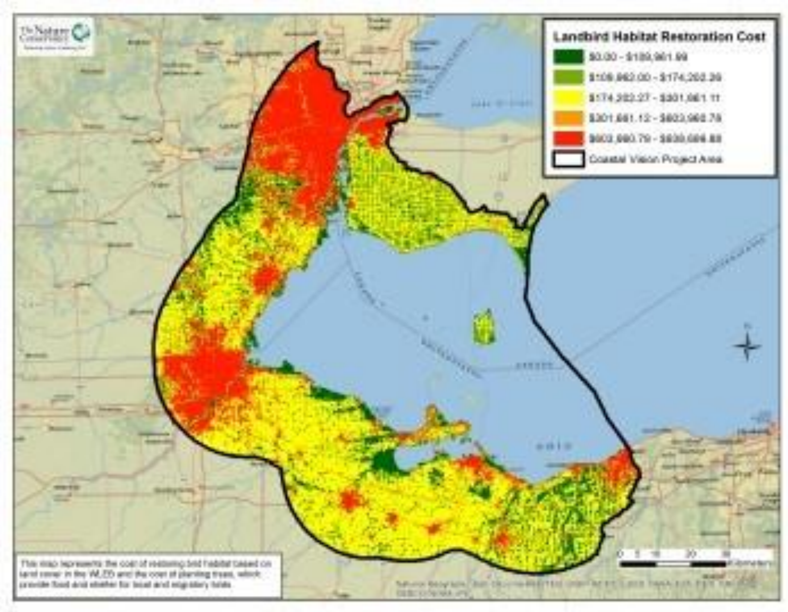

This layer represents the cost of restoring bird habitat based on land cover in the WLEB and the cost of planting trees, which provide food and shelter for local and migratory birds. This layer is applied across the inland geography, as it is intended to relate specifically to landbirds. The cost of restoring landbird habitat is estimated at US$7,441 per acre, adjusted to account for the influence that variation in current land cover will have on restoration cost. Urban areas were considered to have a high cost for restoration, agricultural areas a relatively low cost, and natural areas to have almost no cost. Stakeholders interested in planting trees and shrubs to restore landbird habitat will find this layer useful.

Data Layer Construction

This data layer was produced by first estimating the per-acre cost for restoring landbird habitat. We based the estimate on three actual restoration projects, two of which were implemented on DTE Energy property with costs of $9,000 and $8,000 per acre, respectively[9]. The third project was implemented by TNC's Oak Openings program in Ohio, and cost $5,324 per acre[10]. The average cost of these three projects – rounded to the nearest $100 – provides our estimate of $7,441 per acre. We then established a multiplier based on land cover data from the NOAA Coastal Change Analysis Program (CCAP)[11] for the U.S. and the Ontario Ministry of Natural Resources Provincial Land Cover (PLC) project[12] for Canada; the PLC data lacked roads, so we "burned" roads into the PLC using infrastructure data accessed through Land Information Ontario. We classified these data into three categories including natural, agricultural, and urban land cover, and multiplied the base cost estimate by the average multiplier value within each 10ha planning unit.

Data Details

In order to scale the cost of restoration, a multiplier was applied to each of the three defined low, medium, and high cost areas—which are roughly equivalent to natural, agricultural, and urban areas,

---

[9] Tylenda and Cousino, 2013: http://glmigratorybirds.org/action.html#.UnO1p1MljFc

[10] Personal communication, Jennifer Thieme, Conservation Project Coordinator at TNC Kitty Todd Preserve

[11] http://www.csc.noaa.gov/digitalcoast/data/ccapregional/

[12] http://library.mcmaster.ca/maps/ogde_plc.htm

respectively. A multiplier value of 5 was applied to high cost areas, while multipliers of 1 and 0.5 were respectively applied to agricultural and natural land cover. It should be noted that land cover data for Canada did not have as many classes as U.S. data. The contact for this data layer is Doug Pearsall.

Walleye Stream Habitat Restoration Cost

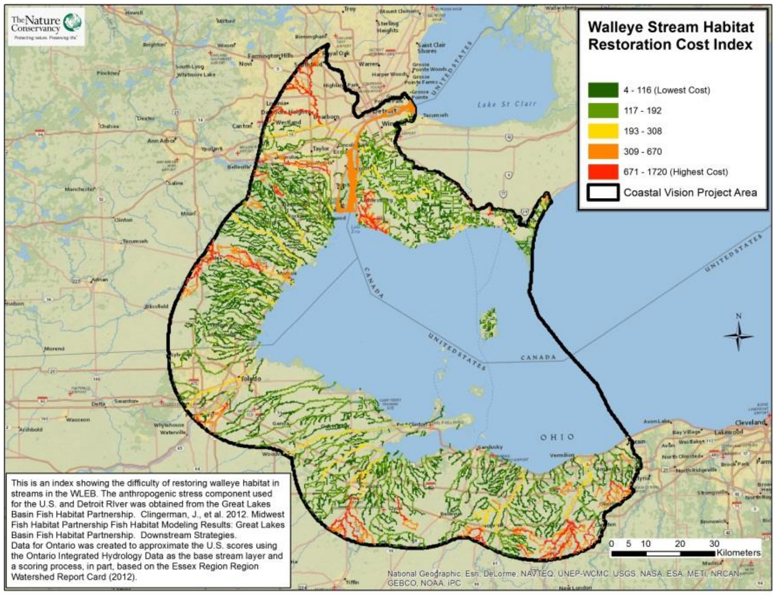

This layer is an index showing the difficulty of restoring walleye habitat in streams in the WLEB. A threat score was assigned to each stream portion using information about barriers to stream flow, size of the stream, and anthropogenic stress on the stream system. Higher costs were assigned to streams of large size, blocked flow, and high levels of anthropogenic stress. This data layer can be used to prioritize streams where restoration projects are likely to have the lowest cost and thus be most successful.

Data layer construction

This data layer was constructed using a formula that multiplied anthropogenic stress, stream size, and stream connectivity together to produce overall costs for restoring various stream areas. The anthropogenic stress component used for this layer for U.S. steams and the Detroit River was obtained from the 2012 Great Lakes Basin Fish Habitat Partnership[13] report by the National Fish Habitat Partnership (NFHAP)[14]. The report calculated a Cumulative Anthropogenic Stress Index (CASI), which was found to be most influenced by stream network wetland land cover, local road crossing density, local groundwater consumption, stream network superfund site density, and local National Pollutant Discharge Elimination System[15] site density. The TNC team multiplied the CASI value for each stream by stream size and by a connectivity score. Stream connectivity and size was determined from TNC data developed for the Lake Erie Biodiversity Conservation Strategy[16]. Connectivity was scored on a

---

[13] Clingerman, J., et al. 2012. Midwest Fish Habitat Partnership Fish Habitat Modeling Results: Great Lakes Basin Fish Habitat Partnership. Downstream Strategies. http://midwestfishhabitats.org/sites/default/files/glb.pdf

[14] http://fishhabitat.org/partnership/great-lakes-basin-fish-habitat-partnership

[15] http://cfpub.epa.gov/npdes/

[16] Pearsall, D., P. Carton de Grammont, C. Cavalieri, C. Chu, P. Doran, L. Elbing, D. Ewert, K. Hall, M. Herbert, M. Khoury, D. Kraus, S. Mysorekar, J. Paskus, and A. Sasson. 2012a. Returning to a Healthy Lake: An International Biodiversity Conservation Strategy for Lake Erie. Technical Report. The Nature Conservancy, Nature Conservancy of Canada, and Michigan Natural Features Inventory, Lansing, MI, 340 pp. with appendices. Available at http://www.conservationgateway.org/ConservationByGeography/NorthAmerica/UnitedStates/michigan/projects/biodiversity/Pages/default.aspx 17

presence-absence basis, while streams were categorized into four size classes. Data for Ontario streams was created to approximate the U.S. scores using the Ontario Integrated Hydrology Data as the base stream layer and a scoring process, in part, based on the Essex Region Watershed Report Card (2012)[17] where streams in watersheds receiving Watershed Health grades of "C" were assigned a score of 50 and those receiving a grade of "D" were assigned a score of 75. Any streams in watersheds with grades of "Unknown" were assigned a score of 75. Then the same process of multiplying these base scores by a stream size and connectivity score as described above for the U.S. streams was applied.

Data Details

Multipliers were applied to the connectivity and stream size scores in order to account for the increased difficulty of restoring streams with barriers to flow and large overall size. In regard to connectivity, a value of 1 was entered for streams connected to the lake and a value of 5 was entered for streams with barriers to flow. For stream size, a multiplier of one was input for the smallest size class, and multipliers of two, three, and four were applied as size class increased, respectively. In the future, it may be necessary to adjust the analysis to account for the connection of polygons that follow a stream's flow, but this is unlikely to be needed. The contact for this data layer is Gust Annis.

Essex Region Watershed Report Card, 2012. http://erca.org/wp-content/uploads/2013/03/watershed-report2012-single-pg-for-web.pdf
