# Supplementary material for: Designing coastal conservation to deliver ecosystem and human well-being benefits
Source: PLoS One. 2017 Feb 27;12(2):e0172458. doi: 10.1371/journal.pone.0172458 (PMC5328270; doi:10.1371/journal.pone.0172458)
Supplement: S1 Table — (PDF) [file pone.0172458.s002.pdf]

S1 Data Source Table:
List of Data Sources Used

Supporting information (S1 Data Source Table) to manuscript:

Designing coastal conservation to deliver ecosystem and human well-being benefits

Gust M. Annis[1*], Douglas R. Pearsall[1], Katherine J. Kahl[1], Erika L. Washburn[1,#a], Christopher A. May[1], Rachael Franks Taylor[1,#b], James B. Cole[2], David N. Ewert[1], Edward T. Game[3], Patrick J. Doran[1]

Feature / Cost	Name	Data description	Data Type	Data source	Contact information	Website	Accessibility	Attribution statements
**Feature**	Spawning Sites	Point locations of spawning sites	Published spatial data	Ohio Department of Natural Resources, Coastal Management Program, Coastal Atlas	Brian George, Ohio Department of Natural Resources, Brian.George@dnr.state.oh.us	http://coastal.ohiodnr.gov/atlas	Available by request	NA
**Feature**	Spawning Sites	Point locations of spawning sites	Unpublished tabular data	Ohio State University; Ohio Department of Natural Resources	Cassie May, PhD student, Ohio State University, may.265@osu.edu		Contact only	NA
**Feature**	Spawning Sites	Point locations of spawning sites	Unpublished tabular data	Ontario Ministry of Natural Resources	Dr. Yingming Zhao, Ontario Ministry of Natural Resources and Climate Change, yingming.zhao@ontario.ca		Contact only	NA
**Feature**	Spawning Sites	Point locations of spawning sites	Published tabular data	US Geological Survey, Great Lakes Science Center	Dr. Edward Roseman, US Geological Survey, Great Lakes Science Center, eroseman@usgs.gov		Contact only	NA
**Feature**	Spawning Sites	Point locations of spawning sites	Published tabular data	US Geological Survey, Great Lakes Science Center	Dr. Jason Fischer, US Geological Survey, Great Lakes Science Center, jrfischer@usgs.gov		Contact only	NA

Feature / Cost	Name	Data description	Data Type	Data source	Contact information	Website	Accessibility	Attribution statements
**Feature**	Walleye Stream Habitat	Line file of streams attributed with habitat value and other parameters	Published spatial data	Midwest Fish Habitat Partnership, Great Lakes Partnership Geodatabase	NA	http://midwestfishhabitats.org/resources/Geodatabase	Link to download data	NA
**Feature**	Walleye Lake Habitat	Bathymetry data	Published spatial data	National Oceanic and Atmospheric Administration, Great Lakes Bathymetry	NA	http://www.ngdc.noaa.gov/mgg/greatlakes/	Link to download data	NA
**Feature**	Potential Coastal Wetlands	Raster data providing an index of coastal wetland restorability for western Lake Erie (US side only)	Published spatial data	Western Lake Erie Restoration Assessment	Justin Saarinen, New College of Florida, jsaarinen@ncf.edu	http://wim.usgs.gov/	Link to download data	NA
**Feature**	Potential Coastal Wetlands	Wetland polygons	Published spatial data	Great Lakes Coastal Wetland Consortium, Coastal Wetland shapefile, currently available through the	Don Uzarski, uzars1dg@cmich.edu	http://greatlakeswetlands.org/Home.vbhtml	Available by request	NA

Feature / Cost	Name	Data description	Data Type	Data source	Contact information	Website	Accessibility	Attribution statements
				Great Lakes Coastal Wetland Monitoring Project				
**Feature**	Potential Coastal Wetlands	Wetland polygons	Private spatial data	Michigan Natural Features Inventory	Becca Rodgers, Michigan State University Extension, Michigan Natural Features Inventory, Rogersr@michigan.gov	http://mnfi.anr.msu.edu/	Available by request	NA
**Feature**	Potential Coastal Wetlands	Coastal land cover	Published spatial data	Michigan Tech Research Institute	Laura Burgeau-Chavez, lchavez@mtu.edu	http://geodjango.mtri.org/coastal-wetlands/	Available by request	NA
**Feature**	Potential Coastal Wetlands	Polygons of coastal wetlands along the Detroit River, circa 1796	Unpublished spatial data	Digitized from a scanned map of the Detroit River produced by General George Henry Victor Collot of the French Navy in 1796; scanned image obtained from the US Geological Survey; original image	NA	https://historicalcharts.noaa.gov/historical_jpgs/00-00-1796.jpg	Link to download data	NA

Feature / Cost	Name	Data description	Data Type	Data source	Contact information	Website	Accessibility	Attribution statements
				scanned by the National Oceanic and Atmospheric Administration				
**Feature**	Coastal Terrestrial Biodiversity Significance	Coastal land cover	Published spatial data	Michigan Tech Research Institute	Laura Burgeau-Chavez, lchavez@mtu.edu	http://geodjango.mtri.org/coastal-wetlands/	Available by request	NA
**Feature**	Coastal Terrestrial Biodiversity Significance	Point and polygon data for rare plants, animals, and natural communities tracked by Heritage programs	Published, restricted spatial data	Michigan Natural Features Inventory	Becca Rodgers, Michigan State University Extension, Michigan Natural Features Inventory, Rogersr@michigan.gov	http://mnfi.anr.msu.edu/	Sensitive data; available only through data use agreement with provider	NA
**Feature**	Coastal Terrestrial Biodiversity Significance	Point and polygon data for rare plants, animals, and natural communities tracked by Heritage programs	Published, restricted spatial data	Ohio Department of Natural Resources, Natural Heritage Database	NA	http://wildlife.ohiodnr.gov/species-and-habitats/ohio-natural-heritage-database	Sensitive data; available only through data use agreement with provider	NA

Feature / Cost	Name	Data description	Data Type	Data source	Contact information	Website	Accessibility	Attribution statements
**Feature**	Coastal Terrestrial Biodiversity Significance	Point and polygon data for rare plants, animals, and natural communities tracked by Heritage programs	Published, restricted spatial data	Ontario Ministry of Natural Resources and Forestry, Natural Heritage Information Centre; copyrighted by the Queen's Printer for Ontario, 04 December 2014.	Jim Mackenzie, Ontario Ministry of Natural Resources and Forestry, Natural Heritage Information Centre, Jim.S.Mackenzie@ontario.ca	https://www.ontario.ca/page/get-natural-heritage-information	Sensitive data; available only through data use agreement with provider	NA
**Feature**	Coastal Terrestrial Biodiversity Significance	NOAA Great Lakes Shoreline	Published spatial data	National Oceanic and Atmospheric Administration, Medium Resolution Digital Shoreline for the Great Lakes	NA	http://www.glerl.noaa.gov/data/char/glshoreline.html	Link to download data	NA
**Feature**	Nearshore Waterfowl Habitat	Current stopover habitat	Published spatial data	Great Lakes Migratory Bird Stopover Portal	August Froehlich, The Nature Conservancy, afroehlich@TNC.ORG	http://www.glmigratorybirds.org/	Link to download data	NA
**Feature**	Restorable Inland	Current stopover habitat	Published spatial data	Great Lakes Migratory Bird	August Froehlich, The Nature Conservancy, afroehlich@TNC.ORG	http://www.glmigratorybirds.org/	Link to download data	NA

Feature / Cost	Name	Data description	Data Type	Data source	Contact information	Website	Accessibility	Attribution statements
	Landbird Habitat			Stopover Portal				
**Feature**	Restorable Inland Landbird Habitat	Coastal Land Cover	Published spatial data	NOAA Coastal Services Center, C-CAP Regional Land Cover Data	NA	http://greatlakesresilience.org/maps-tools-data/data/coastal-change-analysis-program-c-cap-regional-land-cover-data	Link to download data	NA
**Feature**	Restorable Inland Landbird Habitat	Provincial Land Cover	Published spatial data	Ontario Ministry of Natural Resources and Forestry	NA	https://www.javacoeapp.lrc.gov.on.ca/geonetwork/srv/en/main.home?uuid=ed66d203-d5ca-47a2-b357-0226ea3d29ae	Link to download data	NA
**Feature**	Inland Waterfowl Habitat	Current stopover habitat	Published spatial data	Great Lakes Migratory Bird Stopover Portal	August Froehlich, The Nature Conservancy, afroehlich@TNC.ORG	http://www.glmigratorybirds.org/	Link to download data	NA
**Feature**	Shorebird Habitat	Current stopover habitat	Published spatial data	Great Lakes Migratory Bird Stopover Portal	August Froehlich, The Nature Conservancy, afroehlich@TNC.ORG	http://www.glmigratorybirds.org/	Link to download data	NA

Feature / Cost	Name	Data description	Data Type	Data source	Contact information	Website	Accessibility	Attribution statements
**Feature**	Coastal Landbird Habitat	Current stopover habitat	Published spatial data	Great Lakes Migratory Bird Stopover Portal	August Froehlich, The Nature Conservancy, afroehlich@TNC.ORG	http://www.glmigratorybirds.org/	Link to download data	NA
**Feature**	Public Beaches	Circular polygons of beach locations	Published spatial data	Great Lakes Environmental Assessment and Mapping Project	J.D. Allan, School of Natural Resources & Environment, The University of Michigan, dallan@umich.edu	http://www.greatlakesmapping.org/	Available by request	NA
**Feature**	Birding	Points representing birding hotspots	Published spatial data	Cornell University Lab of Ornithology, eBird	NA	https://confluence.cornell.edu/display/CLOISAPI/eBird-1.1-HotSpotsByRegion	Account required for data download	NA
**Feature**	Birding	Birding observations linked to hotspots	Published spatial data	Cornell University Lab of Ornithology, eBird	NA	http://ebird.org/ebird/eBirdReports?cmd=Start	Account required for data download	NA
**Feature**	Commercial Fishing	Polygons of fisheries statistics grid cells; 10-minute scale in US, 5-minute scale in CAN	Published spatial data	Great Lakes Environmental Assessment and Mapping Project (compiled from the USGS Great Lakes Science Center, and	J.D. Allan, School of Natural Resources & Environment, The University of Michigan, dallan@umich.edu	http://greatlakesmapping.org/great_lake_stressors/3/commercial-fishing	Available by request	NA

Feature / Cost	Name	Data description	Data Type	Data source	Contact information	Website	Accessibility	Attribution statements
				the Ontario Ministry of Natural Resources and Forestry)				
**Feature**	Surface Water Intakes	Point locations of water intake facilities in Ohio	Published spatial data	Ohio Department of Natural Resources, Coastal Management Program, Coastal Atlas	Brian George, Ohio Department of Natural Resources, Brian.George@dnr.state.oh.us	http://coastal.ohiodnr.gov/atlas	Available by request	NA
**Feature**	Surface Water Intakes	Point locations of water intake facilities in Ontario	Unpublished spatial data	Essex Region Conservation Authority	Roger Palmini, RPalmini@erca.org		Available by request	The boundaries of Surface Water Intake Protection Zones as shown are approximate. The location and status of these sites are subject to change. The boundaries of the Surface Water Intake Protection Zones as

Feature / Cost	Name	Data description	Data Type	Data source	Contact information	Website	Accessibility	Attribution statements
								shown are approximate. The location and status of these sites are subject to change. Copyright ERCA 2011
**Feature**	Surface Water Intakes	Point locations of water intake facilities in Michigan	Unpublished spatial data	Michigan Department of Environmental Quality	Jason Berndt, BERNDTJ1@michigan.gov		Available by request	NA
**Feature**	Hunting	Polygons of Michigan public lands open to hunting	Published spatial data	Conservation and Recreation Lands, produced by Ducks Unlimited, attributed as hunting lands according to MI DNR's Mi-hunt online application	NA	http://www.ducks.org/conservation/glaro/carl-gis-layer http://www.michigan.gov/dnr/0,4570,7-153-10363_10913---,00.html	Link to download data	NA
**Feature**	Hunting	Polygons of Ontario public lands	Unpublished spatial data	Shapefiles of protected lands in Federal, Provincial, and	Andrea Hebb, Nature Conservancy Canada, Andrea.Hebb@natureconservancy.ca	NA	Available by request	NA

Feature / Cost	Name	Data description	Data Type	Data source	Contact information	Website	Accessibility	Attribution statements
				private ownership, provided by Nature Conservancy Canada				
**Feature**	Hunting	Polygons of permanently protected areas of Ontario, Canada	Published spatial data	Global Forest Watch Canada, via Data Basin	NA	http://app.databasin.org/app/pages/datasetPage.jsp?id=7982d1126b734bf69e8e5eee82eaf2e0	Link to download data; account may be required	NA
**Feature**	Hunting	List of public areas open to hunting in Ontario	Published tabular data	Essex Region Conservation Authority, list of areas open to hunting	NA	http://erca.org/conservation-areas-events/recreation/hunting/	Link to download data	The location and characterization of these sites as shown are approximate and are subject to change. Copyright ERCA 2012
**Feature**	Hunting	Polygons of hunting areas on Pelee Island	Unpublished spatial data	Nature Conservancy Canada property data	Mhairi McFarlane, Nature Conservancy Canada, Mhairi.McFarlane@natureconservancy.ca	NA	Available by request	NA

Feature / Cost	Name	Data description	Data Type	Data source	Contact information	Website	Accessibility	Attribution statements
**Feature**	Hunting	Polygons of Ohio public lands open to hunting	Published spatial data	Conservation and Recreation Lands, produced by Ducks Unlimited and attributed as hunting lands according to OH DNR's Ohio Wildlife Areas website	NA	http://www.ducks.org/conservation/glaro/carl-gis-layer http://wildlife.ohiodnr.gov/wildlifeareas	Link to download or access data	NA
**Feature**	Parks and Recreation	Polygons of public parks and recreation lands in MI and OH	Published spatial data	Conservation and Recreation Lands, produced by Ducks Unlimited	NA	http://www.ducks.org/conservation/glaro/carl-gis-layer	Link to download data	NA
**Feature**	Parks and Recreation	Polygons of public parks and recreation lands in Ontario	Unpublished spatial data	Provided by Nature Conservancy Canada	Andrea Hebb, Nature Conservancy Canada, Andrea.Hebb@natureconservancy.ca	NA	Available by request	NA
**Feature**	Parks and Recreation	Polygons of conserved lands owned and managed by Essex Region	Unpublished spatial data	Essex Region Conservation Authority	Kevin Money, Essex Region Conservation Authority, Kmoney@erca.org	NA	Available by request	The location and characterization of these sites as shown are approximate

Feature / Cost	Name	Data description	Data Type	Data source	Contact information	Website	Accessibility	Attribution statements
		Conservation Authority						and are subject to change. Copyright ERCA 2012
**Feature**	Parks and Recreation	Polygons of permanently protected areas of Ontario, Canada	Published spatial data	Global Forest Watch Canada, via Data Basin	NA	http://app.databasin.org/app/pages/datasetPage.jsp?id=7982d1126b734bf69e8e5eee82eaf2e0	Link to download data; account may be required	NA
**Feature**	Recreational Boating	Estimated density of boating activity	Published spatial data	Great Lakes Environmental Assessment and Mapping Project	J.D. Allan, School of Natural Resources & Environment, The University of Michigan, dallan@umich.edu	http://greatlakesmapping.org/human-values/8/recreational-boating	Available by request	NA
**Feature**	Shipwreck Dive Sites	point data of shipwreck locations	Published spatial data	Great Lakes Historical Society, as compiled from multiple sources	Carrie Sowden, Archaeological Director for the GL Historical Society, shipwreck@inlandseas.org	http://www.inlandseas.org/	Available by request	NA
**Feature**	Shipwreck Dive Sites	Point data of marina locations	Published spatial data	Great Lakes Environmental Assessment and Mapping Project	J.D. Allan, School of Natural Resources & Environment, The University of Michigan, dallan@umich.edu	http://greatlakesmapping.org/human-values/8/recreational-boating	Available by request	NA

Feature / Cost	Name	Data description	Data Type	Data source	Contact information	Website	Accessibility	Attribution statements
**Feature**	Recreational Fishing	Angler hours attributed to grid cells, MI portion of Lake Erie	Unpublished tabular data	Michigan Department of Natural Resources	Tracy Kolb, Fisheries Biologist, Statewide Angler Survey Program, DNR Fisheries Division, 525 W. Allegan Street, Lansing, MI 48933, KOLBT@michigan.gov	NA	Available by request	NA
**Feature**	Recreational Fishing	Angler hours attributed to grid cells, OH portion of Lake Erie	Unpublished tabular data	Ohio Department of Natural Resources	Travis J. Hartman, Fisheries Biologist, Sandusky Fish Research Unit, 305 East Shoreline Drive, Sandusky, OH 44870, Travis.Hartman@dnr.state.oh.us	NA	Available by request	NA
**Feature**	Recreational Fishing	Angler hours for portions of the Sandusky and Maumee River	Unpublished tabular data	Ohio Department of Natural Resources	Travis J. Hartman, Fisheries Biologist, Sandusky Fish Research Unit, 305 East Shoreline Drive, Sandusky, OH 44870, Travis.Hartman@dnr.state.oh.us	NA	Available by request	NA
**Feature**	Recreational Fishing	Angler hours attributed to grid cells, ON portion of Lake Erie	Published survey data	Belore, M., K. Ho, and R. Drouin. 2008. Summer angler survey in the Canadian waters of the	Megan Belore, Assessment Biologist, Lake Erie Management Unit, Ontario Ministry of Natural Resources, 320 Milo Road, Wheatley, ON, N0P	NA	Available by request	NA

Feature / Cost	Name	Data description	Data Type	Data source	Contact information	Website	Accessibility	Attribution statements
				Western Basin of Lake Erie. Ontario Ministry of Natural Resources: Lake Erie Management Unit.	2P0, megan.belore@ontario.ca			
**Feature**	Recreational Fishing	Angler hours attributed to the ON side of the Detroit River	Published survey data	Soper, K., and B. Locke. 2010. Summer creel census in the Canadian waters of the Detroit River. Ontario Ministry of Natural Resources: Lake Erie Management Unit	Megan Belore, Assessment Biologist, Lake Erie Management Unit, Ontario Ministry of Natural Resources, 320 Milo Road, Wheatley, ON, N0P 2P0, megan.belore@ontario.ca	NA	Data extracted from report table (Table 2, page 16)	NA
**Feature**	Recreational Fishing	Angler hours attributed to the MI side of the Detroit River	Published spatial data	Great Lakes Environmental Assessment and Mapping Project	J.D. Allan, School of Natural Resources & Environment, The University of Michigan, dallan@umich.edu	http://greatlakesmapping.org/great_lake_stressors/3/recreational-fishing	Available by request	NA

Feature / Cost	Name	Data description	Data Type	Data source	Contact information	Website	Accessibility	Attribution statements
**Feature**	Trails	Line data of trails in Ontario	Published spatial data	Ontario Ministry of Natural Resources and Forestry, Land Information Ontario data set, Ontario Trail Network Segment	NA	https://www.javacoeapp.lrc.gov.on.ca/geonetwork/srv/en/main.home	Link to download data	NA
**Feature**	Trails	Line data of trails in Ontario	Published spatial data	Carolinian Canada Coalition's Erie Coastal Stewardship Trail project, received from Nature Conservancy Canada	https://caroliniancanada.ca/contact	http://carolinian.org/coastal-trail	Available by request	NA
**Feature**	Trails	Line data of trails in Michigan	Published spatial data	Southeast Michigan Council of Governments	Brian Pawlik, Southeast Michigan Council of Governments, pawlik@semcog.org	http://www.semcog.org/	Available by request	NA
**Feature**	Trails	Line data of trails in Ohio	Published spatial data	Ohio Department of Natural Resources, Coastal Management	Brian George, Ohio Department of Natural Resources, Brian.George@dnr.state.oh.us	http://coastal.ohiodnr.gov/atlas	Available by request	NA

Feature / Cost	Name	Data description	Data Type	Data source	Contact information	Website	Accessibility	Attribution statements
				Program, Coastal Atlas				
**Feature**	Water Access Sites	Point data for locations of public water access sites in Michigan	Published spatial data	Michigan Department of Natural Resources Boating Access Sites dataset	Chris Kargel, Parks and Recreation Division, Michigan Department of Natural Resources, kargelc@michigan.gov	http://www.mcgi.state.mi.us/mrbis/mapbasic.aspx	Available by request	NA
**Feature**	Water Access Sites	Point data for locations of public water access sites in Ohio	Published spatial data	Ohio Coasta Atlas, Watercraft and Boating Facilities data set	Brian George, Ohio Department of Natural Resources, Brian.George@dnr.state.oh.us	http://coastal.ohiodnr.gov/atlas	Available by request	NA
**Feature**	Water Access Sites	Point data for locations of public water access sites in Ontario	Published spatial data	Carolinian Canada Coalition's Erie Coastal Stewardship Trail project, received from Nature Conservancy Canada	https://caroliniancanada.ca/contact	http://carolinian.org/coastal-trail	Available by request	NA
**Cost**	Land Value	Median home values for Michigan and Ohio	Published tabular data	City-Data.com	NA	http://www.city-data.com/	Link to download data	NA

Feature / Cost	Name	Data description	Data Type	Data source	Contact information	Website	Accessibility	Attribution statements
**Cost**	Land Value	Values for agricultural lands in Ohio	Published report	Farmland Value and Rent Outlook 2013. Ohio State University Extension.	NA	http://ohioagmanager.osu.edu/farm-rents/farmland-value-and-rent-outlook-2013/	Link to access report	NA
**Cost**	Land Value	Value of undeveloped land on Lake Erie islands in Ohio	Personal communication	Lake Erie Islands Conservancy (Formerly Lake Erie Islands Chapter of the Black Swamp Conservancy)	Lisa Kutschbach-Brohl, lakbrohl@gmail.com	http://lakeerieislandsconservancy.org/	Available by request	NA
**Cost**	Land Value	Values for agricultural lands in Michigan	Published report	2012 Michigan Land values and Leasing Rates. Michigan State University Department of Agricultural, Food, and Resource Economics, Report #643.	NA	https://msu.edu/~steind/2012%20MI%20Land%20Values%20Leasing%20Rates%20NO%20643_Final%20Version.pdf	Link to access report	NA
**Cost**	Land Value	Values for urban lands in the Essex region of Ontario	Published report	Online report of residential property values published in	NA	http://hlr.coldwellbanker.com/PressRelease.html	Link to access report	NA

Feature / Cost	Name	Data description	Data Type	Data source	Contact information	Website	Accessibility	Attribution statements
				2013 by Coldwell Bankers				
**Cost**	Land Value	Values for agricultural lands in the Essex region of Ontario	Unpublished report	Ontario land values in the Essex region were estimated based on the Essex Forests and Wetlands Natural Area Conservation Plan (Nature Conservancy Canada, 2009)	Kristen Bernard, Nature Conservancy Canada, Kristen.Bernard@natureconservancy.ca	NA	Available by request	NA
**Cost**	Land value	CCAP landcover 2006	Published spatial data	NOAA's Ocean Service, Office for Coastal Management (OCM), Digital Coast	NA	https://coast.noaa.gov/dataregistry/search/dataset/29B19ED9-7564-4820-9947-937A40793204	Link to download data	NA
**Cost**	Land value	Ontario Provincial Land Cover 28 class	Published spatial data	Ontario Ministry of Natural Resources	NA	https://www.javacoeapp.lrc.gov.on.ca/geonetwork/srv/en/main.home?uuid=ed66d203-d5ca-47a2-b357-	Link to download data	NA

Feature / Cost	Name	Data description	Data Type	Data source	Contact information	Website	Accessibility	Attribution statements
						0226ea3d29a e		
**Cost**	Wetland Restoration Cost	Raster data providing an index of coastal wetland restorability for western Lake Erie (US side only)	Published spatial data	Western Lake Erie Restoration Assessment (WLERA), version 1.0. Western Lake Erie Restoration Assessment	Justin Saarinen, New College of Florida, jsaarinen@ncf.edu	http://wim.usgs.gov/	Link to download data	NA
**Cost**	Wetland Restoration Cost	Polygon shapefile of parcel data for the US	Published spatial data	Parcel Point by CoreLogic	NA	http://www.corelogic.com/products/parcelpoint.aspx	Sensitive data; available only through data use agreement with provider	NA
**Cost**	Wetland Restoration Cost	Polygon shapefile of parcel data for Ontario	Published spatial data	Ontario Ministry of Natural Resources, Land Information Ontario, Digital Assessment Parcel Fabric	NA	https://www.javacoeapp.lrc.gov.on.ca/geonetwork/srv/en/main.home	Link to download data	NA
**Cost**	Wetland Restoration Cost	Surrogate wetland restorability	Unpublished spatial data	Created by The Nature	Gust Annis, The Nature Conservancy, gannis@tnc.org	NA	Available by request	NA

Feature / Cost	Name	Data description	Data Type	Data source	Contact information	Website	Accessibility	Attribution statements
		index for Detroit River and Ontario coast of western Lake Erie		Conservancy for this project				
**Cost**	Phragmites Treatment Cost	Coastal land cover	Published spatial data	Michigan Tech Research Institute	Laura Burgeau-Chavez, lchavez@mtu.edu	http://geodjango.mtri.org/coastal-wetlands/	Available by request	NA
**Cost**	Marinas Cost to Wetlands	Point locations of Great Lakes coastal marinas	Published spatial data	Great Lakes Environmental Assessment and Mapping Project	J.D. Allan, School of Natural Resources & Environment, The University of Michigan, dallan@umich.edu	http://www.greatlakesmapping.org/great_lake_stressors/6/marinas-and-recreational-boating	Available by request	NA
**Cost**	Lake Erie and Detroit River Stress Index	Lake Erie cumulative stressor index	Published spatial data	Great Lakes Environmental Assessment and Mapping Project	J.D. Allan, School of Natural Resources & Environment, The University of Michigan, dallan@umich.edu	http://www.greatlakesmapping.org/lake-stressors	Available by request	NA
**Cost**	Lake Erie and Detroit River Stress Index	Detroit River cumulative stressor index	Unpublished spatial data	Created by The Nature Conservancy for this project via personal communication with J.D. Allan at the	Gust Annis, The Nature Conservancy, gannis@tnc.org	NA	Available by request	NA

Feature / Cost	Name	Data description	Data Type	Data source	Contact information	Website	Accessibility	Attribution statements
				University of Michigan				
**Cost**	Landbird Habitat Restoration Cost	Coastal Land cover	Published spatial data	NOAA Coastal Services Center, C-CAP Regional Land Cover Data	NA	http://greatlakesresilience.org/maps-tools-data/data/coastal-change-analysis-program-c-cap-regional-land-cover-data	Link to download data	NA
**Cost**	Landbird Habitat Restoration Cost	Ontario Provincial Land Cover	Published spatial data	Ontario Ministry of Natural Resources and Forestry	NA	https://www.javacoeapp.lrc.gov.on.ca/geonetwork/srv/en/main.home?uuid=ed66d203-d5ca-47a2-b357-0226ea3d29ae	Link to download data	NA
**Cost**	Landbird Habitat Restoration Cost	Point locations of wind turbines in Ontario	Published spatial data	Ventyx	NA	NA	Sensitive data; available only through data use agreement with provider	NA

Feature / Cost	Name	Data description	Data Type	Data source	Contact information	Website	Accessibility	Attribution statements
**Cost**	Landbird Habitat Restoration Cost	Line file of roads and infrastructure in Ontario	Published spatial data	Produced by the Great Lakes Fishery Commission under Licence with the Ontario Ministry of Natural Resources copyright Queen's Printer for Ontario, 2006,	NA	https://www.javacoeapp.lrc.gov.on.ca/geonetwork?uuid=56156123-9eba-4cc3-864e-bbca5cef72cf	Link to download data	NA
**Cost**	Walleye Stream Habitat Restoration Cost	Line file of streams attributed with habitat value and other parameters	Published spatial data	Midwest Fish Habitat Partnership, Great Lakes Partnership Geodatabase	NA	http://midwestfishhabitats.org/resources/Geodatabase	Link to download data	NA
**Cost**	Walleye Stream Habitat Restoration Cost	Stream connectivity	Unpublished spatial data	Lake Erie Biodiversity Conservation Strategy	Gust Annis, The Nature Conservancy, gannis@tnc.org	https://www.conservationgateway.org/ConservationByGeography/NorthAmerica/wholesystems/greatlakes/Pages/lakeerie.aspx	Available by request	NA

Feature / Cost	Name	Data description	Data Type	Data source	Contact information	Website	Accessibility	Attribution statements
**Cost**	Walleye Stream Habitat Restoration Cost	Line file of Ontario streams	Published spatial data	Ontario Integrated Hydrology Data	NA	https://www.ontario.ca/data/ontario-integrated-hydrology-data	Link to download data	NA
**Cost**	Walleye Stream Habitat Restoration Cost	Watershed health scores	Published report	Essex Region Watershed Report Card	NA	http://erca.org/wp-content/uploads/2013/03/watershed-report-2012-single-pg-for-web.pdf	Link to download report	NA
